# The Skull of *Epidolops ameghinoi* from the Early Eocene Itaboraí Fauna, Southeastern Brazil, and the Affinities of the Extinct Marsupialiform Order Polydolopimorphia

**DOI:** 10.1007/s10914-016-9357-6

**Published:** 2016-10-26

**Authors:** Robin M. D. Beck

**Affiliations:** 1 School of Environment & Life Sciences, University of Salford, M5 4WT, Manchester, UK; 2 School of Biological, Earth and Environmental Sciences, University of New South Wales, Sydney, NSW 2052 Australia

**Keywords:** *Epidolops*, Polydolopimorphia, Marsupialia, Marsupialiformes, Argyrolagidae, Itaboraí, Eocene

## Abstract

The skull of the polydolopimorphian marsupialiform *Epidolops ameghinoi* is described in detail for the first time, based on a single well-preserved cranium and associated left and right dentaries plus additional craniodental fragments, all from the early Eocene (53–50 million year old) Itaboraí fauna in southeastern Brazil. Notable craniodental features of *E. ameghinoi* include absence of a masseteric process, very small maxillopalatine fenestrae, a prominent pterygoid fossa enclosed laterally by a prominent ectopterygoid crest, an absent or tiny transverse canal foramen, a simple, planar glenoid fossa, and a postglenoid foramen that is immediately posterior to the postglenoid process. Most strikingly, the floor of the hypotympanic sinus was apparently unossified, a feature found in several stem marsupials but absent in all known crown marsupials. “Type II” marsupialiform petrosals previously described from Itaboraí plausibly belong to *E. ameghinoi*; in published phylogenetic analyses, these petrosals fell outside (crown-clade) Marsupialia. “IMG VII” tarsals previously referred to *E. ameghinoi* do not share obvious synapomorphies with any crown marsupial clade, nor do they resemble those of the only other putative polydolopimorphians represented by tarsal remains, namely the argyrolagids. Most studies have placed Polydolopimorphia within Marsupialia, related to either Paucituberculata, or to Microbiotheria and Diprotodontia. However, diprotodonty almost certainly evolved independently in polydolopimorphians, paucituberculatans and diprotodontians, and *Epidolops* does not share obvious synapomorphies with any marsupial order. *Epidolops* is dentally specialized, but several morphological features appear to be more plesiomorphic than any crown marsupial. It seems likely *Epidolops* that falls outside Marsupialia, as do morphologically similar forms such as *Bonapartherium* and polydolopids. Argyrolagids differ markedly in their known morphology from *Epidolops* but share some potential apomorphies with paucituberculatans. It is proposed that Polydolopimorphia as currently recognised is polyphyletic, and that argyrolagids (and possibly other taxa currently included in Argyrolagoidea, such as groeberiids and patagoniids) are members of Paucituberculata. This hypothesis is supported by Bayesian non-clock phylogenetic analyses of a total evidence matrix comprising DNA sequence data from five nuclear protein-coding genes, indels, retroposon insertions, and morphological characters: *Epidolops* falls outside Marsupialia, whereas argyrolagids form a clade with the paucituberculatans *Caenolestes* and *Palaeothentes*, regardless of whether the Type II petrosals and IMG VII tarsals are used to score characters for *Epidolops* or not. There is no clear evidence for the presence of crown marsupials at Itaboraí, and it is possible that the origin and early evolution of Marsupialia was restricted to the “Austral Kingdom” (southern South America, Antarctica, and Australia).

**Electronic supplementary material:**

The online version of this article (doi:10.1007/s10914-016-9357-6) contains supplementary material, which is available to authorized users.

## Introduction

Calcareous deposits in the Itaboraí Basin in Rio de Janeiro State, southeastern Brazil, preserve one of the few diverse early Palaeogene vertebrate faunas known from South America outside Patagonia (Bergqvist et al. 2008). The Itaboraí fauna formed the basis for recognizing the Itaboraian South American Land Mammal Age (SALMA; Gelfo et al. 2009; Woodburne et al. 2014b). The absolute age of the Itaboraian has been difficult to resolve: older papers typically interpreted it as Paleocene (Marshall 1985; Medeiros and Bergqvist 1999), but more recent works have proposed a younger age, namely Paleocene-Eocene or early Eocene (Marshall et al. 1997; Gelfo et al. 2009; Woodburne et al. 2014b; Goin et al. 2016, in press). The most recent published estimate for the absolute age of the Itaboraian is 50–53 MYA (Woodburne et al. 2014b).

Among the vertebrate fossils collected from Itaboraí are hundreds of mammal specimens. Most of these are isolated teeth and jaw fragments (Paula Couto 1952a, b, c, d; Cifelli 1983b; Marshall 1987; Oliveira and Goin 2006, 2011; Goin and Oliveira 2007; Goin et al. 2009), but postcranial (Paula Couto 1952a; Cifelli 1983a, b; Szalay 1994; Szalay and Sargis 2001; Bergqvist et al. 2004; Bergqvist 2008; Oliveira et al. 2016) and cranial (Paula Couto 1952a, b, c, d; Ladevèze 2004, 2007; Ladevèze and Muizon 2010; Oliveira and Goin 2015) remains are also present. The mammalian fauna comprises both eutherian and marsupialiform species, and is highly diverse, with more than 25 genera currently recognized (Oliveira and Goin 2006, 2011; Bergqvist 2008; Woodburne et al. 2014a). However, representatives of non-therian lineages known to have survived into the Cenozoic in South America (namely monotremes, meridiolestidans, and gondwanatherians; Pascual et al. 1992, 1999; Gelfo and Pascual 2001; Goin et al. 2012b; Rougier et al. 2012) have not been described from Itaboraí.

Several Itaboraí taxa are candidates for being the oldest putative crown marsupials known from South America, notably the apparent paucituberculatan *Riolestes capricornensis* (if this is not based on a deciduous premolar of another taxon; see Goin et al. 2009; Beck in press-b), plus isolated marsupialiform tarsals that Szalay (1994) referred to his “Itaboraí Metatherian Groups” (IMGs) V and XII and which have been identified as possibly representing early didelphimorphians or another crown marsupial lineage (Szalay 1994; Szalay and Sargis 2001; Beck in press-b).

One of the best preserved marsupialiform fossils from Itaboraí is DGM 321-M, a crushed partial cranium and associated left and right mandibles of *Epidolops ameghinoi* (Paula Couto 1952c; Marshall 1982a: figs. 62–63; Bergqvist et al. 2008: fig. 9B). *Epidolops ameghinoi* is also represented by more than one hundred additional craniodental fragments from Itaboraí (Marshall 1982a: 74–82), making it by far the most abundant marsupialiform in the fauna. In his original description of *Epidolops*, Paula Couto (1952c) identified a second species, *E. gracilis*, among the Itaboraí material. However, Marshall (1982a) considered that all the specimens could be referred to a single species, *E. ameghinoi*. Subsequently, Szalay (1994) tentatively referred isolated tarsals comprising his IMG VII morphotype to *E. ameghinoi*.

*Epidolops* is a member of the extinct order Polydolopimorphia (Case et al. 2005; Goin et al. 2009, 2016, in press). Polydolopimorphians are usually described as having a diprotodont lower dentition, sometimes referred to as “pseudodiprotodont” on the assumption that it is non-homologous with that of diprotodontians (Ride 1962, 1964; Goin 2003; Goin et al. 2009). Most polydolopimorphians exhibit a relatively low-crowned, bunodont molar morphology (Marshall 1982a; Goin 2003; Chornogubsky 2010; Goin et al. in press), but the Oligocene-Pliocene argyrolagoids include forms with hypsodont and hypselodont molars (Simpson 1970b; Hoffstetter and Villarroel 1974; Pascual and Carlini 1987; Villarroel and Marshall 1988; Sánchez-Villagra and Kay 1997; Flynn and Wyss 1999; Sánchez-Villagra et al. 2000; Goin et al. 2010, in press; Zimicz 2011).

Given current definitions of the order, the South American fossil record of Polydolopimorphia spans from the Paleocene to the Pliocene (Goin et al. 2016, in press). Polydolopimorphians are also known from the middle Eocene La Meseta Fauna from Seymour Island, off the Antarctic Peninsula (Woodburne and Zinsmeister 1982, 1984; Goin et al. 1999; Chornogubsky et al. 2009). Possible polydolopimorphians have been described from the Late Cretaceous of North America (Case et al. 2005) and the Cenozoic of Australia (Beck et al. 2008a; Sigé et al. 2009), but these more questionable records are based on very fragmentary dental evidence; their similarities may simply reflect convergent evolution of a bunodont molar morphology (Beck et al. 2008a).

Recent works (e.g., Case et al. 2005; Goin et al. 2010, 2016, in press; Oliveira and Goin 2011; Chornogubsky and Goin 2015) have recognized three suborders within Polydolopimorphia (see Table 1): Hatcheriformes (which contains the dentally most plesiomorphic forms); Polydolopiformes (which includes *Roberthoffstetteria nationalgeographica* from the early or middle Paleocene Tiupampa locality in Bolivia, *Sillustania quechuense* from the late Paleocene-early Eocene Chulpas locality in Peru, and the diverse polydolopids) and Bonapartheriiformes. Within Bonapartheriiformes, two superfamilies are currently recognized: Bonapartherioidea and Argyrolagoidea (the latter including the dentally highly derived groeberiids, patagoniids, and argyrolagids; Goin et al. 2010, 2016, in press; Zimicz 2011). Goin et al. (2016: table 5.1) considered *Epidolops* to be a member of Bonapatherioidea, within which they recognized four families: Prepidolopidae, Bonapartheriidae, Gashterniidae, and Rosendolopidae. Goin et al. (2016: table 5.1) placed *Epidolops* in Bonapartheriidae, but in its own subfamily, namely Epidolopinae (see also Goin et al. 2003a, in press; Goin and Candela 2004; Case et al. 2005).

**Table 1:** Selected metatherian classifications, modified from Goin et al. (2009: table 2). As presented here, the classifications other than those of Simpson (1945) and Ride (1964) are restricted to metatherians from North and South America only, and taxonomic levels below the level of family are not shown, except that the bonapatheriid subfamilies Epidolopinae (which includes *Epidolops*) and Bonapartheriinae are indicated for the classifications of Case et al. (2005) and Goin et al. (2016)

Simpson (1945)	Ride (1964)	Aplin & Archer (1987)	Marshall et al. (1990)	Szalay (1994)	Kirsch et al. (1997)	Case et al. (2005)	Goin et al. (2016)
Order Marsupialia	Infraclass Metatheria	Supercohort Marsupialia	Infraclass Metatheria	Infraclass Metatheria	Infraclass Marsupialia	Supercohort Marsupialia	Infraclass Metatheria
Superfamily Didelphoidea	Superorder Marsupialia	Cohort Ameridelphia	Supercohort Marsupialia	Cohort Ameridelphia	Supercohort Boreometatheria	Cohort Alphadelphia	Cohort “Ameridelphia”
Family Didelphidae	Order Marsupicarnivora	Order Didelphimorphia	Cohort Alphaldelphia	Order Didelphida	Order Peradectimorphia	Order Peradectia	Family Pediomyidae
Family Caroloameghiniidae	Superfamily Didelphoidea	Family Didelphidae	Order Peradectia	Suborder Archimetatheria	Family Peradectidae	Family Peradectidae	Family Pucadelphyidae
Superfamily Borhyaenoidea	Family Didelphidae	Family Sparassocynidae	Cohort Ameridelphia	Family Stagodontidae	Supercohort Notometatheria	Family Pediomyidae	Family Jaskhadelphyidae
Family Borhyaenidae	Superfamily Borhyaenoidea	Order Paucituberculata	Order Didelphimorphia	Family Pediomyidae	Cohort Didelphidia	Family Stagodontidae	Family Mayulestidae
Superfamily Dasyuroidea	Family Borhyaenidae	Superfamily Caroloameghinioidea	Superfamily Didelphoidea	Suborder Sudameridelphia	Order Didelphimorphia	Family Caroloameghiniidae	Family Protodidelphidae
Family Dasyuridae	Superfamily Dasyuroidea	Family Caroloameghiniidae	Family Didelphidae	Infraorder Itaboraiformes	Family Didelphidae	Cohort Ameridelphia	Family Derorhynchidae
Family Notoryctidae	Family Dasyuridae	Superfamily Caenolestoidea	Family Sparassocynidae	Family Caroloameghiniidae	Cohort Pseudiprotodontia	Order Didelphimorphia	Family Sternbergiidae
Superfamily Perameloidea	Family Thylacinidae	Family Caenolestidae	Order Polydolopimorphia	Infraorder Polydolopimorphia	Order Paucituberculata	Superfamily Didelphoidea	Family Herpetotheriidae
Family Peramelidae	Order Paucituberculata	Superfamily Argyrolagoidea	Superfamily Polydolopoidea	Family Prepidolopidae	Superfamily Caenolestoidea	Family Pucadelphidae	Order Sparassodonta
Superfamily Caenolestoidea	Family Caenolestidae	Family Gashterniidae	Family Protodidelphidae	Family Polydolopidae	Family Caenolestidae	Family “Didelphidae”	Family Hondadelphidae
Family Caenolestidae	Family Polydolopidae	Family Groeberiidae	Family Prepidolopidae	Family Bonapartheriidae	Superfamily Argyrolagoidea	Family Caluromyidae	Family Hathliacynidae
Family Polydolopidae	Order Peramelina	Family Argyrolagidae	Family Bonapartheriidae	Infraorder Sparassodonta	Family Argyrolagidae	Family Herpetotheriidae	Superfamily Borhyaenoidea
Superfamily Phalangeroidea	Family Peramelidae	Superfamily Polydolopoidea	Family Polydolopidae	Family Borhyaenidae	Family Patagoniidae	Family Derorhynchidae	Family Borhyaenidae
Family Phalangeridae	Order Diprotodonta	Family Prepidolopidae	Order Sparassodonta	Family Thylacosmilidae	Superfamily Groeberioidea	Family Protodidelphidae	Family Proborhyaenidae
Family Thylacoleonidae	Family Phalangeridae	Family Bonapartheriidae	Superfamily Borhyaenoidea	Suborder Glirimetatheria	Family Groeberiidae	Order Sparassodonta	Family Thylacosmilidae
Family Phascolomidae	Family Wynyardidae	Family Polydolopidae	Family Stagodontidae	Infraorder Paucituberculata	Order Polydolopimorphia	Superfamily Borhyaenoidea	Supercohort Marsupialia
Family Macropodidae	Family Vombatidae	Order Sparassodonta	Family Hondadelphidae	Family Caenolestidae	Superfamily Polydolopoidea	Family Mayulestidae	Order Didelphimorphia
Family Diprotodontidae	Family Diprotodontidae	Family Borhyaenidae	Family Hathliacynidae	Infraorder Simpsonitheria	Family Polydolopidae	Family Hondadelphidae	Superfamily Peradectoidea
	Family Macropodidae	Family Thylacosmilidae	Family Borhyaenidae	Family Gashterniidae	Family Prepidolopidae	Family Hathliacynidae	Family Peradectidae
	Marsupialia *incertae sedis*	Cohort Australidelphia	Family Proborhyaenidae	Family Groeberiidae	Family Bonapartheriidae	Family Borhyaenidae	Family Caroloameghiniidae
	Family Notoryctidae	Order Microbiotheria	Family Thylacosmilidae	Family Argyrolagidae	Family Protodidelphidae	Family Proborhyaenidae	Superfamily Didelphoidea
		Family Microbiotheriidae	Order Paucituberculata	Family Patagoniidae	Superfamily Caroloameghinoidea	Family Thylacosmilidae	Family Didelphidae
			Superfamily Caenolestoidea	Suborder Didelphimorphia	Family Caroloameghinidae	Order Paucituberculata	Family Sparassocynidae
			Family Kollpaniidae	Family Didelphidae	Cohort Eometatheria	Cohort Australidelphia	Order Paucituberculata
			Family Caenolestidae	Family Sparassocynidae	Order Microbiotheria	Order Microbiotheria	Superfamily Caenolestoidea
			Family Palaeothentidae	Cohort Australidelphia	Family Microbiotheriidae	Family Microbiotheriidae	Family Caenolestidae
			Family Abderitidae	Order Gondwanadelphia		Marsupialia *incertae sedis*	Superfamily Palaeothentoidea
			Superfamily Argyrolagoidea	Suborder Microbiotheria		Order Polydolopimorphia	Family Pichipilidae
			Family Argyrolagidae	Family Microbiotheriidae		Suborder Hatcheriformes	Family Palaeothentidae
			Family Groeberiidae			Suborder Bonapartheriiformes	Family Abderitidae
			Cohort Australidelphia			Superfamily Bonapartherioidea	Cohort Australidelphia
			Order Microbiotheria			Family Prepidolopidae	Order Microbiotheria
			Superfamily Microbiotheroidea			Family Bonapartheriidae	Family Woodburnodontidae
			Family Pediomyidae			Subfamily Bonapartheriinae	Family Microbiotheriidae
			Family Microbiotheriidae			Subfamily Epidolopinae	Order Polydolopimorphia
						Family Gashterniidae	Family Glasbiidae
						Superfamily Argyrolagoidea	Suborder Bonapartheriiformes
						Family Groeberiidae	Superfamily Bonapartherioidea
						Family Patagoniidae	Family Prepidolopidae
							Family Bonapartheriidae
							Subfamily Bonapartheriinae
							Subfamily Epidolopinae
							Family Gashterniidae
							Family Rosendolopidae
							Superfamily Argyrolagoidea
							Family Groeberiidae
							Family Patagoniidae
							Family Argyrolagidae
							Suborder Polydolopiformes
							Family Polydolopidae

Goin et al.‘s (2016) classification of Polydolopimorphia received partial support from the phylogenetic analyses of Goin et al. (2009) and Chornogubsky and Goin (2015): in both analyses, clades equivalent to Polydolopiformes*,* Bonapartheriiformes, and Argyrolagoidea were recovered. The unpublished phylogenetic analyses of Chornogubsky (2010), meanwhile, recovered clades equivalent to Polydolopiformes and Bonapartheriiformes, but these analyses were focused on relationships within Polydolopidae and included only four non-polydolopid polydolopimorphians (*Epidolops*, *Bonapartherium*, *Prepidolops*, and *Roberthoffstetteria*). Ultimately, taxon sampling in these and other analyses (e.g., Goin et al. 2006; Oliveira and Goin 2011; Forasiepi et al. 2013) is too limited to adequately test relationships within Polydolopimorphia.

The relationship of polydolopimorphians to other marsupialiforms has proved difficult to resolve (summarized in Table 1). Most early studies argued for a close relationship between Polydolopimorphia and the South American order Paucituberculata (which includes the living caenolestid “shrew opossums”), largely based on the shared presence of diprotodonty (Gregory 1910; Simpson 1928, 1945, 1948; Paula Couto 1952c). In a major review of “Polydolopidae” (= polydolopids and *Epidolops*), Marshall (1982a) concluded that the enlarged anterior “gliriform” tooth of the lower jaw of polydolopids is probably the canine. If so, diprotodonty must have arisen independently in polydolopids and paucituberculatans, because the paucituberculatan gliriform tooth is unequivocally an incisor (Ride 1962; Abello 2013). Some authors that accepted Marshall’s (1982a) conclusion that the polydolopid gliriform tooth is the lower canine nevertheless continued to link polydolopimorphians with paucituberculatans (e.g., Aplin and Archer 1987; Marshall 1987; Kirsch et al. 1997). Kirsch et al. (1997) named the grouping of Polydolopimorphia and Paucituberculata as the cohort Pseudiprotodontia. The classifications of Aplin and Archer (1987), Marshall (1987), and Kirsch et al. (1997) did not group argyrolagoids or gashterniids with other polydolopimorphians, with Kirsch et al. (1997) instead placing them within Paucituberculata.

The classifications of Marshall et al. (1990), Szalay (1994), and Case et al. (2005), by contrast, did not endorse a specific relationship between Polydolopimorphia and Paucituberculata. In Marshall et al.‘s (1990: fig. 2) phylogeny, Polydolopimorphia is sister to Didelphimorphia (which includes living didelphid opossums), whilst Paucituberculata is sister to Sparassodonta (an extinct order of South American carnivorous marsupialiforms), with these four orders collectively forming a clade. Marshall et al. (1990) referred to this clade as Ameridelphia, which is a name originally proposed by Szalay (1982) to refer to non-australidelphian marsupialiforms. Marshall et al. (1990) placed the argyrolagoid families Groeberiidae and Argyrolagidae within Paucituberculata, rather than Polydolopimorphia.

Szalay (1994) classified Polydolopimorphia as an infraorder in his suborder Sudameridelphia, and recognized Paucituberculata as an infraorder within a different suborder, Glirimetatheria. Szalay (1994) also erected the infraorder Simpsonitheria for the argyolagoid families Groeberiidae, Argyrolagidae, and Patagoniidae, plus Gashterniidae. Szalay (1994) placed Simpsonitheria together with Paucituberculata, in Glirimetatheria. Finally, Case et al. (2005) classified Polydolopimorphia (including argyrolagoids and gashterniids) as “Marsupialia” (= Marsupialiformes here) *incertae sedis*, but placed Paucituberculata together with Didelphimorphia and Sparassodonta in Ameridelphia.

In several papers (Goin et al. 1998b, 2009, 2016, in press; Goin 2003; Goin and Candela 2004; Oliveira and Goin, 2006, 2011; Chornogubsky and Goin 2015), Goin and co-authors have proposed a very different hypothesis of polydolopimorphian relationships. Specifically, they have argued that Polydolopimorphia is closely related to the order Microbiotheria, which is known from South America (including the extant *Dromiciops gliroides*) and the middle Eocene of Seymour Island off the Antarctic Peninsula, and the Australian order Diprotodontia, which includes the koala, wombats, “possums,” kangaroos, and a range of extinct forms. Recently, isolated tarsals from the early-middle Eocene (Lutetian) La Barda locality in Patagonia have also been identified as representing a probable diprotodontian (Lorente et al. 2016); if so, this is the first South American record of Diprotodontia. If polydolopimorphians are close relatives of microbiotherians and diprotodontians, it would mean that they are also members of the trans-Gondwanan marsupial superorder Australidelphia (Szalay 1982, 1994; Beck et al. 2008b; Nilsson et al. 2010; Beck 2012, in press-b), which in turn would have significant implications for our understanding of marsupialiform biogeography.

To date, hypotheses regarding polydolopimorphian affinities have relied almost exclusively on dental features (Goin 2003; Goin et al. 2006, 2009; Oliveira and Goin 2011). Cranial anatomy is obviously a key source of phylogenetic (as well as functional) data within mammals, but few crania of polydolopimorphians are known. Several relatively complete crania of argyrolagids have been described (Simpson 1970b; Sánchez-Villagra and Kay 1997; Sánchez-Villagra et al. 2000), but these represent relatively late (Oligocene or younger), craniodentally apomorphic taxa. The groeberiid *Groeberia* is also known from partial crania, but these are less well preserved, and the known craniodental morphology of this taxon is also highly apomorphic (Patterson 1952; Simpson 1970a; Pascual et al. 1994). Among older polydolopimorphians, the skull of *Epidolops ameghinoi* from Itaboraí, DGM-321-M, is one of the best preserved, and is therefore a critically important specimen. However, despite having been illustrated in several published works (Paula Couto 1952c; Marshall 1982a: figs. 62–63; Bergqvist et al. 2008: fig. 9B), DGM 321-M has never been described in detail.

In this paper, I provide the first detailed description of the cranial morphology of *Epidolops ameghinoi*, based largely on DGM 321-M but supplemented by information provided by the additional specimens from Itaboraí. I do not present a detailed description of the dentition, because this has been well covered in previous publications (Marshall 1982a; Goin and Candela 1996; Zimicz 2014), but I present a novel interpretation for the dental formula of *E. ameghinoi*. I argue that the marsupialiform “Type II” petrosals described by Ladevèze (2004) plausibly belong to *E. ameghinoi*, and I accept that the IMG VII tarsals referred to this taxon by Szalay (1994) are correctly attributed. I compare the morphology of *Epidolops* with that of other taxa currently included in Polydolopimorphia. I qualitatively assess the available morphological evidence regarding the relationship of Polydolopimorphia to other marsupialiforms. I discuss the implications of the new information presented here for our understanding of the affinities of argyrolagids and other argyrolagoids. As a quantitative test of the position of polydolopimorphians within Metatheria, I add *Epidolops* and the argyrolagids *Argyrolagus* and *Proargyrolagus* to modified versions of the total evidence matrix of Beck et al. (2014) and analyze them using a Bayesian undated approach. I conclude with a discussion of the implications of this study for our understanding of marsupialiform biogeography, specifically regarding the origin and early evolution of Marsupialia.

## Materials and Methods

### Specimens

All specimens of *Epidolops ameghinoi* that I examined are currently housed at the Museu de Ciências da Terra (prefix DGM) in the Departamento Nacional de Produção Mineral, and at the Museu Nacional do Rio de Janeiro (prefix MNRJ), both in Rio de Janeiro. Comparative specimens of other taxa examined in the course of this study are from the Department of Mammalogy at the American Museum of Natural History (prefix AMNH M-), the University of New South Wales (prefix UNSW), the Museo Municipal de Ciencias Naturales “Lorenzo Scaglia,” Mar del Plata (prefix MMP), and the Natural History Museum, London (prefix BMNH).

### Anatomical Terminology and Abbreviations

Terminology for cranial anatomy follows Beck et al. (2014; see also Wible 2003; Voss and Jansa 2009). Terminology and abbreviations for the dental formula follow Voss and Jansa (2009: table 7), in which the maximum metatherian dental formula is assumed to be I1–5 C1 P1–3 M1–4 in the upper dentition and i1–4 c1 p1–3 m1–4 in the lower dentition. Recent papers by Goin and co-authors (e.g., Oliveira and Goin 2011) have instead followed Hershkovitz (1982, 1995) in assuming that metatherians have lost the anteriormost lower incisor, and so have referred to the lower incisors as i2–5 (see also Voss and Jansa 2009: table 7).

### Assumed Classification

I tentatively follow Goin et al.’s (2016) classification of *Epidolops* within Polydolopimorphia (see also Goin and Candela 2004; Case et al. 2005; Goin et al. 2010, in press). However, the results of the current study cast doubt on whether argyrolagids (and possibly other argyrolagoids) are polydolopimorphians; I believe it more likely that argyrolagids are in fact members of Paucituberculata (see below). I follow Sereno’s (2006: table 10.1) stem-based phylogenetic definition for Metatheria, namely the most inclusive clade containing *Didelphis marsupialis* but not *Mus musculus*. I restrict the name Marsupialia to the crown-clade only (see Rougier et al. 1998; Flynn and Wyss 1999), and I use the phylogenetic definition of Beck et al. (2014: 131), namely the least inclusive clade containing *Didelphis marsupialis*, *Caenolestes fuliginosus*, and *Phalanger orientalis*. Vullo et al. (2009) proposed the name Marsupialiformes for the clade corresponding to “traditional,” more inclusive definitions of Marsupialia (e.g., Kielan-Jaworowska et al. 2004); I follow Beck’s (in press-a: Table 1) definition of Marsupialiformes here, namely the most inclusive clade containing *Didelphis marsupialis* but not *Deltatheridium pretrituberculare*.

### Regression Analysis of Petrosal Size

Ladevèze and Muizon (2010) ruled out referral any of the eight marsupialiform petrosal morphotypes (Types I-VIII) described from Itaboraí to *E. ameghinoi* based on incompatibilty in relative size. However, Ladevèze and Muizon (2010) based this conclusion on regressions of molar area (for M2, M3, m2, and m3) against promontorium area, and Szalay (1994: Table 6.3) remarked that *E. ameghinoi* has “relatively small molars [that] are unlikely to reflect body size accurately.” As an alternative approach, I regressed promontorium area against total cranial length for the set of 12 extant and fossil marsupialiform taxa used by Ladevèze and Muizon (2010: table 2). I then plotted estimated skull length for *E. ameghinoi* (55 mm – see below) and promontorium area for the eight Itaboraí petrosal morphotypes to see if any of the eight morphotypes is an appropriate size for referral to *E. ameghinoi*. Following Beck (2012), all measurements were log_10_-transformed prior to analysis, and reduced major axis regression was used (as implemented by the R package smatr; Warton et al. 2012; R Development Core Team 2016). Measurements and sources for these are given in the Electronic Supplementary Material.

### Phylogenetic Analysis

As a test of the evolutionary relationships of *Epidolops* and argyrolagids, I carried out a phylogenetic analysis using modified versions of the total evidence matrix of Beck et al. (2014). This matrix comprises DNA sequence data from five nuclear protein-coding genes (*APOB*, *BRCA1*, *IRBP*, *RAG1*, and *VWF*), plus indels in the sequence data, retroposon insertions, and morphological characters (see Beck et al. 2014 for full details). This dataset was enlarged by adding 20 retroposon insertion characters taken from Gallus et al. (2015), and 15 novel morphological characters. *Epidolops* and the argyrolagids *Proargyrolagus* and *Argyrolagus* were then added to this expanded matrix.

I produced two versions of the matrix: in the first (“Matrix A”), I scored *Epidolops* based solely on DGM 321-M and other isolated craniodental specimens from Itaboraí that could be unequivocally identified as belonging to this taxon based on dental morphology; in the second (“Matrix B″), I assumed that the Type II petrosal morphotype described by Ladevèze (2004) and the IMG VII tarsal morphotype described by Szalay (1994) also represent *Epidolops*, and used these additional specimens for scoring purposes (see below). Scores for *Epidolops* were based on firsthand observation of craniodental specimens in the DGM and MNRJ collections, plus the descriptions of the Type II petrosals by Ladevèze (2004) and the IMG VII tarsals by Szalay (1994). Scores for *Argyrolagus* were taken from Simpson (1970b), whilst those for *Proargyrolagus* were taken from Sánchez-Villagra and Kay (1997), Sánchez-Villagra et al. (2000), and Sánchez-Villagra (2001). A full list of the morphological characters and scorings for *Epidolops*, *Argyrolagus*, and *Proargyrolagus* is given in Electronic Supplementary Material. The full morphological and total evidence matrices can be downloaded from Morphobank (http://www.morphobank.org, Project 2436).

The complete total evidence matrix was analyzed using a Bayesian non-clock approach in MrBayes 3.2.6, following Beck et al. (2014). As in Beck et al. (2014), an eight partition scheme was used for the DNA sequence data, and the nuclear indel and retroposon insertion partitions were assigned separate restriction site (binary) models, with the assumption that only variable characters were coded. For the morphological partition, an Mk model was specified; because autapomorphies were present, I specified that variable characters were scored (“coding = var”). As in Beck et al. (2014), a gamma distribution with four rate categories was used to to model rate heterogeneity between morphological characters.

The MrBayes 3.2.6 analysis comprised two independent runs of four chains (three “heated,” one “cold”), running for 50 × 10^6^ generations and sampling trees every 2000 generations. The temperature of the heated chains was decreased from 0.2 to 0.1. An average standard deviation of split frequencies of 0.01–0.02 indicated that the chains had converged. The first 25 % were discarded as burn-in. A minimum ESS of >500 and PSRF of 1.00 for all parameters confirmed that stationarity was reached among the post-burn-in trees, as also indicated by plots of log likelihood against generation number. 50 % majority rule consensus was used to summarize the post-burn-in trees, with Bayesian posterior probabilities (BPPs) calculated as support values.

SYSTEMATIC PALEONTOLOGY

METATHERIA HUXLEY, 1880 (SENSU SERENO, 2006)

MARSUPIALIFORMES VULLO ET AL., 2009 (SENSU BECK, IN PRESS-A)

POLYDOLOPIMORPHIA ARCHER, 1984

BONAPARTHERIIFORMES PASCUAL, 1980

BONAPARTHERIOIDEA PASCUAL, 1980

BONAPARTHERIIDAE PASCUAL, 1980

EPIDOLOPINAE PASCUAL AND BOND, 1981

*EPIDOLOPS* PAULA COUTO, 1952

*EPIDOLOPS AMEGHINOI* PAULA COUTO, 1952

### Diagnosis

Marshall (1982: 73–74) presented a detailed but non-differential diagnosis for the subfamily Epidolopinae, which was based on *E. ameghinoi* only, but a differential diagnosis is presented here.

Medium-sized marsupialiform (estimated body mass ~ 400 g; Zimicz 2014) with probable dental formula I1–3/i1–3 C1/c1 P1–3/p1–3 M1–4/m1–4. Differs from most marsupialiforms in the combined presence of diprotodonty, enormous and plagiaulacoid P3 and p3, and bunodont molars. Differs from paucituberculatans in that its i2–3 and c1 are well developed and procumbent (rather than reduced and single-rooted or absent), a large diastema is present behind c1, P3 and p3 are enormous and plagiaulacoid, maxillopalatine fenestrae are very small, and an alisphenoid tympanic process is absent. Differs from diprotodontians in that its i2–3 and c1 are well developed and procumbent (rather than reduced and single-rooted or absent), the floor of its hypotympanic sinus is unossified, its glenoid fossa is simple and planar (rather than complex, with a separate articular eminence and mandibular fossa), and its postglenoid foramen is posterior to the postglenoid process (rather than shifted medially).

Among taxa currently included by Goin et al. (2016) in Polydolopimorphia, *E. ameghinoi*: differs from hatcheriforms in that stylar cusp C is absent, stylar cusp B and stylar cusp D are positioned relatively closer to the paracone and metacone, respectively, and the paracone and metacone are connected by weak lophs to the protocone and metaconular hypocone, respectively; differs from polydolopiforms in having better developed molar crests and in lacking a well-developed paraconule and well-developed supernumerary cusps; differs from rosendolopids in that stylar cusp B and stylar cusp D are positioned relatively closer to the paracone and metacone, respectively, and the paracone and metacone are connected by weak lophs to the protocone and metaconular hypocone, respectively; differs from prepidolopids in that P3 and p3 are plagiaulacoid (with a distinct serrated edge), in having a more procumbent anterior dentition, in having stylar cusp B and stylar cusp D positioned relatively closer to the paracone and metacone, respectively, and in having paracone and metacone connected by weak lophs to the protocone and metaconular hypocone, respectively; differs from *Gashternia* in that its P3 has many more cuspules forming a serrated edge and lacks a lingual shelf; differs from *Bonapartherium* in that P2 is much smaller and single-rooted, P3 is plagiaulacoid and lacks a lingual platform, stylar cusp B and stylar cusp D are positioned relatively further from the paracone and metacone, respectively, and the lower incisors are enlarged and procumbent; differs from *Patagonia* in that its i1 is more procumbent, its i2–3 and c1 are well developed and procumbent (rather than reduced and single-rooted or absent), its P3 and p3 are enormous and plagiaulacoid (rather than absent), its molars are bunodont (rather than hypsodont), and a total of four (rather than three) molars are present; differs from *Groeberia* in that its i1 is more procumbent, its i2–3 and c1 are well developed and procumbent (rather than reduced and single-rooted or absent), its P3 and p3 are enormous and plagiaulacoid (rather than very reduced or absent), its molars are bunodont (rather than hypsodont), its rostrum is relatively longer, it lacks a distinct masseteric process, its maxillopalatine fenestrae are much smaller, and its dentary is relatively longer and shallower and lacks a medial platform; differs from *Klohnia* in that its i1 is more procumbent, its i2–3 and c1 are well developed and procumbent (rather than reduced and single-rooted or absent), its molars are bunodont (not hypsodont), and a total of four (rather than three) molars are present; differs from argyrolagids in that its i1 is more procumbent, its i2–3 and c1 are well developed and procumbent (rather than reduced and single-rooted or absent), its molars are bunodont (not hypsodont or hypselodont), a large diastema is present behind c1, P3 and p3 are enormous and plagiaulacoid (rather than small and hypsodont or hypselodont, or entirely absent), maxillopalatine fenestrae are present but very small, and an alisphenoid tympanic process is absent.

### Distribution and Temporal Range

All known specimens of *E. ameghinoi* are from the Itaboraí Basin, Rio de Janeiro State, southeastern Brazil (Paula Couto 1952c; Marshall 1982a). Three distinct depositional phases, S1–3, have been recognized at Itaboraí (Medeiros and Bergqvist 1999; Bergqvist et al. 2008); all *E. ameghinoi* specimens are reported as being from fissure fills (Paula Couto 1952c; Marshall 1982a), which formed during phase S2. The absolute age of the Itaboraí fauna (Itaboraian SALMA) remains somewhat uncertain and it seems likely that the fossil-bearing deposits span a considerable age range (Gayet et al. 1991; Marshall et al. 1997; Rage 1998; Pinheiro et al. 2012). An ankaramite flow at the northern border of the Itaboraí Basin has a K/Ar date of 52.6 +/− 2.4 Ma (Riccomini and Rodrigues-Francisco 1992) and may postdate phase S2 (Bergqvist et al. 2008). Another Itaboraian fauna is known from the Las Flores Formation in central Patagonia (Goin et al. 1997; Woodburne et al. 2014b); Ar/Ar dating of an overlying tuff suggests a minimum age of 49.5 Ma for this fauna (Woodburne et al. 2014b). Based largely on these two radiometric dates, Woodburne et al. (2014b) suggested that the Itaboraian spans ~53–50 Ma, and I tentatively follow this here.

### Notes

A smaller species, *E. redondoi* (estimated body mass 127 g; Zimicz 2014: tabla 4), has been described from the Cerro Redondo locality in Patagonia (Goin and Candela 1995). Simpson (1935) identified 14 stratigraphic levels (a-n) at Cerro Redondo and reported that mammals were found in levels h and m. Simpson (1935) concluded that level h was slightly older than the *Carodnia* Zone of the Peñas Coloradas Formation, which is currently interpreted as 62 Ma old (Clyde et al. 2014; Woodburne et al. 2014b). Woodburne et al. (2014a: fig. 6) proposed that the stratigraphically higher level m at Cerro Redondo falls within the *Ernestokokenia* Faunal Zone, which represents the Riochican SALMA, currently estimated at ~49 Ma old (Woodburne et al. 2014b). It is uncertain from which level at Cerro Redondo the only known specimen of *E. redondoi* was collected (Goin and Candela 1995); however, assuming that is from one of the two levels known to bear mammals, and that an age of 50–53 Ma for the Itaboraían is accurate, it seems more likely that it is from level m and hence is 49 Ma old.

Gayet et al. (1991) mentioned the presence of *Epidolops* sp. at Estancia Blanco Rancho, Santa Lucia Formation, Bolivia. If Estancia Blanco Rancho is similar in age to a much better known Santa Lucia Formation fauna, Tiupampa, it is probably early or middle Paleocene in age (Marshall et al. 1997; Woodburne et al. 2014b). However, this would mean the *Epidolops* material from Estancia Blanco Rancho is at least 9 Myr older than that from Itaboraí; as such, this record should be treated with caution pending a description of the relevant material.

Two as-yet undescribed species of *Epidolops* listed by Zimicz (2014: 108) are from the Itaboraian-aged Las Flores Formation of Patagonia (F.J. Goin, pers. comm.).

## Description

### Overall Morphology of DGM-321 M

**Fig. 1:**
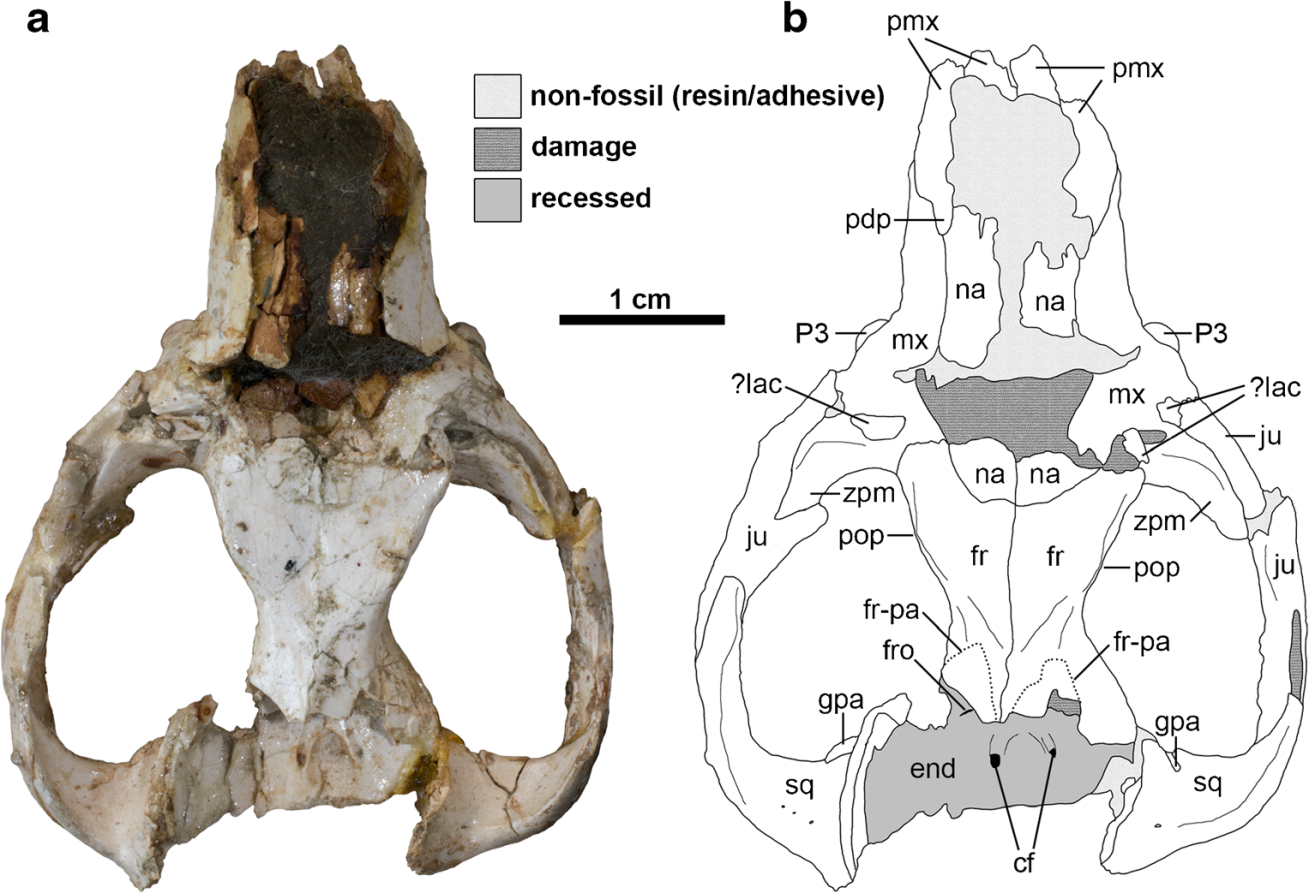
Cranium of *Epidolops ameghinoi* (DGM 321-M - holotype) in dorsal view. **a** photograph; **b** interpretative drawing. Abbreviations: cf = carotid foramen; end = endocranial cavity; fr = frontal; fr-pa = reconstructed path of frontal-parietal suture; fro = foramen rotundum; gpa = glenoid process of the alisphenoid; ju = jugal; ?lac = ?lacrimal; mx = maxilla; na = nasal; pdp = posterodosal process of the premaxilla; pmx = premaxilla = pop = postorbital process; sq = squamosal

**Fig. 2:**
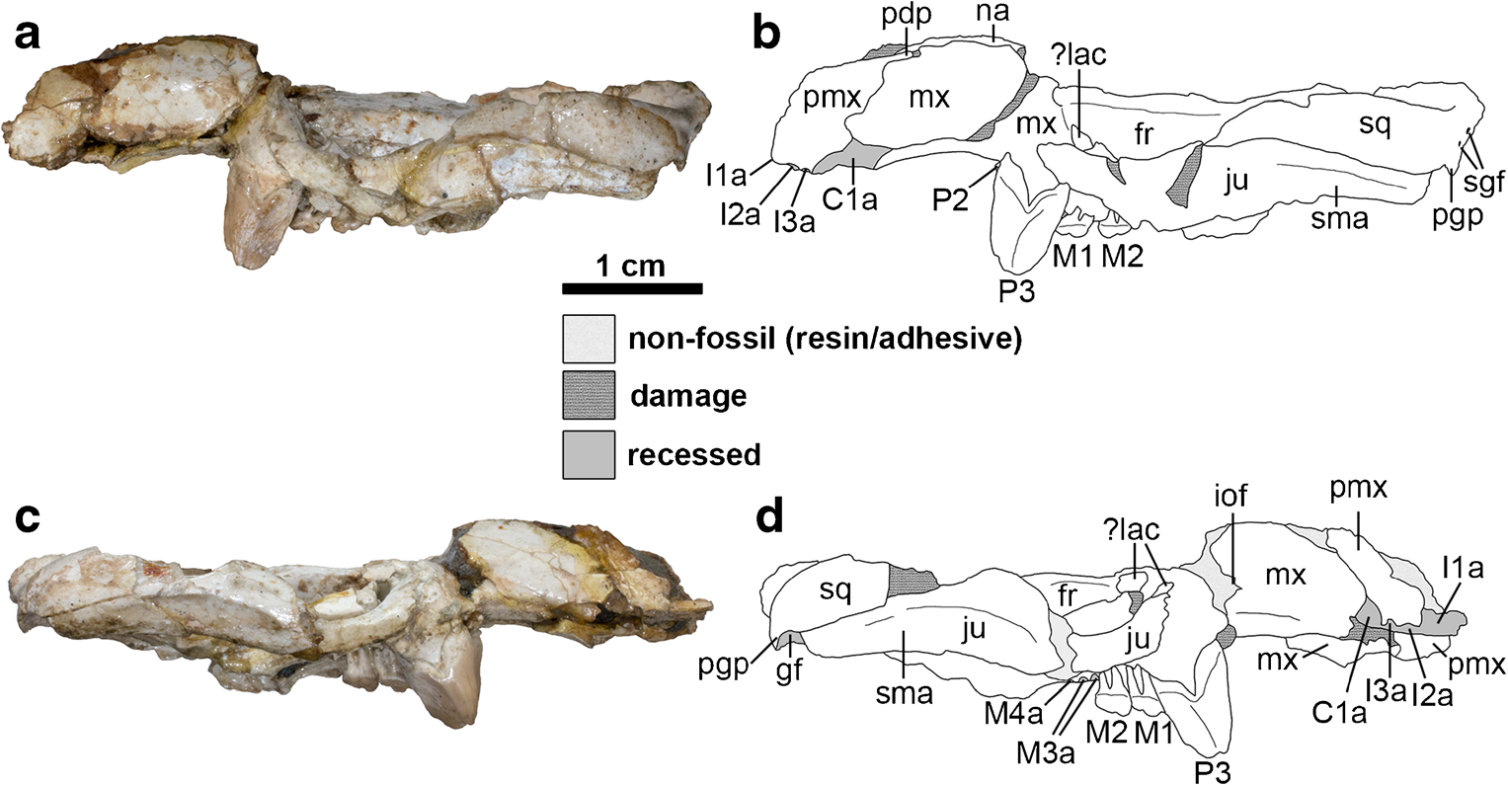
Cranium of *Epidolops ameghinoi* (DGM 321-M - holotype) in lateral view. **a** photograph of left lateral view; **b** interpretative drawing of left lateral view; **c** photograph of right lateral view; **d** interpretative drawing of right lateral view. Abbreviations: C1a = upper canine alveolus; fr = frontal; gf = glenoid fossa; I1a = first upper incisor alveolus; I2a = second upper incisor alveolus; I3a = third upper incisor alveolus; iof = position of infraorbital foramen; ju = jugal; ?lac = ?lacrimal; M1 = first upper molar; M2 = second upper molar; M3a = third upper molar alveoli; M4a = fourth upper molar alveolus; mx = maxilla; na = nasal; pdp = posterodosal process of the premaxilla; P2 = second upper premolar; P3 = third upper premolar; pgp = postglenoid process; pmx = premaxilla; sgf = supraglenoid foramina; sma = sulcus for masseter muscle; sq = squamosal

**Fig. 3:**
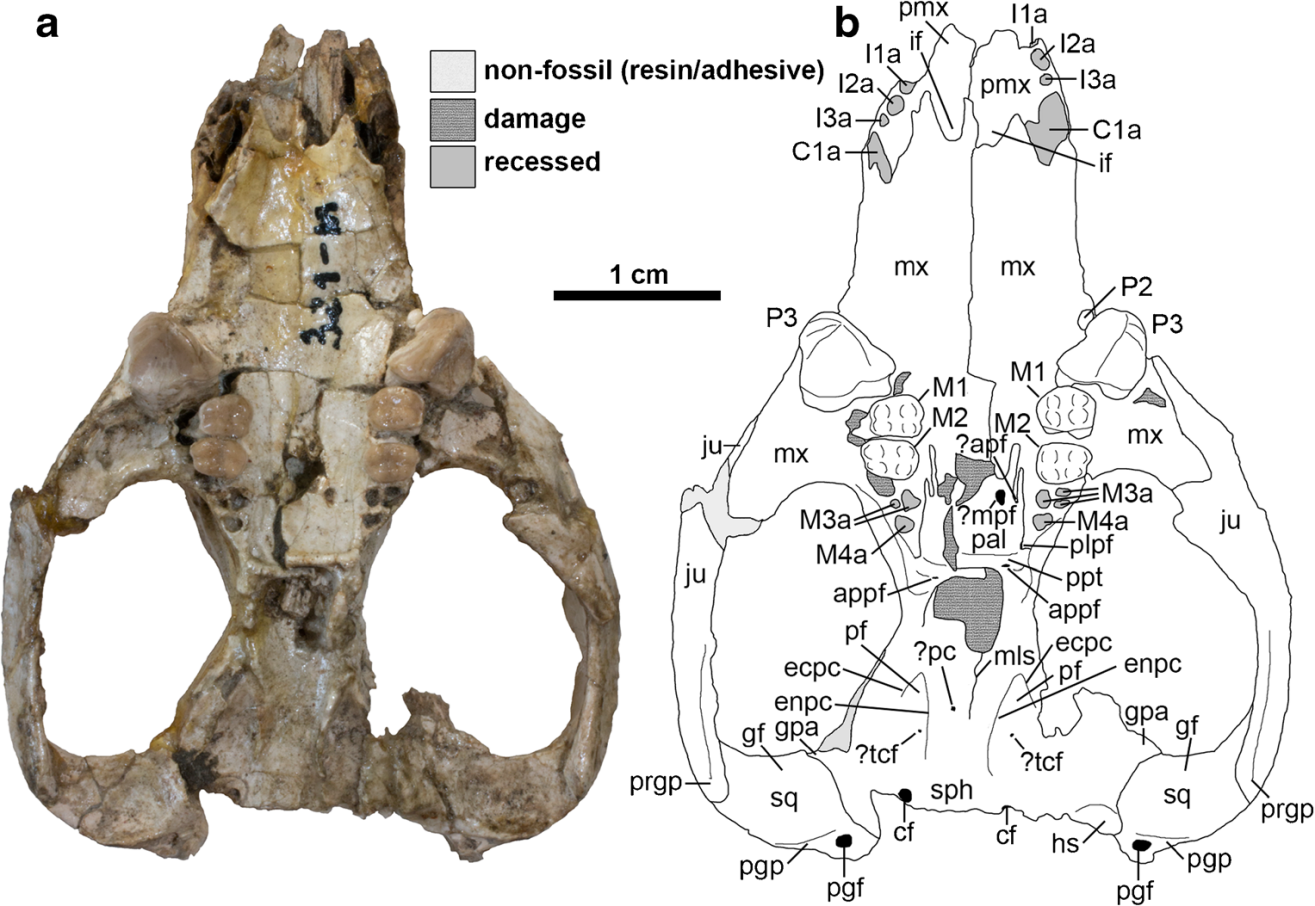
Cranium of *Epidolops ameghinoi* (DGM 321-M - holotype) in ventral view. **a** photograph; **b** interpretative drawing. Abbreviations: ?apf = ?accessory palatal foramen; appf = accessory posterolateral palatal foramen; C1a = upper canine alveolus; cf = carotid foramen; ecpc = ectopterygoid crest; enpc = entopterygoid crest; gf = glenoid fossa; gpa = glenoid process of the alisphenoid; hs = hypotympanic sinus; I1a = first upper incisor alveolus; I2a = second upper incisor alveolus; I3a = third upper incisor alveolus; if = incisive foramen; ju = jugal; M3a = third upper molar alveoli; M4a = fourth upper molar alveolus; ?mpf = ?major palatine foramen; mls = midline suture; mx = maxilla; pal = palatine; ?pc = ?pterygoid canal; pf = pterygoid fossa; pgf = postglenoid foramen; pgp = postglenoid process; plpf = posterolateral palatal foramen; pmx = premaxilla = ppt = postpalatine torus; prgp = preglenoid process; sph = sphenoid complex; sq = squamosal

DGM-321 M comprises a cranium plus associated left and right mandibles (Figs. 1-3, 5, and 6). A large fragment of what may be fossilized bone is present in the same box; it is labelled as 321-M, but it differs in color and texture from the other material, and its shape does not correspond to any missing region of the cranium of *E. ameghinoi* (it appears to be too large and too thick to be part of the posterior cranial roof). I am confident that this fragment does not pertain to *E. ameghinoi* - indeed, it is not unambiguously mammalian - and I do not discuss it further here.

The total preserved length of the cranium is approximately 51 mm, whilst maximum width across the zygomatic arches is approximately 37 mm. Total intact length of the cranium was probably approximately 55 mm. The cranium is crushed dorsoventrally, with the degree of crushing much greater posterior to the rostrum (Fig. 2). The dorsal surface of the rostrum is largely missing, with only fragments of the nasals remaining. The frontals are largely intact, but the parietals are missing.

The posterior part of the cranium (basioccipital, supraoccipital, paired exoccipitals, and paired parietals, plus the interparietal, if the last bone was present) is not preserved. The ventral floor of the anterior part of the braincase is visible in dorsal view (Fig. 1): the foramen rotundum (the exit of the maxillary branch of the trigeminal nerve; Fig. 1: fro) and the internal opening of the carotid foramen (Fig. 1: cf) are visible on both the left and right sides. Flynn and Wyss (2004) noted that loss of the posterior braincase (as also seen in cranial specimens of the polydolopimorphians *Kramadolops mckennai* and *Bonapartherium hinakusijum*; Pascual 1981; Flynn and Wyss 2004) resembles the damage produced by modern predatory birds (such as owls) when feeding on mammals.

The auditory region of DGM-321 M is slightly more complete on the left side (Figs. 3 and 5), but neither petrosal is preserved. The squamosal contribution to the sidewall of the braincase is largely missing. The zygomatic arches are largely complete but damaged, and the glenoid fossa and postglenoid process are complete on both sides. Foramina for the postglenoid venous system are preserved. The left and right mandibles are largely complete, but the left and right incisor arrays, right c1, and left and right m4 are missing or broken (Fig. 6).

### Nasal

Both nasals are badly damaged and fragmentary, with the left slightly more complete (Fig. 1: na). Anteriorly, fragments of left and right nasals are preserved in contact with the facial processes of the maxillae, where they form the roof of the nasal cavity. More posteriorly, crushing of the skull means that the nasals are poorly preserved, but the posterior contact with the frontals is intact. The posterior margins of the nasals form a gentle convex curve. The nasals terminate well posterior to the anterior margin of the orbit. It is unclear exactly how far the nasals extend laterally, but, based on the right side of the cranium, it seems likely that the maxilla and frontal (rather than the nasal and lacrimal) were in contact. Antorbital vacuities (a highly distinctive feature of living caenolestids; Osgood 1921, 1924; Patterson and Gallardo 1987; Voss and Jansa 2009: 29; Ojala-Barbour et al. 2013) appear to be absent.

### Premaxilla

The anterior and dorsal parts of the premaxillae are damaged, particularly on the right side (Figs. 1-3: pmx). In lateral view (Fig. 2), the suture with the maxilla can be identified, extending posterodorsally from level with the anterior margin of the canine alveolus. A distinct posterodorsal process (sensu Wible 2003) extends posteriorly to a point approximately level with the middle of the diastema between C1 and P2 (Figs. 1 and 2: pdp). The incisive foramina (Fig. 3: if) are crushed, obscuring their exact morphology, but they are short, with their posterior margins approximately level with the posterior half of the C1 alveolus. The posterior borders of the incisive foramina are formed by the maxillae.

The left and right premaxillae both preserve evidence of four closely-packed alveoli (Figs. 2 and 3). Posteriorly, the premaxilla forms the anterior margin of a large alveolus for a large, single-rooted C1, with the remainder formed by the maxilla (Figs. 2 and 3: C1a). Immediately anterior to this, three alveoli are present, which presumably housed three single-rooted incisors. The posteriormost alveolus (Fig. 3: I3a) is roughly circular, whereas the middle alveolus (Fig. 3: I2a) is somewhat rectangular, being slightly longer mesiodistally than labiolingually. Only the posterior part of the anteriormost alveolus (Fig. 3: I1a) is preserved, but the incisor it housed was probably the largest of the three. The anterior end of the premaxilla is not preserved, and hence the presence of one or two additional anterior incisors (assuming a maximum incisor count of five) cannot be entirely ruled out. However, in metatherians with four or five upper incisors, I2–4 are usually similar in size (pers. obs.), and thus the large size of the anteriormost alveolus suggests that no more than three incisors were present. On the (admittedly questionable) assumption that teeth are lost from the posterior end of the incisor array (Ziegler 1971), I tentatively identify them as I1–3. If only three incisors are present, then I1 must have been set back posteriorly somewhat from the anterior end of the premaxilla. The I1 alveolus appears relatively shallow, and hence this tooth is unlikely to have been open-rooted.

### Maxilla

**Fig. 4:**
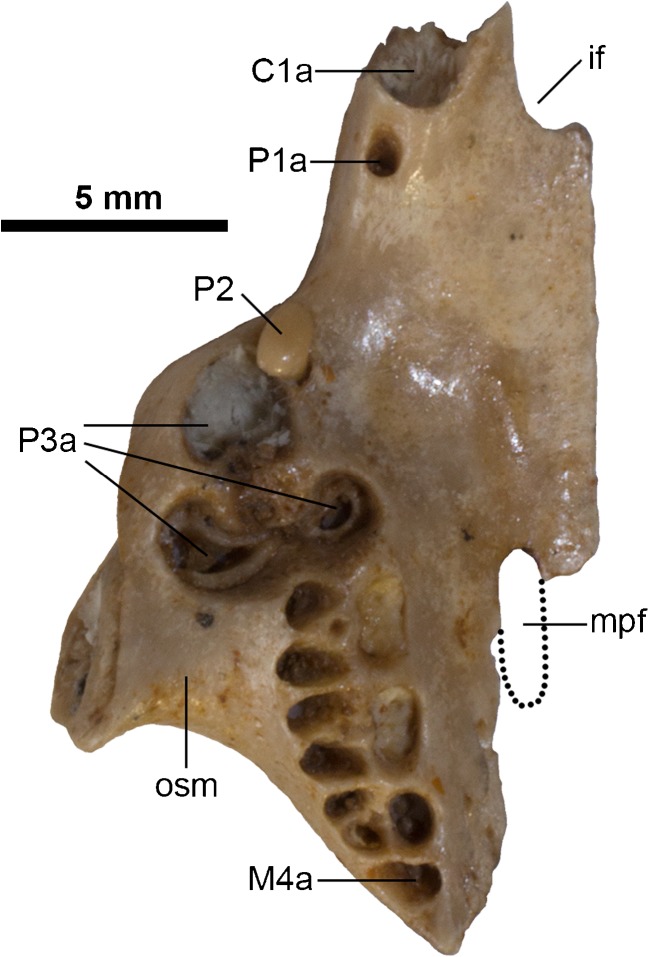
Isolated partial right maxilla of *Epidolops ameghinoi* (DGM 898-M) in ventral view, with inferred extent of maxillopalatine fenestra indicated. Abbreviations: C1a = upper canine alveolus; if = incisive foramen; M4a = upper fourth molar alveolus; mpf = maxillopalatine fenestra; osm = area of origin of superficial masseter; P1a = upper first premolar alveolus; P3a = upper third premolar alveoli

The exact position of the infraborbital foramen cannot be determined in DGM 321-M, but its rough location can be inferred on the right side (Fig. 2c and d: iof), directly above P2 and well anterior of the suture with the jugal. This interpretation is confirmed by some isolated maxillary fragments that preserve the infraorbital foramen in this position (e.g., DGM 198-M, 204-M, 913-M). In other maxillary specimens, the infraorbital foramen is level with the anterior margin of P3 (e.g., DGM 201-M, 205-M). The suture with the jugal is essentially straight, but a slight saw-edge is visible on the right side of DGM 321-M (Fig. 2c and d). There is no antorbital fossa. The maxilla does not form a distinct masseteric process at the base of the zygomatic arch in DGM 321-M (Figs. 2 and 3). Other specimens of *E. ameghinoi* preserve a very weakly raised area on the maxilla corresponding to the likely area of origin of the superficial masseter (e.g., DGM 898-M; Fig. 4: osm), but in none of these can this structure be reasonably described as forming a distinct masseteric process.

In dorsal view (Fig. 1), the anterior root of the zygomatic arch is anteroposteriorly elongate (as noted by Flynn and Wyss 2004: 88); this is somewhat exaggerated in DGM 321-M due to dorsoventral crushing, but isolated maxillary fragments (e.g., DGM 898-M; Fig. 4) show that this morphology is not entirely artefactual. Crushing means that the exact contribution of the maxilla to the orbital fossa, and its relationships to the other bones in this region, is unclear in DGM 321-M. However, a robust zygomatic process of the maxilla extends posterolaterally on the inside of the zygomatic arch of the jugal (Fig. 1: zpm). Isolated maxillae (DGM 205-M and 898-M) show that the maxillary foramen was completed dorsally by the lacrimal, rather than being entirely enclosed by the maxilla. DGM 898-M also indicates that the exposure of the maxilla within the orbital fossa was relatively small, and hence the palatine and lacrimal were probably in contact.

In ventral view (Fig. 3), the maxilla forms the majority of the palate, from its anterior contact with the premaxilla (where it forms the posterior borders of the incisive foramina, level with C1) posteriorly, with the palatine forming the posteromedial section. The palate between P3-M4 is damaged in its midline in DGM 321-M, with the right maxilla broken at the labial roots of M1–2 and its palatal process displaced dorsomedially (contra Paula Couto 1952c: fig. 2). As a result, it is difficult to determine whether palatal vacuities are present or absent in DGM 321-M. However, DGM 898-M (Fig. 4) and MNRJ 2879-V (both isolated maxillae) preserve the anterior margin of a small palatal vacuity, which extends anteriorly to approximately level with the bony septum between the P3 and M1 alveoli. Further posteriorly, the path of the maxillopalatine suture can be traced in DGM 898-M (Fig. 4), suggesting that the vacuity was enclosed posteriorly by the palatine and hence that it is a maxillopalatine fenestra sensu Voss and Jansa (2009).

The exact size of the intact maxillopalatine fenestrae in *E. ameghinoi* cannot be determined, but they appear to have been very short anteroposteriorly, probably only extending posteriorly as far as M2 (Fig. 4: mpf). The palatal suture between the maxilla and the palatine is somewhat difficult to identify in DGM 321-M due to the presence of obscuring adhesive, but it appears to be complex and interdigitating medial to M2–3 (Fig. 3; see also Paula Couto 1952c: fig. 2: left [anatomical right] side). The maxilla forms the lateral border of the posterolateral palatal foramen sensu Voss and Jansa (2009 = minor palatine foramen sensu Wible 2003), with the maxillopalatine suture passing through this foramen (Fig. 3: plpf).

The maxilla preserves alveoli for at least seven teeth (Figs. 2 and 3). At its anterior end, it clearly formed the majority of the alveolus for the large, single-rooted C1 (Figs. 2 and 3: C1a); the premaxilla seems to have formed the anterior margin of the C1 alveolus in DGM 321-M (Figs. 2 and 3: C1a) and also in DGM 898-M (Fig. 4: C1a) and 917-M, but this alveolus is entirely within the maxilla in MNRJ 2879-V (polymorphism in this feature occurs in a few living marsupials, namely the caenolestid *Lestoros inca* and several peramelemorphians; pers. obs.). The C1 is unknown in *E. ameghinoi*. However, based on the size and position of its alveolus, it was probably similar in morphology to the C1 of *Bonapartherium hinakusijum* (see Pascual 1981: figs. 1-3), which is large and possibly also somewhat procumbent (if this is not an artefact of the dorsoventral crushing of the best preserved *B. hinakusijum* cranium, MMP 1408).

There is a large diastema separating C1 from P2; this region is damaged on both left and right sides of DGM 321-M (Figs. 2 and 3), and so the presence of P1 cannot be ruled out. In fact, DGM 898-M (Fig. 4: P1a), 917-M, and MNRJ 2879-V all indicate the presence of a very small, single-rooted P1 ~ 1 mm behind the posterior margin of the C1 alveolus. DGM 917-M preserves the root and base of the crown of P1, demonstrating that this tooth was slightly procumbent. Marshall (1982a) stated that P1 was sometimes absent in *E. ameghinoi*, but all three specimens in which the region of the maxilla immediately posterior to C1 is well preserved (DGM 898-M, 917-M, and MNRJ 2879-V) have a P1. Marshall (1982a) also reported that P1 is double-rooted in MNRJ 2879-V, but I interpret the posterior “alveolus” in this specimen as an artefact due to damage.

P2 is a very small, button-like tooth located at the base of the enormous, plagiaulacoid P3. It is single-rooted in DGM 321-M (Fig. 3: P2) and in several other specimens (DGM 912-M, 918-M), but double-rooted (MNRJ 2879-V; DGM 898-M – Fig. 4: P2) or incipiently double-rooted (DGM 917-M) in others. The relative sizes and arrangement of P2 and P3 in *E. ameghinoi* are strongly reminiscent of the condition seen in the living Australian diprotodontian *Burramys parvus* (the mountain pygmy possum; see Ride 1956). DGM 898-M reveals the root morphology of P3 (Fig. 4: P3a): the anterior alveolus is single, whereas the posterior alveolus is incipiently divided and so has a mediolaterally-oriented figure-of-8 shape in dorsal view; however, the roots within the posterior alveolus are fully divided in DGM 898-M, i.e., there are two posterior roots, one posterolabial and one posterolingual. The maxilla flares distinctly laterally where it houses the P3, and hence the skull broadens markedly at this point (Figs. 1, 3 and 4); this is in contrast to the rostrum, which is relatively constant in width (Figs. 1 and 3). Posteromedial to P3, M1–3 are each housed in three alveoli: two small roots on the labial side, and a single, broader root on the lingual half (Figs. 3 and 4). An alveolus for a small, single-rooted M4 is present posterior to the lingual root of M3 (Figs 3 and 4: M4a). The molar row is oriented roughly anteroposteriorly, but is positioned distinctly medial to P3: the labial roots of M1 are posterior to lingual root of P3 (Figs. 3 and 4).

In dorsal view, the exact relationship between the maxilla and the other bones forming the roof of the anterior region of the cranium is unclear due to crushing and displacement (Fig. 1); however, based on the right side, it seems likely that the maxilla and frontal were in contact (as in most marsupials), rather than the nasal and lacrimal.

### Lacrimal

A few fragments of lacrimal may be preserved on both sides of DGM 321-M (Figs. 1 and 2: ?lac). These possible remnants do not give any indication of (for example) the presence or absence of a distinct orbital crest, or the number and arrangement of the lacrimal foramina. However, the facial exposure of the lacrimal appears to have been relatively small, and the right side of DGM 321-M suggests that the maxilla and frontal were in contact, rather than the nasal and lacrimal (Fig. 1).

The arrangement of bones in the orbital mosaic is also unclear in DGM 321-M. However, isolated maxillae (DGM 205-M, 898-M) indicate that the maxillary foramen was completed dorsally by the lacrimal, and suggest that the lacrimal and palatine were probably in contact.

### Palatine

In ventral view (Fig. 3), the palatine forms the posteromedial section of the hard palate, contacting the maxilla along a complex, interdigitating suture (see also Paula Couto 1952c: fig. 2). Large palatine fenestrae sensu Voss and Jansa (2009) are absent, but a distinct foramen is visible within the palatal process of the palatine (most obviously on the left side), medial to M3 (Fig. 3: ?mpf). A much smaller foramen appears to be present lateral to this, close to or within the suture with the maxilla Fig. 3: ?apf). The larger foramen is plausibly the major palatine foramen for the major palatine artery, vein, and nerve (if these did not pass through the maxillopalatine fenestrae, which are located further anteriorly). The smaller foramen may be an accessory palatine foramen, which transmits branches of the accessory palatine nerve and artery (Wible and Rougier 2000; Wible 2003). The palatine forms a distinct, raised postpalatal torus (Fig. 3: ppt), and also forms the posterior and medial borders of the posterolateral palatal foramen (which is completed anteriorly and laterally by the maxilla; Fig. 3: plpf). An accessory posterolateral palatal foramen is also identifiable (Fig. 3: appf), extending anteroposteriorly through the postpalatine torus, posteromedial to the posterolateral palatal foramen. The palate lateral to the posterolateral palatal foramen does not form distinct “corners,” unlike the condition in most didelphids (Voss and Jansa 2009, 2003).

**Fig. 5:**
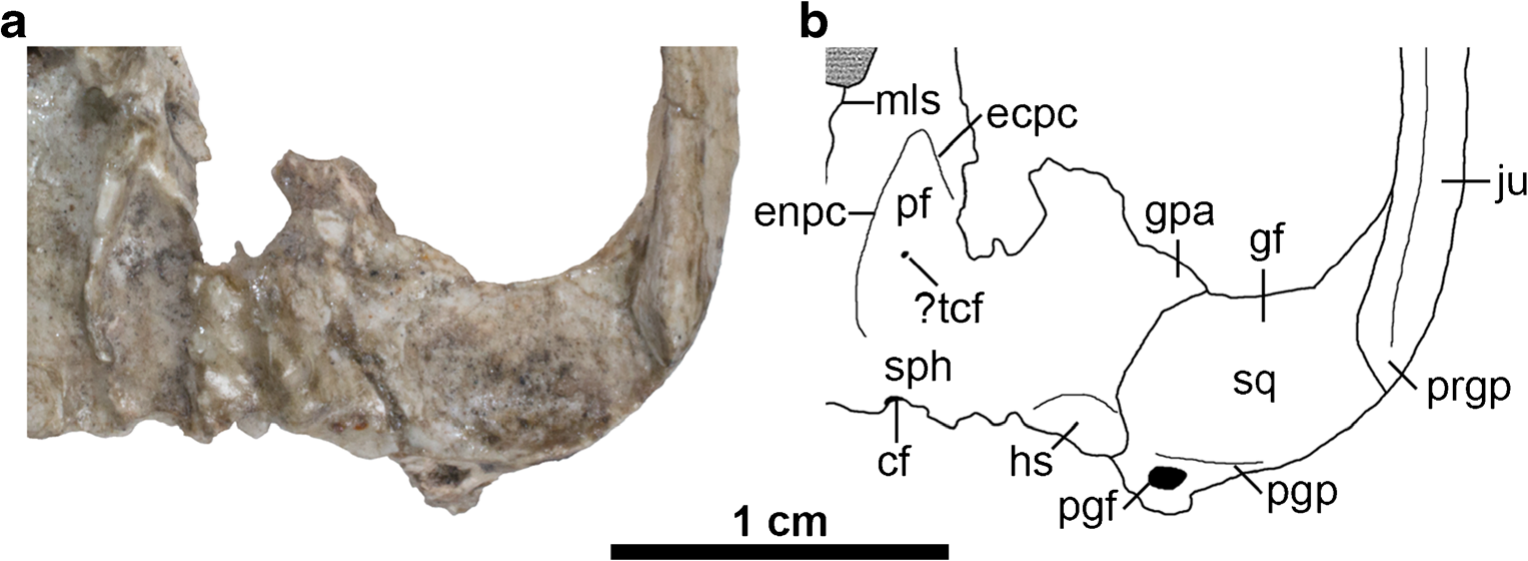
Left basicranial region of *Epidolops ameghinoi* (DGM 321-M - holotype) in ventral view. **a** photograph; **b** interpretative drawing. cf = carotid foramen; ecpc = ectopterygoid crest; enpc = entopterygoid crest; gf = glenoid fossa; gpa = glenoid process of the alisphenoid; hs = hypotympanic sinus; ju = jugal; mls = midline suture; pf = pterygoid fossa; pgf = postglenoid foramen; pgp = postglenoid process; prgp = preglenoid process; sph = sphenoid complex; sq = squamosal

Posterior to the postpalatine torus, the palatines contribute to the lateral walls of the nasopharyngeal region. The anterior part of this region is badly damaged, but there is a faint midline suture more posteriorly (Figs. 3 and 5: mls); this probably represents midline contact between either the palatines or the pterygoids, but damage and obscuring glue mean that these alternatives cannot be distinguished. Ride (1956) proposed that midline contact between the palatines seen in diprotodontians with very large plagiaulacoid P3s is an adaptation to strengthen the palatal region of the cranium; if so, this may explain its possible presence in *E. ameghinoi*, which has a similar P3 morphology.

In lateral view, crushing and general damage mean that the exact contribution of the palatine to the orbital mosaic is unclear, as is the location and morphology of the sphenopalatine foramen. Based on the left side, it seems likely that the palatine prevented contact between the maxilla and alisphenoid.

### Jugal

Isolated maxillary specimens demonstrate that the jugal did not contribute to the slightly raised area for origin of the superficial masseter (Figs. 2, 3 and 4: osm; see above). The jugal is deep dorsoventrally, and together with the squamosal forms a robust zygomatic arch (Figs. 1-3).

The concave dorsal margin of the jugal suggests that the orbit was relatively large (Fig. 2). The anterior part of the jugal is buttressed medially by a prominent zygomatic process of the maxilla (Fig. 1: zpm). More posteriorly, there does not appear to be a distinct frontal process marking the attachment of the postorbital ligament on the dorsal margin of the jugal (Fig. 2). The jugal extends under the squamosal as far as the glenoid fossa, terminating in a ventrally deep but mediolaterally narrow preglenoid process (Fig. 3: prgp); this process is better preserved on the left side of DGM 321-M than on the right. The posterior end of the preglenoid process terminates in a distinct facet that is oriented posterolateral to anteromedial. The lateral face of the zygomatic process of the jugal is marked by a prominent ventral sulcus for the masseter muscles (Fig. 2: sma**)**, while the medial face is strongly concave.

### Frontal

In dorsal view, the postorbital process forms a gently-rounded lateral protuberance (Fig. 1: pop). A relatively sharp postorbital constriction is present immediately posterior to the postorbital process. A faint temporal line can be traced posteromedially back from the postorbital process, reaching the midline ~3.5 mm anterior from the posterior edge of the frontals. The median frontal suture is unfused. Either side of the posterior end of the median suture, areas of the frontal that were overlapped by the parietal when the skull was intact are identifiable: the suture between the paired frontals and parietals was evidently W-shaped in dorsal view, with the base of the W oriented anteriorly (Fig. 1: fr-pa). The posterior end of the median frontal suture is slightly raised, suggesting that a sagittal crest may have been present on the parietals.

### Squamosal

Parts of both the left and right squamosal are preserved in DGM 321-M (Figs. 1-3 and 5: sq), with the glenoid region largely intact on both sides (Figs. 3 and 5). However, only part of the squamosal contribution to the lateral braincase is preserved, and the region posterior to the postglenoid process (including the part surrounding the external auditory meatus) is missing. In ventral view, the glenoid fossa (Figs. 3 and 5: gf) is mediolaterally broad and gently concave, forming a smoothly curved surface that extends posteroventrally onto the anterior face of the postglenoid process (Figs. 2, 3 and 5: pgp). There is no raised articular eminence anteriorly, whereas this structure is found in most diprotodontians (Aplin 1987, 1990). The postglenoid process is broad mediolaterally and low, with its anterior face slightly concave and its posterior face slightly convex. The posteromedial edge of the postglenoid process is grooved for the passage of the postglenoid vein. The postglenoid foramen itself (Figs. 3 and 5: pgf) is located slightly more dorsal, namely medial and slightly posterior to the postglenoid process. Although the region is damaged on both sides of DGM 321-M, the slightly better preserved left side suggests that the postglenoid foramen was probably fully enclosed by squamosal. Two supraglenoid foramina, visible on the left side of DGM 321-M dorsal and slightly posterior to the postglenoid process in lateral view (Fig. 2a and b: sgf), appear to be continuous with the postglenoid foramen (confirmed by breakage on the right side).

The contact between the squamosal and alisphenoid is not obvious. On the right side, the part of the alisphenoid that contacted the squamosal appears to have flaked away, but the suture can still be traced, coursing posteromedially from the anteromedial corner of the glenoid fossa. This morphology is confirmed on the left side, in which the alisphenoid is more intact, and which indicates that a distinct glenoid process of the alisphenoid (= the entoglenoid process of the alisphenoid sensu Muizon 1998, 1999; Figs 1, 3 and 5: gpa) was present, extending along the anterior margin of the medial part of the glenoid fossa. Based on the left side of DGM 321-M, it is unlikely that the squamosal contributed to the roof of the hypotympanic sinus.

In lateral view, the zygomatic process of the squamosal is deep, and together with the underlapping jugal, forms a robust zygomatic arch (Fig. 2).

In dorsal view (Fig. 1), the zygomatic process of the squamosal forms a prominent ridge on its dorsal margin; posteriorly, where this ridge merges with the squamosal contribution to the braincase, it forms the posterior and lateral wall of a distinct, roughly triangular depression. This depression provides attachment for the temporalis, and a number of small foramina are visible within it.

### Pterygoid

Grooves that presumably housed the pterygoid are visible in the sphenoid complex on the right side of DGM 321-M, but the right pterygoid itself appears to be largely or entirely absent; based on the disposition of these grooves, it is unlikely that the pterygoid extended posteriorly as far as the external opening of the carotid foramen (Figs. 3 and 5: cf). The pterygoid appears to be at least partially preserved on the left side, where it contributes to the entopterygoid crest (Figs. 3 and 5: enpc). The pterygoid is damaged, but the preserved part does not extend posteriorly as far as the carotid foramen. The precise extent of the pterygoids when intact cannot be unambiguously inferred in DGM 321-M, and so it is uncertain whether the midline suture visible in the nasopharyngeal region (Figs. 3 and 5: mls) represents midline contact by the palatines or by the pterygoids (see above).

### Sphenoid Complex

The sphenoid complex (Figs. 3 and 5: sph) comprises the presphenoid, basisphenoid, and paired orbitosphenoids and alisphenoids (Wible 2003). Distinct sutures between these bones are not identifiable in DGM 321-M, and so this region will be described as a whole. In lateral view, neither the sphenorbital fissure nor the foramen rotundum (both of which open within the sphenoid complex) are identifiable with any certainty. On the left side, there is a piece of bone that is in contact with the palatine anteriorly and the frontal dorsally; this is presumably part of the alisphenoid. However, damage to the region posterior to this means that the full extent of the alisphenoid (and whether it was in contact with the parietal, or whether instead the frontal and squamosal were in contact) is unclear, as it is on the right side.

In ventral view, the sphenoid complex preserves some particularly significant features (Figs. 3 and 5). Posteriorly, the ventral part of the back of the cranium appears to have broken away along the basisphenoid-basioccipital suture. Prominent entopterygoid crests extend posteriorly (Figs. 3 and 5: enpc); on the left side, the pterygoid also contributes to this crest, but this bone is not preserved on the right. Lateral to these crests are well-excavated pterygoid fossae (Figs. 3 and 5: pf), and on the left side there is also evidence for an ectopterygoid crest (Figs. 3 and 5: ecpc) that encloses at least the anterior half of the pterygoid fossa laterally, indicating that the pterygoid musculature of *E. ameghinoi* was well developed. A tiny foramen appears to be present within this fossa on both sides of DGM 321-M (Figs. 3 and 5: ?tcf); it may be the transverse canal foramen, but CT data will be required to confirm this. The external opening of the carotid foramen is visible at the posterior margin of the sphenoid complex on both sides of DGM 321-M (Figs. 3 and 5: cf). It is located slightly further anterior on the right side compared to the left, but slight bilateral asymmetry in the position of this foramen is not uncommon among marsupials (pers. obv.); alternatively, this might be a taphonomic artefact.

A glenoid process of the alisphenoid (Figs. 3 and 5: gpa) extends along the anterior margin of the medial part of the glenoid fossa, and more posteriorly the suture between the alisphenoid and squamosal extends in a posteromedial direction. Medial to the posterior part of this suture, the part of the alisphenoid forming the hypotympanic sinus appears to be preserved on the left side of DGM 321-M, sloping dorsally where it starts to form the anterolateral part of the roof of this sinus (Figs. 3 and 5: hs). This preserved part of the hypotympanic sinus roof is not strongly excavated.

Apart from a slight rise medially, there is no evidence of an alisphenoid tympanic process along the anterior border of the putative hypotympanic sinus (Figs. 3 and 5: hs). This apparent absence may be an artefact due to the obscuring adhesive; however, there is no sign of the broken base of an alisphenoid tympanic process as is clearly visible in, for example, fossil crania of the Australian marsupialiform *Yalkaparidon coheni* (see Beck et al. 2014: figs. 2 and 8) or the peramelemorphian *Yarala burchfieldi* (see Muirhead 2000: figs. 1 and 3). There is also no tympanic process of the squamosal.

It is possible that another bone formed an ossified floor for the anterior part of the hypotympanic sinus, but that this bone is not preserved in DGM 321-M. For example, it is possible that the petrosal enclosed the hypotympanic sinus in *E. ameghinoi*, as it does in acrobatid diprotodontians (Aplin 1987, 1990). However, multiple isolated marsupialiform petrosals are known from Itaboraí (including the Type II petrosals of Ladevèze 2004, which are plausibly referable to *E. ameghinoi* – see below), and none preserve evidence of extensive tympanic processes that could enclose the hypotympanic sinus (Ladevèze 2004, 2007; Ladevèze and Muizon 2010). Furthermore, the entire auditory region of acrobatids is highly autapomorphic (Aplin 1987, 1990), whereas the preserved morphology of this region in DGM 321-M appears relatively plesiomorphic within Marsupialiformes (see below). Alternatively, the hypotympanic sinus could have been floored by one or more entotympanics; however, entotympanics do not occur in any known metatherian (with the possible exceptions of acrobatids and some specimens of *Phalanger orientalis*; Maier 1989; Aplin 1990; Norris 1993; Sánchez-Villagra 1998), and it seems unlikely that *E. ameghinoi* was an exception to this general rule. Instead, based on available evidence, I conclude that *E. ameghinoi* probably lacked an ossified hypotympanic sinus floor. A notch medial to the slightly raised area at the anteromedial corner of this region may represent the anterior margin of the foramen ovale.

In dorsal view, the sphenoid contribution to the endocranium is visible; the internal openings of the carotid foramina (Figs. 3 and 5: cf) can be seen either side of the hypophyseal fossa, and the left foramen rotundum (Figs. 3 and 5: fro) is also identifiable. Flynn and Wyss (2004: 89) observed that the braincase of *E. ameghinoi* appears to be proportionately much smaller than that of *Kramadolops mckennai*, but it seems similar to *Bonapartherium hinakusijum* in this regard (see Pascual 1981: figs. 1-3; Goin et al. 2016: fig. 5.10).

### Mandible

**Fig. 6:**
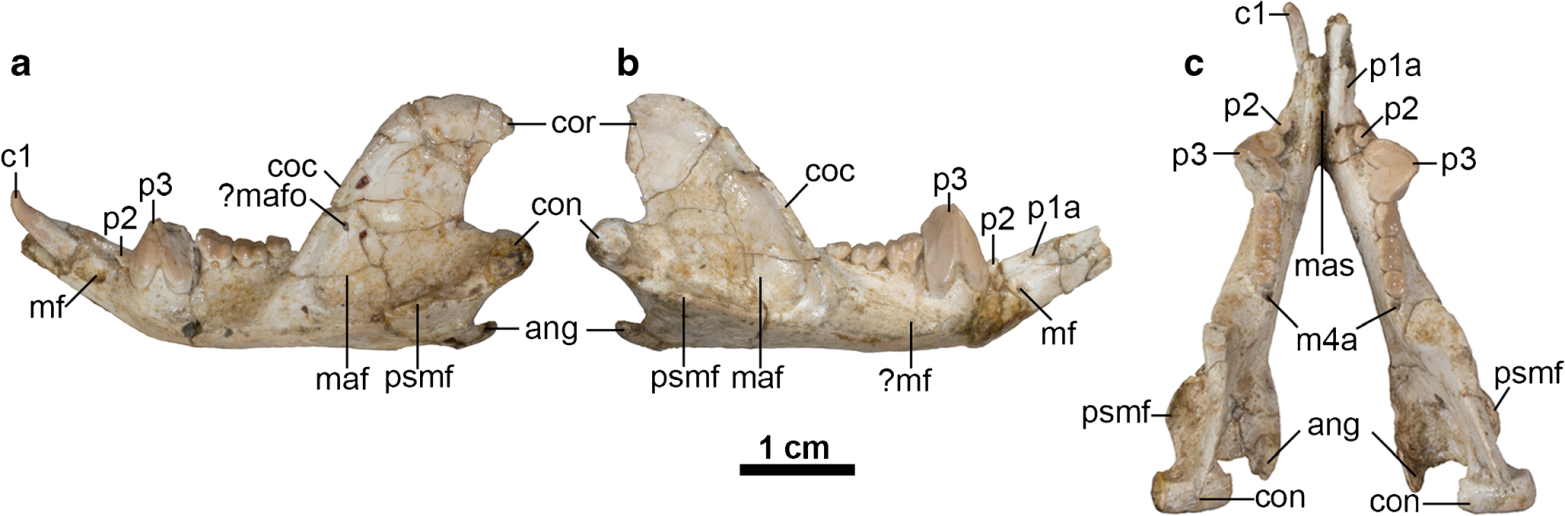
Left and right mandibles of *Epidolops ameghinoi* (DGM 321-M - holotype). **a** left mandible in lateral view; **b** right mandible in lateral view; **c** a left and right mandibles in dorsal view. Abbreviations: ang = angular process; c1 = lower canine; coc = coronoid crest; con = mandibular condyle; cor = coronoid process; m4a = fourth lower molar alveolus; maf = massteric foramen; mas = mandibular symphysis; mf = mental foramen; ?mafo = ?massteric foramen; ?mf = ?mental foramen; p1a = first lower premolar alveolus; p2 = second lower premolar; p3 = third lower premolar; psmf = posterior shelf of the massteric fossa

The left and right mandibles of DGM 321-M are preserved largely intact (Fig. 6). They are joined by adhesive at the symphysis (Fig. 6c: mas), but the symphysis is nevertheless clearly unfused. Multiple additional dentaries of *E. ameghinoi* are present in the DGM and MNRJ collections, and they have been used to supplement the description here.

The mandibular ramus appears short and robust, and its lateral wall bulges out below p3, due to the enlarged roots of this tooth. Anterior to this bulge, the dorsal and ventral margins of the ramus slope distinctly dorsally. A large mental foramen (Fig. 6a and b: mf) is present anteroventral to the small p2, level with the exposed roots of p3. A distinct sulcus on the dorsal surface of the mandibular ramus extends anteriorly from medial to the anterior root of p3. The symphysis (Fig. 6c: mas) appears to have extended posteriorly as far as the vestigial p2; in some other specimens the symphysis is posteriorly more extensive, reaching as far back as p3 in DGM 903-M and 904-M. Posterior to the bulge formed by p3, the lateral face of the mandibular ramus is slightly concave. A second, smaller mental foramen (Fig. 6b: ?mf) appears to be present below the midpoint of m1 on the right mandible, but this region is damaged on the left. Two mental foramina are present in several other specimens, e.g., DGM 903-M and DGM 904-N; the position of these foramina varies slightly between specimens, but the larger anterior foramen is typically ventral or anteroventral to p2, and the smaller posterior foramen is typically ventral or anteroventral to m1.

The coronoid process (Fig. 6a and b: cor) is tall, its anterior margin rising at an angle of ~55° relative to the horizontal, lateral to the m4. There is no large foramen (the “retromolar canal” sensu Hoffstetter and Villarroel 1974) within the retromolar space behind m4, whereas such a canal is present in argyrolagids (Simpson 1970b; Hoffstetter and Villarroel 1974; Sánchez-Villagra et al. 2000; Voss and Jansa 2009: 46; Babot and García-López 2016). A distinct foramen is also present in the retromolar space of at least some caenolestids (Simpson 1970b; Voss and Jansa 2009: 46), but Babot and García-López (2016) argued that this structure is not homologous with the retromolar canal of argyrolagids (see “Affinities of Argyrolagoids” below).

On the lateral face of the anterior margin of the coronoid process, a thick coronoid crest (Fig. 6a and b: coc) is present, continuing ventrally into the body of the mandibular ramus and defining the anterior limit of the well-excavated masseteric fossa (Fig. 6a and b: maf). In the midsection of the coronoid process, a shallow sulcus is visible on the anterior face of the coronoid crest. The posterior margin of the coronoid process is gently concave and slightly more vertical than its anterior margin. The coronoid process forms a blunt hook at its posterodorsal extremity. The condylar process (Fig. 6: con) is mediolaterally broad and roughly cylindrical. Posteroventrally, the angular process (Fig. 6: ang) is strongly medially inflected, forming a prominent medial platform, the posteromedial edge of which forms a posterodorsally-oriented hook. The mandibular foramen is easily identifiable on the medial face of the right mandible of DGM 321-M, but this region is damaged on the left.

Laterally, multiple foramina (Fig. 6a: ?mafo) are present within the masseteric fossa of at least some specimens (see Abbie 1939). In DGM 903-M, the largest of these foramina is on the anterior wall of masseteric fossa, and is concealed by the coronoid crest in lateral view.

The ventral margin of the masseteric fossa is formed by a prominent masseteric line; as this line extends posteriorly, it becomes crestlike and particularly extensive laterally, forming a distinct posterior shelf of the masseteric fossa (Fig. 6: psmf; Marshall and Muizon 1995; Wible 2003).

### Lower Dental Formula

The lower dentition of *E. ameghinoi* will be discussed starting with the molars and then moving anteriorly, because it is the antemolar formula that warrants the most detailed discussion. DGM 321-M and other mandibular specimens preserve evidence of four molars (Fig. 6c), with m1–3 doubled-rooted and m4 single-rooted (Fig. 6c: m4a). The molars show a clear decreasing gradient in size moving from m2 to 4, whereas m1 and m2 are similar in size. The double-rooted p3 is by far the largest tooth (Fig. 6: p3), and is preceded by a vestigial, single-rooted p2 (Fig. 6: p2). Anterior to this is a prominent diastema, ~4.5 mm long, between the p2 and the first procumbent tooth, which I interpret here as c1 (Fig. 6a and c: c1; see below), as did Marshall (1982a). On the right side of DGM 321-M, there appears to be a very small alveolus (Fig. 6b and c: p1a), even smaller than that of p2, ~3 mm anterior to p2 and ~1.5 mm posterior to c1; this alveolus seems to be absent from the left side of DGM 321-M. A groove extends anteriorly from this alveolus, suggesting that, if it did house a tooth, then it must have been small, single-rooted, and distinctly procumbent. Other mandibular specimens of *E. ameghinoi* preserve this alveolus (e.g., DGM 899-M 901-M, 903-M, 908-M), suggesting that it was normally present. A root is preserved within the alveolus in DGM 901-M, confirming that a procumbent tooth was indeed present. Although the crown of this tooth is not preserved in any *E. ameghinoi* specimen, it was probably very similar in morphology to the similarly-positioned tooth in the polydolopid *Kramadolops abanicoi* that Flynn and Wyss (1999: fig. 1) identified as p1, but which Goin et al. (2010: 86) referred to as c1 and Chornogubsky (2010) identified as ?c1. I concur with Flynn and Wyss (1999) that this tooth is p1 in *K. abanicoi* and also *E. ameghinoi* (see “Comparisons with Other Taxa Currently Included in Polydolopimorphia” below).

More anteriorly, there is a large, anteriorly-facing alveolus for an elongate, procumbent tooth. This tooth is preserved on the left side of DGM 321-M (Fig. 6a and c: c1); it is characterized by an elongate dentine root, and enamel is restricted to the distinctly-hooked tip. The enamel extends slightly further down the root labially than lingually, but it still extends only approximately 3 mm on the labial side and approximately 2.7 mm on the lingual side, compared with a total tooth length of approximately 7 mm. The elongate, dentine root and small enamelled tip of this tooth is strongly reminiscent of the canine morphology seen in older individuals of many marsupial taxa (e.g., didelphids, peramelemorphians, and dasyuromorphians), in which the canine root is extruded continuously throughout life and, as a result, the enamel becomes increasingly restricted to the tip of the tooth (Jones 1997: 2572; Jones and Stoddart 1998: 240; Voss and Jansa 2009: 48; Aplin et al. 2010: 15). By contrast, in marsupials that have an enlarged, procumbent anterior tooth in their lower dentition that can be unambiguously identified as an incisor (diprotodontians, paucituberculatans), the enamel extends far down the root. When the dentaries of DGM 321-M are placed in approximate articulation with the cranium, the procumbent lower tooth of the left dentary could plausibly occlude with C1 (which is missing), but its tip is distinctly posterior to the upper incisor alveoli. This evidence, together with the morphology of the more anterior teeth (discussed below), strongly suggests that the large procumbent lower tooth is c1 in *E. ameghinoi*, as also concluded by Paula Couto (1952c) and Marshall (1982a).

Paula Couto (1952c: figs. 3, 5A, 6A, 7A) illustrated the presence of two procumbent incisors anterior to the alveolus for c1 on the right side of DGM 321-M. These teeth appear to have broken off some time in the following 30 years, because they appear to be absent in Fig. 63a–b of Marshall (1982a); they are currently not in the box containing DGM 321-M and are presumably now lost. Paula Couto (1952c: figs. 3, 5A, 6A, 7A) indicated that these two incisors were arranged mediolaterally, with the more medial tooth slightly longer.

Examining DGM 321-M today, the roots of both of these incisors can be identified, with more of the root of the lateral tooth preserved; intriguingly, however, there appears to be evidence of at least one additional alveolus, dorsal to the roots of the two procumbent teeth. This raises the possibility that *E. ameghinoi* has three lower incisors, an interpretation that receives further support from examination of other isolated *E. ameghinoi* dentaries.

**Fig. 7:**
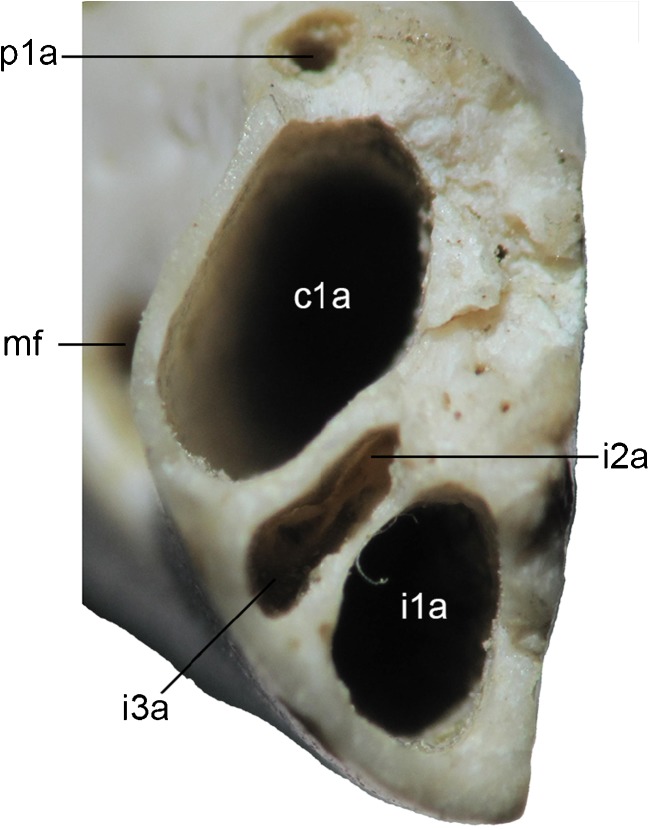
Partial right mandible of *Epidolops ameghinoi* (MNRJ 2880-V) in anterior view. Abbreviations: c1a = lower canine alveolus; i1a = first lower incisor alveolus; i2a = second lower incisor alveolus; i3a = third lower incisor alveolus; mf = mental foramen; p1a = first lower premolar alveolus

Particularly informative are DGM 171-M, a partial left dentary, and MNRJ 2880-V, a partial right dentary (Fig. 7). There is a single large ventral alveolus (Fig. 7: i1a) at the anterior end of both specimens, which presumably housed i1. Dorsolateral to this, is an elongate opening that is oriented dorsomedial to ventrolateral. This opening appears to bifurcate into two alveoli deep within the substance of the dentary (Fig. 7: i2a and i3a). Based on this, I propose that this opening housed two teeth: ventrolaterally, a procumbent tooth that corresponds to the lateral incisor that was originally present in the right dentary of DGM 321-M and was illustrated by Paula Couto (1952c. figs. 3, 5A, 6A, 7A); dorsomedially, a procumbent tooth the dorsal edge of which would have been slightly higher than the dorsal edge of i1, and the which would have been directed slightly more dorsally than either of the other two incisors.

The dorsomedial position of this latter tooth relative to the other two incisors is strongly reminiscent of the “staggered” i2 seen in most polyprotodont metatherians (Hershkovitz 1982, 1995), but with a far greater degree of “staggering.” Based on this arrangement, I therefore identify this tooth as i2, and conclude that *E. ameghinoi* retains i1–3. Under this interpretation, the two incisors illustrated by Paula Couto (1952c) are i1 and i3, rather than i1 and i2 as he suggested. Thus, I conclude that the complete lower dental formula of *Epidolops ameghinoi* is i1–3 c1 p1–3 m1–4. Based on the depth of their alveoli, the roots of the procumbent teeth (i1–3 c1) of *E. ameghinoi* are not particularly extensive within the mandibular ramus; their posterior extent within the mandible is limited by the enormous roots of p3.

## Probable Additional Material of *E. ameghinoi*

Based on craniodental specimens, *E. ameghinoi* is by far the most common named marsupialiform species known from Itaboraí (contra Ladevèze and Muizon 2010: 749), with 115 craniodental specimens known and a Minimum Number of Individuals (MNI) of 43 (Table 2); this represents ~38 % of the total number of marsipialiform craniodental specimens and ~33 % of the total marsupialiform MNI from the fauna. The next most abundant single species, the much smaller *Marmosopsis juradoi*, is less than half as common, with an MNI of 19 (Table 2). All other named marsupialiform species from Itaboraí have an MNI of 8 or less (Table 2). It therefore seems likely that *E. ameghinoi* is represented among the non-dental marsupialiform specimens from Itaboraí.

**Table 2:** Relative abundance of named marsupialiforms from the Itaboraí Fauna. Taxonomy, identity, and number of specimens follow Marshall (1978, 1981, 1982a, 1987), Goin and Oliveira (2007), Goin et al. (2009), Oliveira and Goin (2011, 2015), and Oliveira et al. (2016)

Species	total number of craniodental specimens	minimum number of individuals
*Epidolops ameghinoi*	115	43
*Marmosopsis juradoi*	40	19
*Protodidelphis mastodontoides* ^a^	16	4
*Monodelphopsis travassoi*	15	7
*Protodidelphis vanzolinii*	15	6
*Patene simpsoni*	14	5
*Gaylordia mater*	13	4
*Gaylordia macrocynodonta* ^b^	12	8
*Didelphopsis cabrerai*	10	3
*Mirandatherium alipioi*	10	7
*Itaboraidelphys camposi*	7	4
*Guggenheimia crocheti*	5	3
*Minisculodelphis modicum*	5	3
*Carolopaulacoutoia itaboraiensis*	4	2
*Derorhynchus singularis*	4	2
*Minisculodelphis minimus*	3	2
*Eobrasilia coutoi*	2	1
*Bobbschaefferia fluminensis*	1	1
aff. *Bobbschaefferia* sp.	2	1
*Carolocoutoia ferigoloi*	1	1
*Gashternia carioca*	1	1
*Guggenheimia brasiliensis*	1	1
cf. *Nemolestes* sp.	1	1
*Periprotodidelphis bergqvistae*	1	1
*Procaroloameghinia pricei*	1	1
*Riolestes capricornicus*	1	1
*Zeusdelphys complicatus*	1	1

^a^= “*Robertbutleria mastodontoidea*”

^b^includes *Gaylordia* “*doelloi*”

Two major types of non-dental marsupialiform material have been described from the site: petrosals (Ladevèze 2004, 2007; Ladevèze and Muizon 2010) and postcranial elements (Szalay, 1994; Szalay and Sargis, 2001). Of these, only Szalay’s (1994) “Itaboraí Metatherian Group” (IMG) tarsal morphotype VII has been referred to *E. ameghinoi*. However, available evidence suggests that a petrosal morphotype can also be tentatively referred to *E. ameghinoi*.

### Petrosals

Ladevèze (2004, 2007) and Ladevèze and Muizon (2010) identified eight marsupialiform petrosal morphotypes (Types I-VIII) from Itaboraí that can be distinguished based on both relative size and morphology. Ladevèze (2004, 2007) and Ladevèze and Muizon (2010) used phylogenetic and morphometric approaches to try to associate these petrosal morphotypes with marsupialiform taxa from Itaboraí that have been named based on dental specimens. The morphometric approach involved plotting the areas of M2, M3, m2, and m3 against the area of the petrosal promontorium for a range of modern and fossil metatherians, and then calculating predictive regression equations from these data that could then be applied to the named Itaboraí marsupialiforms (Ladevèze and Muizon 2010: fig. 5, table 3; Ladevèze 2007: fig. 1, table 2). Based on these regression equations, Ladevèze (2007) and Ladevèze and Muizon (2010) concluded that *E. ameghinoi* could not be associated with any of the eight petrosal morphotypes: its molars appeared to be too big for association with Types I and III-VIII, but too small for association with Type II (Ladevèze and Muizon 2010: table 3; Ladevèze 2007: table 2). I reach a different conclusion, based on two lines of evidence.

**Fig. 8:**
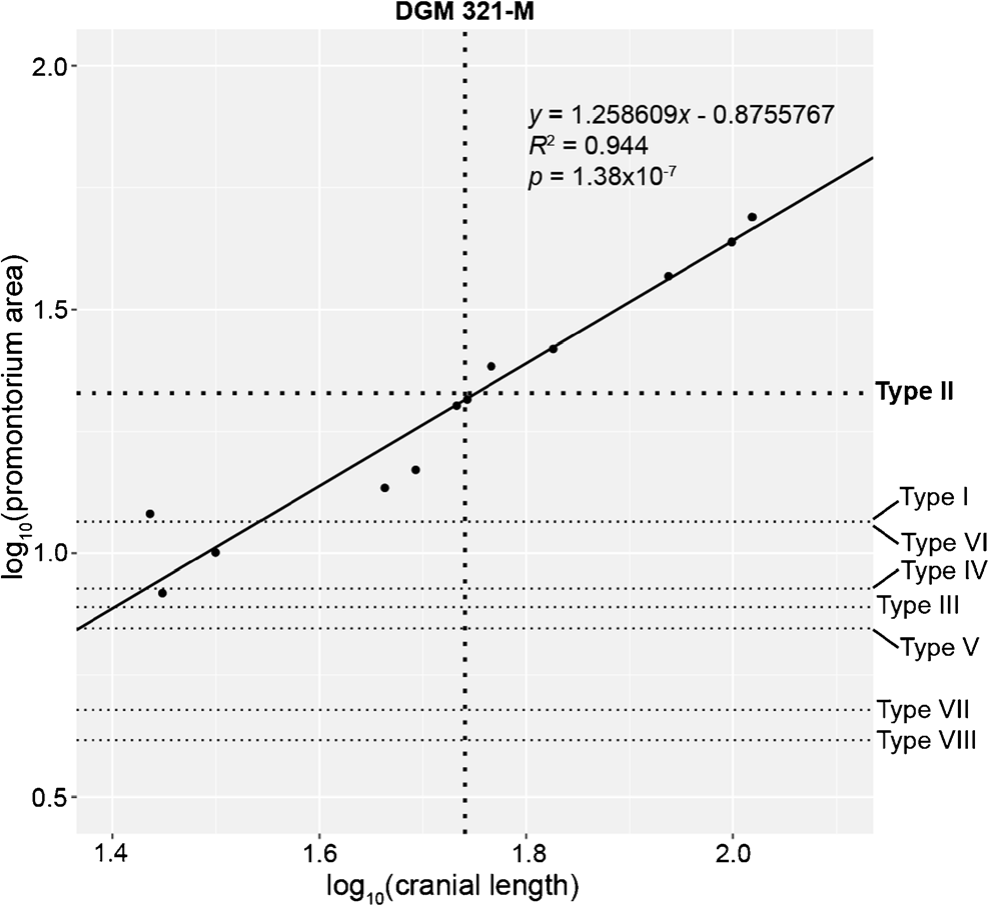
Reduced (‘standardized’) major axis regression of log_10_-transformed measurements of promontorium area against cranial length for 12 extant and fossil marsupialiforms (see Ladevèze and Muizon 2010: table 2 and electronic supplementary material). The solid line represents the line of best fit. The dotted horizontal lines represent the log_10_(promontorium area) of the eight petrosal morphotypes (Types I-VIII) from Itaboraí described by Ladevèze (2004, 2007) and Ladevèze and Muizon (2010). The dotted vertical line represents log_10_(estimated cranial length) of *Epidolops ameghinoi*, based on DGM 321-M. The Type II petrosal morphotype is suitably-sized for referral to *Epidolops ameghinoi*, whereas the other morphotypes are too small

Firstly, the molars of *E. ameghinoi* appear smaller relative to the overall size of its skull than those of most other metatherians (see Szalay 1994: table 6.3). Thus, *E. ameghinoi* should be expected to have petrosals with a larger promontorial area than predicted by the regression equations of Ladevèze (2007) and Ladevèze and Muizon (2010). As an alternative approach, I carried out reduced major axis regression of promontorium area of the 12 marsupialiform taxa used by Ladevèze and Muizon (2010) in their regression analyses, using cranial length as the predictor variable (see Fig. 8), rather than molar size. This analysis found a stronger correlation between promontorium area and total skull length (*R* ^2^ = 0.944; *p* = 1.38 × 10^−7^) than the correlations between promontorium area and molar size found by Ladevèze and Muizon (2010: fig. 5A-D), and gives the following regression equation: log_10_(promontorium area) = 1.258609*log_10_(cranial length) - 0.8755767. Assuming a total skull length of 55 mm based on DGM 321-M (see above), this gives an expected promontorium area of 20.6 mm^2^ for *Epidolops ameghinoi*; this value is almost identical to that of the Type II petrosals, namely 20.72–21.91 mm^2^ (Ladevèze and Muizon 2010: table 3; see Fig. 8).

Secondly, Ladevèze and Muizon (2010: 749) explicitly rejected relative abundance as a criterion for associating the petrosal morphotypes with dental taxa. However, I argue that relative abundance should be taken into account, particularly given that *E. ameghinoi* is by far the most common marsupialiform at Itaboraí (Table 2). Ladevèze and Muizon (2010) suggested that Type II belongs to either *Bobbschaefferia fluminensis* or *Procaroloameghinia pricei*; however, these two taxa are much rarer than *E. ameghinoi*, with both having an MNI of 1 (Table 2). Based on the combined evidence of relative size and relative abundance, I conclude that the Type II petrosals most likely belong to *E. ameghinoi*.

Ladevèze (2004) gave a detailed description of the Type II petrosals. They exhibit a number of features that are rare or absent in relative to crown marsupials, but which are found in the Tiupampan stem marsupials *Pucadelphys*, *Andinodelphys*, and *Mayulestes*. These include a tiny rostral tympanic process, a deep groove for the internal carotid artery at the anterior pole of the promontorium, and the posterior part of the hypotympanic sinus excavated in the petrosal lateral to the promontorium. Ladevèze and Muizon (2007: characters 162–165; 2010: characters 15 and 19) implied that this sinus is not homologous with the hypotympanic sinus; however, I interpret them as homologous based on their position and structural relations (see e.g., Muizon et al. 1997: fig. 2; Muizon 1999: fig. 4; Ladevèze and Muizon 2007: text-fig. 3). The similarity in petrosal structure between the Type II morphotype and *Pucadelphys*, *Andinodelphys*, and *Mayulestes* is particularly interesting given that all three Tiupampan taxa lack an ossified hypotympanic sinus floor, as also seems to be the case for *Epidolops* (see above); if, as I believe, the Type II petrosals belong to *E. ameghinoi*, then all four taxa appear to have a similar morphology of the auditory region that differs from all known crown marsupials.

### Tarsals

**Fig. 9:**
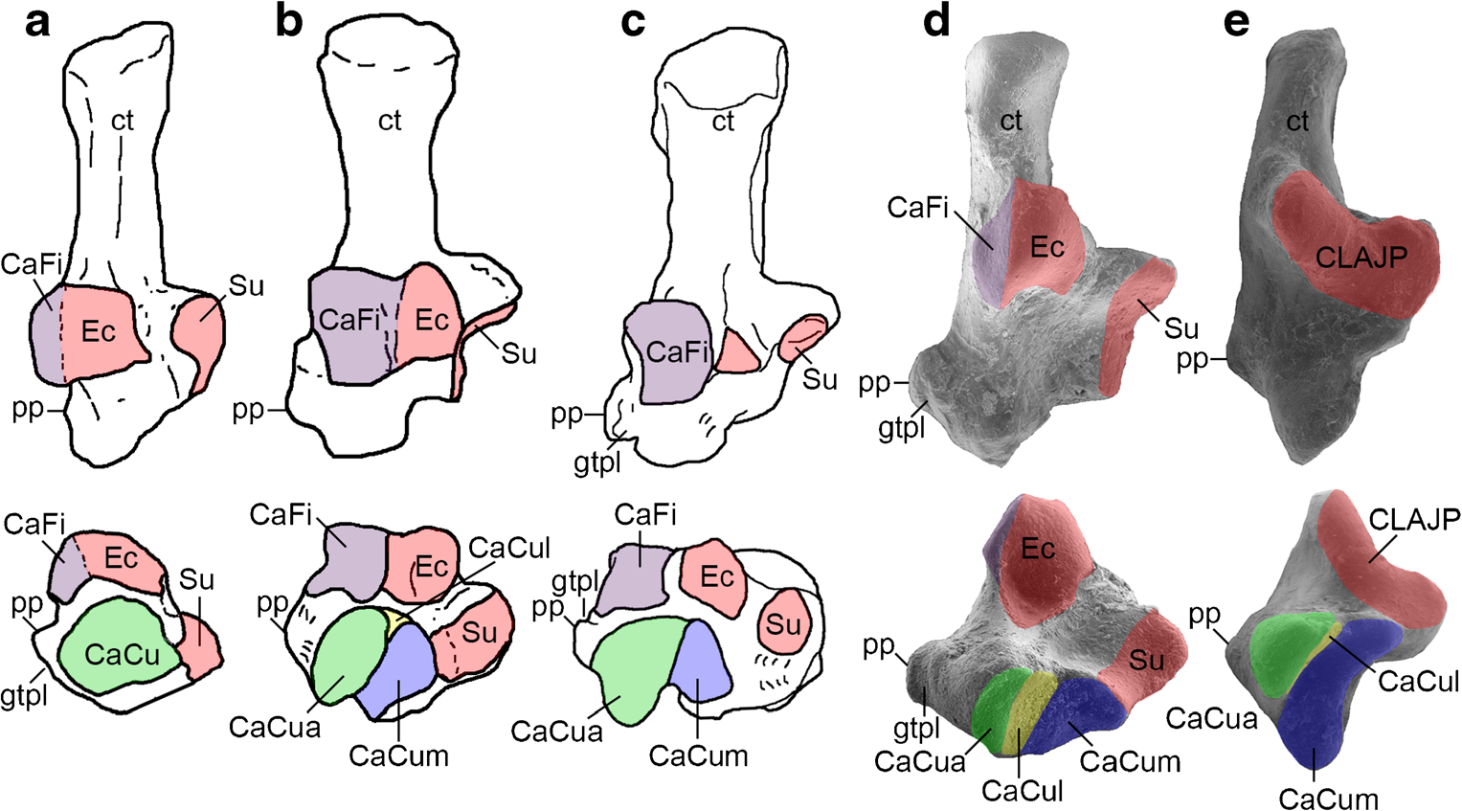
Isolated calcanea of a range of marsupialiforms in dorsal (flexad) and anterior (distal) views. **a** “Itaboraian Metatherian Group” (IMG) VII tarsal morphotype from Itaborai, which Szalay (1994) tentatively referred to *Epidolops ameghinoi* (redrawn from Szalay 1994: fig. 6.25); **b** Unnamed argyrolagid from the Colhuehuapian (early Miocene) Gaiman locality (redrawn from Szalay 1994: fig. 7.28); **c** *Argyrolagus scagliai* (redrawn from Szalay 1994: fig. 7.28); **d** *Caenolestes fuliginosus* (AMNH M-62915 – see Beck 2012); **e** *Dromiciops gliroides* (unregistered UNSW Palaeontology Laboratory specimen– see Beck 2012). Abbreviations: CaCu = calcaneocuboid facet; CaCua = auxiliary calcaneocuboid facet; CaCul = lateral calcaneocuboid facet; CaCum = medial calcaneocuboid facet; CaFi = calcaneofibular facet; CLAJP = continuous lower ankle joint pattern; ct = calcaneal tuber; Ec = ectal facet; gtpl = groove for the tendon of the peroneus longus muscle; pp. = peroneal process; Su = sustentacular facet. *Red* represents the ectal (Ec) and sustentacular (Su) facets or continuous lower ankle joint pattern (CLAJP – formed by fusion of the ectal and sustentacular facets); *green* represents the calcaneocuboid (CaCu) facet or auxiliary calcaneocuboid (CaCua) facet (which are probably homologous – see Szalay 1994; Beck, 2012); *yellow* represents the lateral calcaneocuboid (CaCul) facet; *blue* represents the medial calcaneocuboid (CaCum) facet. Specimens are not drawn to scale. Note that the lateral calcaneocuboid (CaCul) facet is perpendicular to the page and so is not visible in *Argyrolagus scagliai* (**c**; compare with **b**, **d** and **e**)

Szalay (1994) identified 12 distinct morphotypes among isolated marsupialiform tarsals from Itaboraí, which he referred to as “Itaboraí Metatherian Groups” (IMGs) I-XII. Of these, (Szalay 1994: 174–177) tentatively referred IMG VII to *E. ameghinoi*, because it is the second most abundant IMG (comprising 14 calcanea and two astragali) and it is compatible in size with the craniodental remains (the more common IMG II is far too small to be plausibly referred to *E. ameghinoi*). The IMG VII calcanea (Fig. 9a) are distinctly apomorphic: the peroneal process (Fig. 9a: pp) is very reduced, with the groove for the tendon of the peroneus longus muscle (Fig. 9a: gtpl) present on the ventral (rather than dorsal) side of this process, a prominent calcaneofibular facet (Fig. 9a: CaFi) is present lateral to the ectal facet (Fig. 9a: Ec), and the calcaneal tuber is elongate (Fig. 9a: ct).

On the other hand, the calcaneocuboid facet (Fig. 9a: CaCu) of IMG VII is a single surface, unlike the apomorphic bipartite calcaneocuboid facet characteristic of didelphids (Szalay 1982, 1994). The australidelphian apomorphies of a tripartite calcaneocuboid facet and merged ectal and sustentacular facets (= the continuous lower ankle joint pattern [CLAJP]) are also absent (Fig. 9e; Szalay 1982, 1994). The overall morphology of IMG VII is well-adapted for extensive flexion-extension, with little capacity for inversion or eversion of the foot, suggesting a terrestrial (perhaps cursorial) locomotor mode (Szalay 1994: 174–177).

## Comparisons with Other Taxa Currently Included in Polydolopimorphia

### Dentition

The occlusal morphology and cusp homologies of the molar dentition of *E. ameghinoi* and other taxa currently included in Polydolopimorphia (see Goin and Candela 2004; Case et al. 2005; Goin et al. 2010, 2016, in press) have been discussed at length in previous papers (Pascual and Bond 1981; Marshall 1982a; Goin and Candela 1995, 1996, 2004; Goin 2003; Goin et al. 2003a, 2010; Chornogubsky 2010; Zimicz 2011, 2014), and will not be repeated here. Instead, in comparing *E. ameghinoi* with other polydolopimorphians, I focus on other aspects of the dentition that show potentially phylogenetically informative variation.

### Upper Anterior Dentition

Assuming that incisors are lost from the posterior end of the incisor series (Ziegler 1971), the upper dental formula of *E. ameghinoi* was probably I1–3 C1 P1–3 M1–4, although the presence of one or two additional incisors (I4 and I5) cannot be completely ruled out (see above). There is no diastema between I3 and C1, and (based on its alveolus) C1 was clearly a large tooth.

It is unclear exactly how many upper incisors are present in the bonapartheriid *Bonapartherium hinakusijum*, but Pascual (1981) concluded that there were probably five. *Bonapartherium hinakusijum* also has a very large C1 that, based on the morphology in MMP 148, appears semi-procumbent (Pascual 1981: lamina I); however, this semi-procumbency may be an artefact, because this specimen is distinctly crushed dorsoventrally. The rosendolopid bonapartherioid *Hondonadia feruglioi* preserves at least four upper incisors (of which the posteriormost is the smallest), but the presence of five teeth cannot be ruled out (Goin and Candela 1998). *Hondonadia feruglioi* also preserves a very large, subvertical C1 (Goin and Candela 1998). Both *B. hinakusijum* and *H. feruglioi* exhibit a prominent diastema between the incisors and C1, within which there is a distinct paracanine fossa (Pascual 1981; Goin and Candela 1998). The lack of a diastema between the incisors and C1 in *E. ameghinoi* may be connected with the presence of a more procumbent c1 that presumably no longer fits between the incisors and C1 during occlusion.

Flynn and Wyss (2004) interpreted the upper dental formula of the polydolopid *Kramadolops mckennai* as I?1–2 C1 P1–3 M1–3, and argued that the three alveoli preserved at the anterior end of the only known cranium of *K. mckennai*, SGOPV 3476 (see Flynn and Wyss 2004: fig. 6.1), housed an incisor, C1, and P1. However, such an anterior position for P1 would be highly unusual among metatherians, and I think it more likely that the posteriormost alveolus of the three more likely housed an incisor or C1 (see also Chornogubsky 2010). Discovery of additional polydolopid specimens will be required to confidently infer their anterior dental formula.

Among argyrolagids, *Proargyrolagus bolivianus* exhibits four upper incisors (of which I1 appears by far the longest mesiodistally, followed by I2) and a very small canine, with no distinct diastema separating these teeth (Sánchez-Villagra and Kay 1997). *Argyrolagus scagliai* has only two teeth anterior to P3, namely two similarly-sized, apparently open-rooted incisors (presumably I1–2), followed by a very long diastema that seems to lack a paracanine fossa (Simpson 1970b). Species of *Groeberia* have two open-rooted upper incisors, immediately followed by a small C1 that Pascual et al. (1994) described as premolariform; no distinct diastema is present (Pascual et al. 1994: fig. 1D; Chimento et al. 2014: fig. 2A). *Klohnia charrieri* also has at least two (possibly open-rooted) upper incisors, of which I1 is the larger, but it is uncertain whether or not a canine or a diastema was present (Flynn and Wyss 1999).

### Lower Anterior Dental Formula

**Fig. 10:**
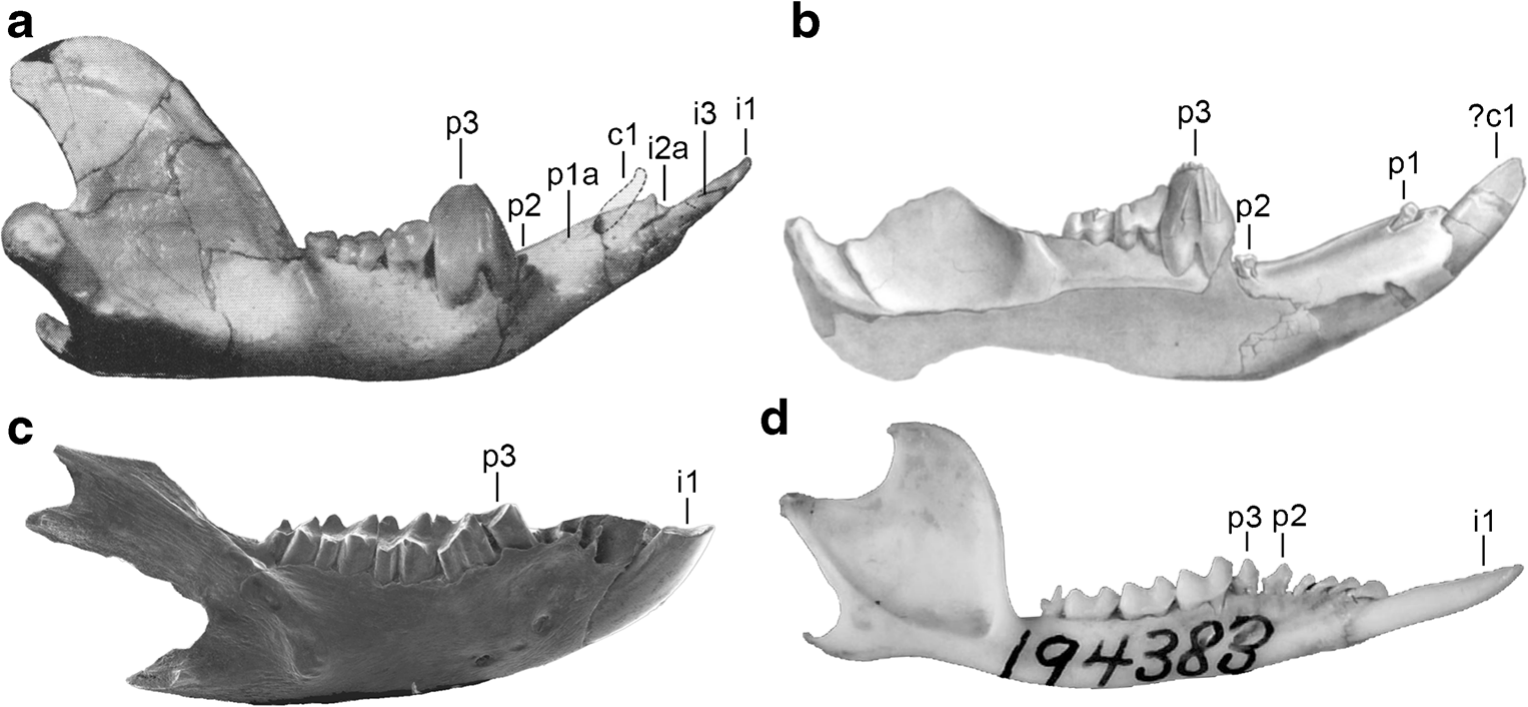
Dentaries of *Epidolops ameghinoi* and putative relatives. **a** *Epidolops ameghinoi* (DGM 321-M; modified from Paula Couto 1952: fig. 6A); **b** the polydolopid *Kramadolops abanicoi* (SGOPY 2941 [reversed]; modified from Flynn and Wyss 1999: fig. 1); **c** the argyrolagid *Anargyrolagus primus* (MPEF-PV 5299 [reversed]; modified from Goin and Abello 2013: fig. 4.18); **d** the caenolestid *Lestoros inca* (USNM 194383; modified from Martin 2013: fig. 4A). Abbreviations: c1 = lower canine; i1 = first lower incisor; i2a = second lower incisor alveolus; i3 = third lower incisor; p1 = first lower premolar; p1a = first lower premolar alveolus; p2 = second lower premolar; p3 = third lower premolar

I interpret the lower anterior dental formula of *E. ameghinoi* as i1–3 c1 (see above); this is in contrast to previous authors, who inferred a formula of i1–2 c1 (Paula Couto 1952c; Marshall 1982a). All four of these anterior teeth appear to be procumbent and enlarged, but i1 and c1 are markedly larger than i2–3 (Figs. 7 and 10a). Based on the arrangement of the alveoli, i2 appears to be staggered, as in deltatheroidans and most marsupialiforms (Hershkovitz 1982, 1995; Cifelli and Muizon 1997; Rougier et al. 1998; Kielan-Jaworowska et al. 2004), but with a much greater degree of staggering (Fig. 7).

Pascual (1981: 513) reported that there are four lower incisors in *Bonapartherium hinakusijum*, and that i2 appears to be staggered. Based on alveolar evidence, none of the lower incisors were enlarged or procumbent in this taxon (Pascual 1981). The c1 of *B. hinakusijum* is also well developed; Pascual (1981) concluded that this tooth was probably similar in size and orientation to the c1 of didelphids, implying that it is also non-procumbent in *B. hinakusijum*. Thus, *B. hinakusijum* cannot be described as diprotodont. Pascual’s (1980b) description of *Prepidolops didelphoides* indicates that at least three incisors were present in this taxon, that i2 was staggered, and that c1 is relatively well developed. Intriguingly, the anterior dentition of *P. didelphoides* is not procumbent in young individuals (e.g., MLP 78-V-6-1), but becomes increasingly procumbent with age (Pascual 1980b); that is to say, the degree of diprotodonty increases over the course of ontogeny. Assuming that *Bonapartherium* and *Prepidolops* are indeed polydolopimorphians, these taxa suggest that diprotodonty is not a synapomorphy of the order as a whole (unless *Bonapartherium* and *Prepidolops* have secondarily lost diprotodonty, which seems unlikely). The evidence from *Prepidolops*, with diprotodonty developing over ontogeny, is particularly intriguing, as this may represent an intermediate morphology between non-diprotodont and diprotodont dentitions; if so, it may give general insight into how diprotodonty arises in mammals.

Polydolopids have only a single enlarged and procumbent (“gliriform”) anterior tooth in the lower jaw (Fig. 10b); a critical, still-debated question is what locus this tooth represents (Marshall 1982a; Flynn and Wyss 1999, 2004; Chornogubsky 2010; Goin et al. 2010). The two most likely possibilities are i1 (Goin et al. 2010) and c1 (Marshall 1982a; Flynn and Wyss 1999, 2004); this is based on the general assumption that incisors are lost from the posterior end of the dental series, and on comparison with *E. ameghinoi*, in which both i1 and c1 are enlarged and procumbent (see above). The c1 of *E. ameghinoi* and the gliriform tooth of polydolopids are somewhat different in morphology (Figs. 6, 10a-b): the former is distinctly caniniform, with enamel restricted to the curved tip of the tooth, and it is less procumbent than i1 (Figs. 6, 10a; Paula Couto 1952c). By contrast the gliriform tooth of polydolopids such as *Kramadolops abanicoi* is not caniniform but is instead laterally compressed, and it also appears proportionately much larger than the c1 of *E. ameghinoi* (Fig. 10b; Flynn and Wyss 1999); however, it does not seem impossible that this morphology evolved from a more caniniform precursor.

A potentially key specimen for determining the homology of the polydolopid gliriform tooth is MACN 10340a, an edentulous partial right dentary of a polydolopid. This specimen was originally described as the holotype of “*Promysops acuminatus*” by Ameghino (1902), but Marshall (1982a) referred it to *Eudolops tetragonus*, and more recently Chornogubsky (2010) referred it to *Eudolops caroliameghinoi*. Marshall (1982a) reported that MACN 10340a preserves a large alveolus at its anterior end, with two much smaller alveoli present mesial to this. Ameghino (1903: Figs. 3 and 8) illustrated this specimen, but indicated the presence of only two alveoli: a larger alveolus laterally and a smaller alveolus medially. Assuming the more recent description of Marshall (1982a) is accurate, it implies that the gliriform tooth of MACN 10340a (and hence presumably of other polydolopids) is c1, with the two more mesial alveoli housing reduced incisors. Alternatively, one of these two alveoli might have housed two incisors (i2 and i3), as appears to be the case in *E. ameghinoi* (Fig. 7; see above). Ultimately, however, the complete absence of teeth in MACN 10340a means that its referral to *Eudolops tetragonus* is not assured, and the identity of the gliriform lower tooth of polydolopids remains contentious.

Based on tooth counts in *Proargyrolagus bolivianus* (see Sánchez-Villagra and Kay 1997) and *Anagyrolagus primus* (see Goin and Abello 2013), the gliriform lower tooth of argyrolagids is unequivocally an incisor, identified here as i1 (Fig. 10c). There are four small, single-rooted teeth between the gliriform incisor and p3 in the lower jaw in *Proargyrolagus bolivianus* (see Sánchez-Villagra and Kay 1997) and *Anagyrolagus primus* (Fig. 10c; Goin and Abello 2013), and hence there must be at least one additional incisor present in both of these taxa; assuming that incisors are lost from posterior to anterior, the tooth immediately posterior to i1 can therefore be identified as i2. Sánchez-Villagra and Kay (1997: 720) reported that, based on its alveolus, the i2 of *Proargyrolagus bolivianus* was probably procumbent and that it was “pressed along the side” of i1, suggesting that it may have been staggered, but this requires confirmation. The i2 of *Anagyrolagus primus* does not appear obviously staggered (Goin and Abello 2013: fig. 4.13–18). I consider the homologies of the remaining three teeth between i2 and p3 in *Proargyrolagus bolivianus* and *Anagyrolagus primus* to be unclear, but Goin and Abello (2013) interpreted them as c1 p1–2. In *Argyrolagus scagliai*, there is one tooth between the gliriform i1 and p3, which is small, single-rooted, procumbent and immediately behind i1 (Simpson 1970b); based on comparison with *Proargyrolagus bolivianus*, it seems likely that this tooth is i2, as was concluded by Simpson (1970b).

In the lower dentition of *Groeberia minoprioi*, a large, relatively vertically-oriented (rather than procumbent) tooth is present anteriorly, followed by a diastema, after which there are four molars (Pascual et al. 1994); the molars can be clearly identified as such because they retain clear evidence of a tribosphenic cusp pattern (Patterson 1952; Simpson 1970a). In the holotype of *Groeberia minoprioi*, MMP 738, there is no evidence of teeth within the lower diastema (Patterson 1952; Chimento et al. 2014). However, Pascual et al. (1994) concluded that there are two unicuspid teeth between the enlarged anterior tooth and m1 in another *G. minoprioi* specimen, MLP 85-IX-24-1. Subsequently, Chimento et al. (2014) argued that only a single unicuspid is present in this region in this specimen. By itself, this information is insufficient to identify the identity of the enlarged anterior lower tooth in *Groeberia minoprioi*; however, given that its two occlusal counterparts in the upper jaw are unequivocally incisors (I1–2; Simpson 1970a; Pascual et al. 1994; Chimento et al. 2014), it seems reasonable to conclude that it is also an incisor, i1. The identity of the unicuspid (where present) within the lower diastema (i.e., whether an incisor, canine, or premolar) is uncertain. Pascual et al. (1994) suggested that it might be c1; however, given that it is closely appressed to i1, it is tempting to identify it as i2, as assumed by Chimento et al. (2014). If it is i2, published studies do not clearly indicate whether or not it is staggered.

In the lower jaw of *Klohnia charrieri*, there is a single gliriform tooth followed by a large diastema and then p3; the diastema is reportedly entirely edentulous (Flynn and Wyss 1999). Two large, open-rooted incisors are present in the upper jaw of *Klohnia charrieri* (see Flynn and Wyss 1999), making it likely that the lower gliriform tooth is also an incisor, i.e., i1. The lower anterior dentition of the enigmatic *Patagonia peregrina* comprises a single lower gliriform tooth of uncertain homology, but which has been interpreted as incisor (Pascual and Carlini 1987; Goin and Abello 2013), immediately followed by a very small, single-rooted, somewhat procumbent tooth that was identified as a probable c1 by Pascual and Carlini (1987, see also Goin and Abello 2013) but whose homology is likewise uncertain; it may in fact be i2.

### Premolar Number and Morphology

**Fig. 11:**
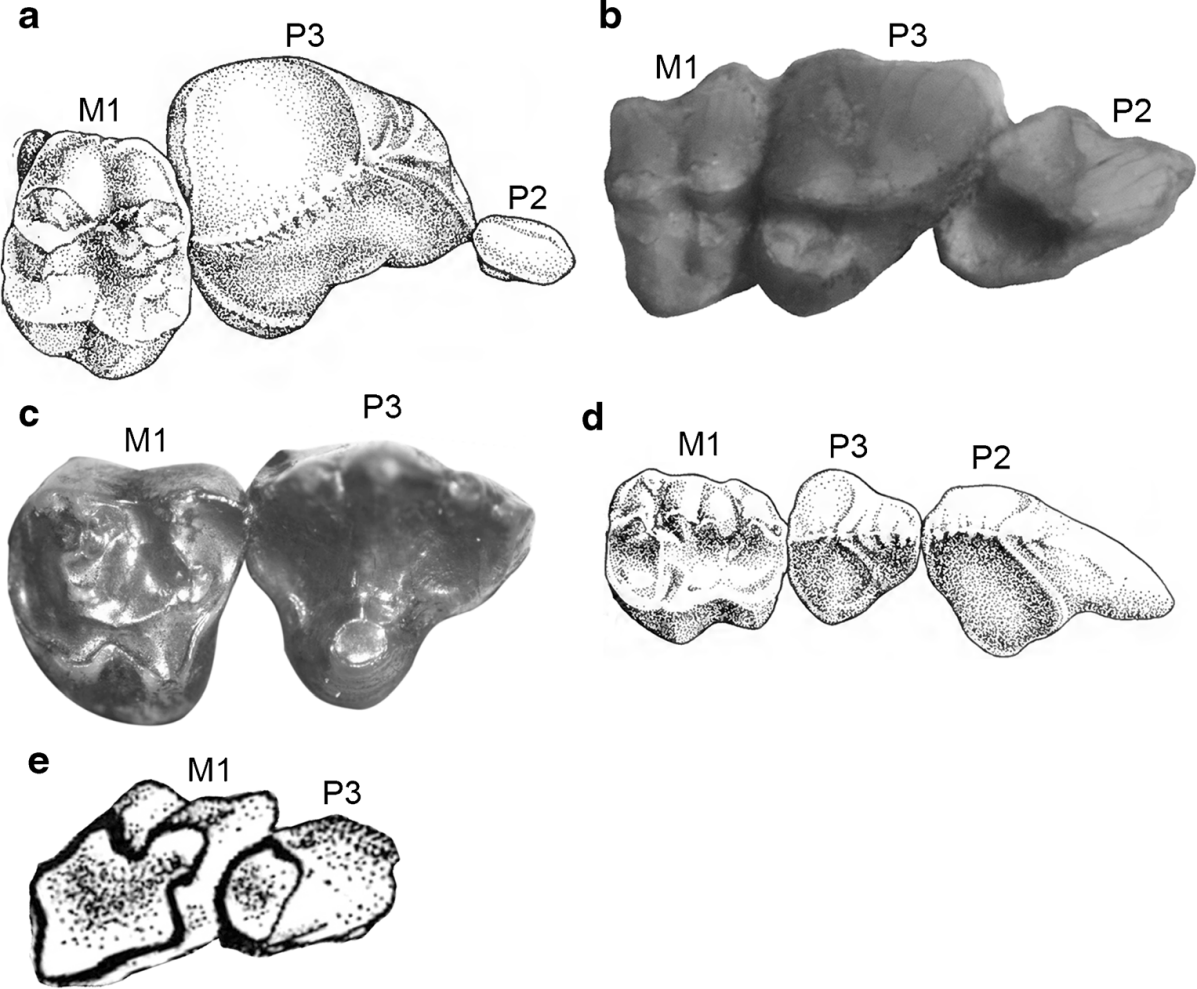
Upper premolars and first upper molar of *Epidolops ameghinoi* and putative relatives. **a** *Epidolops ameghinoi* (DGM 800-M; modified from Marshall 1982: fig. 67b); **b** the bonapartheriid *Bonapartherium hinakusijum* (MMP 1416); **c** the gashterniid *Gashternia carioca* (MCN-PV 1801 [reversed]; modified from Goin and Oliveira 2007: fig. 1); **d** the polydolopid *Polydolops thomasi* (MACN 10338; modified from Marshall 1982: fig. 32);. **e** the argyrolagid *Anargyrolagus primus* (MACN-ch-1305; modified from Carlini et al. 2007: fig. 2A). Abbreviations: M1 = first upper molar; P2 = second upper premolar; P3 = third upper premolar

In *E. ameghinoi*, P1 and p1 were clearly small, single-rooted teeth (although neither tooth is preserved in any known specimen), P2 and p2 are tiny and buttonlike, while P3 and p3 are enormously hypertrophied and plagiaulacoid (Figs. 2-4, 6 and 11a). Other polydolopimorphians show a diversity of premolar numbers and morphologies.

In *Bonapartherium hinakusijum*, P1 is small but double-rooted, whilst P2 and P3 are both large, three-rooted, and bladelike, with a distinct lingual platform or talon not present in *E. ameghinoi*; P3 is considerably wider and taller than P2 (Fig. 11b; Pascual 1980a, 1981). In the lower dentition of *B. hinakusijum*, p1 and p2 are much smaller and lower than the large, bladelike p3 (Pascual 1980a, 1981). Unlike *E. ameghinoi*, neither the P3 nor p3 of *B. hinakusijum* is truly plagiaulacoid sensu Simpson (1933), i.e., they lack a serrated edge (Fig. 11b). In *Hononadia feruglioi*, the morphology of P3 is unknown, but both P1 and P2 are small and premolariform, with P2 slightly larger (Goin and Candela 1998). *Hondonadia* (= “*Pascualdelphys*”) *fierroensis* (see Goin et al. 2010) is known from a single lower dentary, the premolars of which appear more plesiomorphic than those of other taxa currently placed in the order Polydolopimorphia: all three are double-rooted and they increase in size slightly from anterior to posterior (Flynn and Wyss 1999).

In the prepidolopids *Prepidolops didelphoides* and *Punadolops alonsoi*, P3 is the only upper premolar currently known; it is very large and bladelike but not plagiaulacoid, and it lacks a lingual platform or talon (Pascual 1980a, b; Goin et al. 1998a). In the lower dentition of prepidolopids, meanwhile, p1 is very small (based on *Prepidolops didelphoides*) and possibly single-rooted (based on *Prepidolops molinai*), p2 is somewhat larger and double-rooted, with the roots and crown oriented strongly obliquely relative to the major dental axis, and p3 is enormous, double-rooted, and similar in crown morphology to P3 (Pascual 1980b; Goin et al. 1998a;). P3 is also the only upper premolar known for *Gashternia carioca*: it is bladelike, and somewhat plagiaulacoid, with five cusps aligned along its labial margin, and a distinct shelf supporting a single blunt cusp is also present lingually (Fig. 11c; Goin and Oliveira 2007). Lower premolars of *Gashternia calehor* are not preserved in the only known specimen (AMNH 28533), but, based on their alveoli, p1 was small and single-rooted, p2 was larger, double-rooted, and implanted obliquely, and p3 was larger still and also double-rooted (Simpson 1948). In *Wamradolops tsullodon*, P3 is large and bladelike but apparently not plagiaulacoid (Goin and Candela 2004); a small posterolingual platform appears to be present, but it is far less developed than in either *Gashternia carioca* or *Bonapartherium hinakusijum*.

Based on SGOPV 3476, P1 must have been very small or entirely absent in the polydolopid *Kramadolops mckennai* (the alveolus that Flynn and Wyss 2004 argued as having housed P1 is more likely for an incisor or C1 – see above), and it has not been reported in other members of the family. Both P2 and P3 are well developed, double-rooted and plagiaulacoid in *K. mckennai*, with P2 the larger of the two (Flynn and Wyss 2004). Among other polydolopids, Marshall (1982a) reported that P2 is apparently absent but P3 is present and double-rooted in *Amphidolops serrula* (see also Simpson 1948: plate 6.5), whereas in *Eudolops tetragonus* P2 and P3 are similarly-sized, relatively small, double-rooted, and somewhat bladelike without being plagiaulacoid (see Marshall 1982a: fig. 58). In most polydolopids (e.g., *Polydolops thomasi*; Fig. 11d), however, P2 is considerably larger than P3, and both teeth are distinctly plagiaulacoid (Marshall 1982a; Chornogubsky et al. 2009).

In the lower dentition, comparison with *E. ameghinoi* persuades me that the lower postcanine formula proposed for the polydolopid *Kramadolops abanicoi* by Flynn and Wyss (1999) – namely p1–3 m1–3 – is correct. If so, the p1 of *K. abanicoi* is very similar in morphology to that of *E. ameghinoi*, being very small, single-rooted, and procumbent (Fig. 10b; Flynn and Wyss 1999). The p2 is present but very small and either double- or single-rooted in many polydolopids (e.g., *Kramadolops abanicoi* - Fig. 10b; *Polydolops* spp.; Marshall 1982a) but entirely absent in others (e.g., *Antarctolops* spp.; Chornogubsky et al. 2009). *Roberthoffstetteria nationalgeographica* (grouped with polydolopids in Polydolopiformes by Goin et al. 2016) has three double-rooted, premolariform premolars, of which P3 was probably slightly larger than P2 (Marshall et al. 1983; Muizon et al. 1984; Goin et al. 2003a). The lower premolars of *R. nationalgeographica* are less well known, but p3 appears to be double-rooted and premolariform (Muizon et al. 1984).

Among argyrolagids (e.g., *Anargyrolagus primus* – Fig. 11e), P3 and p3 are consistently present, small, and appear to be hypsodont or hypselodont, similar to the molars; with wear, they form a continuous dental series with the molars (Simpson 1970b; Sánchez-Villagra and Kay 1997; Goin and Abello 2013). Both *Anagyrolagus primus* (Fig. 11e) and *Proargyrolagus bolivianus* have a small, single-rooted P1 and P2 in the upper jaw, whilst in the lower jaw there are four small, single-rooted teeth between the gliriform incisor and p3, and hence there could be a total of one, two, or three lower premolars (Sánchez-Villagra and Kay 1997; Carlini et al. 2007; Goin and Abello 2013). In *Argyolagus scagliai*, there are no teeth between I2 and P3 in the upper jaw, whilst in the lower jaw there is one tooth between the gliriform incisor and p3, which is probably i2 (Simpson 1970b; see above).

*Groeberia minioproi* has three small, single-rooted upper premolars (i.e., P1–3) that differ little in size in the upper jaw (Pascual et al. 1994). As discussed, the holotype of *G. minioproi* lacks any teeth in the diastema between i1 and m1, and hence lower premolars are absent in this specimen, but a single-rooted unicuspid of uncertain homology is present in the diastema in MLP 85-IX-24-1 (Pascual et al. 1994; Chimento et al. 2014). Thus, at most *G. minioproi* sometimes retained a single lower premolar. However, Pascual et al. (1994) suggested that the unicuspid in MLP 85-IX-24-1 is a canine, and Chimento et al. (2014) argued that it is more likely an incisor; in either case, this would mean that lower premolars are consistently absent in *G. minioprioi*. Upper premolar number in *Klohnia charrieri* is uncertain based on available specimens, but P3 is clearly present; its occlusal morphology is unknown, but it clearly was not hypertrophied (Flynn and Wyss 1999). In the lower jaw, p3 is apparently the only lower premolar present: it is small, peg-like, and probably single-rooted, but it appears somewhat hypsodont and with wear its morphology resembles that of the p3 of argyrolagids (compare Flynn and Wyss 1999: fig. 2E with Simpson, 1970b: fig. 1A-C). Assuming that the three posteriorly-located, quadrilateral, hypselodont teeth in the upper and lower jaw of *Patagonia peregrina* are molars (i.e., M1–3 and m1–3), this taxon entirely lacks premolars (Pascual and Carlini 1987; Goin and Abello 2013).

### Molar Number

*Epidolops ameghinoi* retains four molars, but M4 and m4 are very small and single-rooted (Figs. 3, 4 and 6). Four molars are present in *Bonapartherium hinakusijum*, *Prepidolops didelphoides*, and *P. molinai*, but M4 and m4 are considerably smaller than M3 and m3 (Pascual 1980a, b, 1981). All known polydolopids lack the fourth molar (Marshall 1982a), as does the prepidolopid *Punadolops alonsoi* (see Goin et al. 1998a). *Hondonadia* (= “*Pascualdelphys*”) *fierroensis* retains four lower molars, with m4 double-rooted and only slightly smaller than m3 (Flynn and Wyss 1999). Argyrolagids retain four molars, with M4 and m4 usually markedly smaller than M1–3 and m1–3 (Fig. 10c; Simpson 1970b; Sánchez-Villagra and Kay 1997; Carlini et al. 2007; Goin and Abello 2013), and a similar morphology is seen in *Groeberia* (see Pascual et al. 1994). *Klohnia charrieri* has only three molars (Flynn and Wyss 1999), as does *Patagonia peregrina* (see Pascual and Carlini 1987; Goin and Abello 2013).

### Rostrum

*Epidolops ameghinoi* lacks a distinct masseteric process (for attachment of the superficial masseter) at the anterior end of the zygomatic arch (Figs. 2-4). A masseteric process also appears to be absent in *Bonapartherium hinakusijum*, but dorsoventral crushing of the best preserved cranial specimen, MMP 1408, means that this is uncertain (Pascual 1981: lamina I). The only known cranium of *Kramadolops mckennai* is too badly crushed to determine whether or not a masseteric process is present (Flynn and Wyss 2004: figs. 6.1–6.2). A distinct, raised masseteric process is, however, clearly present in argyrolagids (Simpson 1970b: fig. 4A-B) and *Groeberia* (see Pascual et al. 1994).

### Palate

*Epidolops ameghinoi* is characterized by a relatively imperforate palate, with a single pair of very small maxillopalatine fenestrae extending from level with the anterior margin of M1 to level with M2 (Fig. 4). Most definitive crown marsupials have comparatively much larger palatal vacuities, although their number, exact size, and sutural relations vary (Archer 1984a, b; Voss and Jansa 2009). A few, however, have a largely imperforate palate, among them the didelphid *Caluromys*, which is the modern marsupial that most closely resembles *E. ameghinoi* in terms of the probable size of its maxillopalatine fenestrae (see e.g., Bucher and Hoffmann 1980: fig. 2; Cáceres and Carmignotto 2006: fig. 2; Voss and Jansa 2009: fig. 38).

Among other polydolopimorphians, the palate of *Bonapartherium hinakusijum* appears very similar to that of *E. ameghinoi*, with a single, small, relatively centrally-placed pair of maxillopalatine fenestrae (Pascual 1981). Pascual (1981) stated that the fenestrae extend from P3 to M2 in *Bonapartherium hinakusijum*, but Fig. 4.1 of Zimicz (2014) and Fig. 5.10b of Goin et al. (2016) suggest that the fenestrae are damaged in the best preserved cranium of this specimen, MMP 1408, and that they may have only extended the length of M1–2 when intact, as in *E. ameghinoi*. Flynn and Wyss (2004: 85) stated that “a distinct foramen or vacuity occurs near the posterobuccal corner of the palate” in the polydolopid *Kramadolops mckennai*. In fact, this opening (clearly identifiable on the right [= anatomical left] side of SGOPV 3476 in Flynn and Wyss, 2004: fig. 6.1) appears to be the posterolateral palatal foramen (see Voss and Jansa 2009: fig. 14; Wible 2003: figs. 4, 5B). As illustrated (Flynn and Wyss 2004: fig. 6.1), the palate of SGOPV 3476 seems too badly damaged to determine whether or not there are true fenestrae; if present, however, such fenestrae must have been small. Other polydolopid specimens are insufficiently well preserved to determine whether or not palatal fenestrae are present.

Pascual et al. (1994) reported the presence of an elongate pair of palatal fenestrae in *Groeberia* spp., but they did not identify the bones enclosing them; however, they appear to be maxillopalatine fenestrae (pers. obs.). Elongate palatal vacuities are present in all known argyrolagids (Simpson 1970b; Sánchez-Villagra and Kay 1997; Sánchez-Villagra et al. 2000; Carlini et al. 2007; García-López and Babot 2015); in *Hondalagus altiplanensis* at least, they are between the maxilla and palatine (pers. obs.). The precise extent of these fenestrae is unclear in *Hondalagus altiplanensis*, but in *Proargyrolagus bolivianus*, *Anagyrolagus primus*, and *Argyrolagus scagliai* they appear to extend from P3 to M4 (Simpson 1970b; Carlini et al. 2007). As noted by Simpson (1970b: 22), a narrow median septum may have originally divided left and right fenestrae in *Argyrolagus scagliai*, but, if so, has broken away in known specimens; alternatively, a septum might have been absent, with the fenestrae forming a single, very large opening (this region is less well preserved in *Hondalagus altiplanensis*, *Proargyrolagus bolivianus* and *Anagyrolagus primus*). Finally, Goin and Abello (2013) reported that palatal fenestrae are absent in *Patagonia peregrina*, but more complete material is probably required to confirm this.

### Postpalatal Region

A notable apomorphy of the postpalatal region of *E. ameghinoi* is the presence of a very well-defined pterygoid fossa, with a distinct ectopterygoid crest laterally enclosing at least the anterior half of the fossa (Figs. 3 and 5); in most other metatherians, this fossa is shallow or indistinct, and lacks an obvious ectopterygoid crest. Known crania of most other polydolopimorphians are too poorly preserved to determine the morphology of the pterygoid fossa (Simpson 1970a; Pascual 1981; Pascual et al. 1994; Sánchez-Villagra and Kay 1997; Sánchez-Villagra et al. 2000; Flynn and Wyss 2004); however, this fossa does not appear to be well developed in the argyrolagid *Argyrolagus scagliai* (Simpson 1970b: fig. 3B).

The transverse canal foramen is either absent or (based on a tiny foramen present bilaterally within the pterygoid fossa) very small in *E. ameghinoi* (Figs. 3 and 5). Most other polydolopimorphians are insufficiently well-preserved to determine whether or not the transverse canal foramen is present, but this foramen is present and large in the argyrolagids *Hondalagus altiplanensis* (there are three foramina on the right side of MNHN-Pal-BoIV-006,330, but only one on the left side; Sánchez-Villagra et al. 2000) and *Argyrolagus scagliai* (Simpson 1970b).

### Auditory Region

**Fig. 12:**
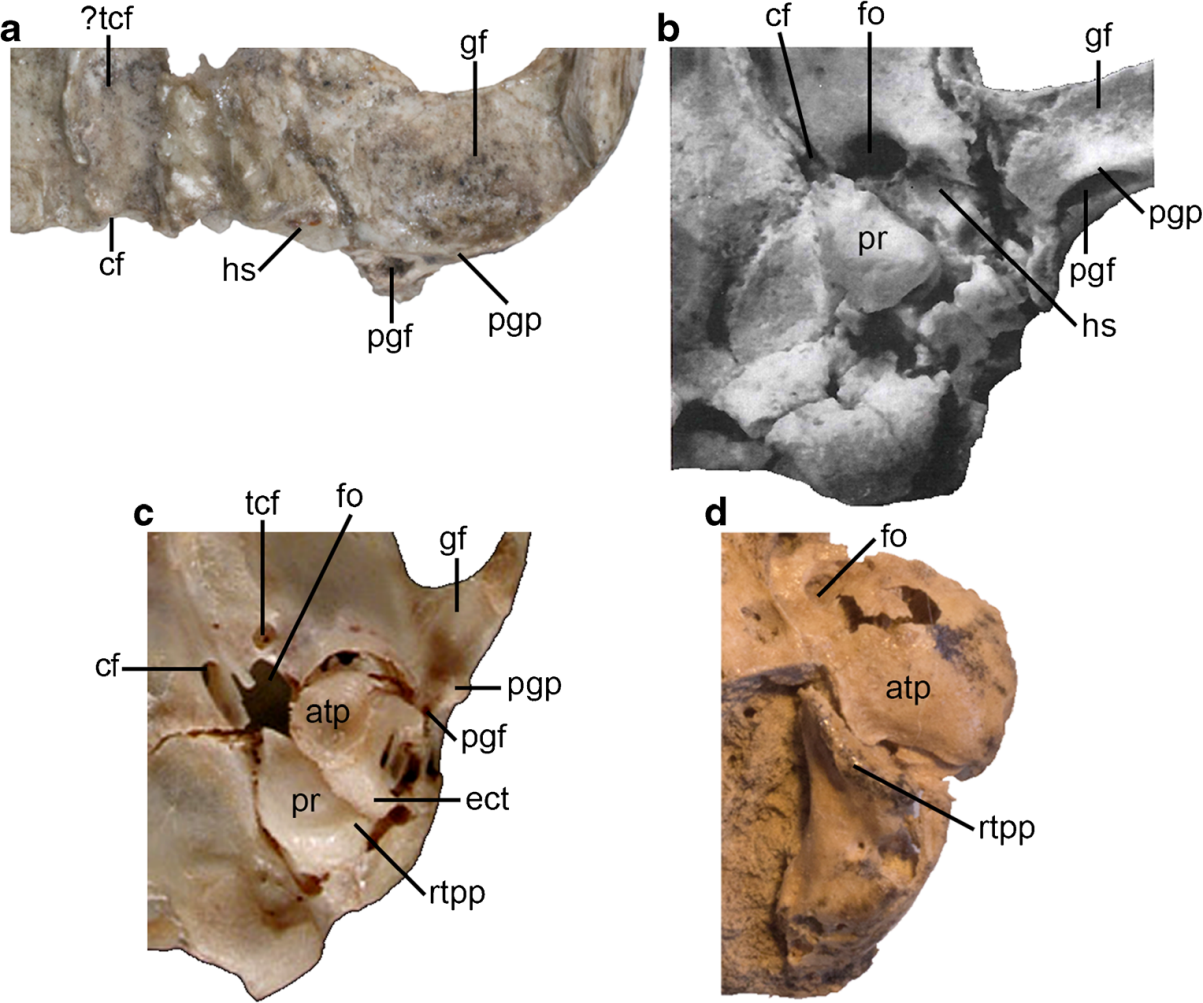
Basicranial region of *Epidolops ameghinoi* and other marsupialiforms. **a** *Epidolops ameghinoi* (DGM 321-M); **b** the mayulestid *Mayulestes ferox* (MHNC 1249; modified from Muizon 1998: fig. 8A); **c** the caenolestid *Caenolestes convelatus* (modified from Animal Diversity Web); **d** the argyrolagid *Argyrolagus scagliai* (MMP 5538 – specimen is damaged and is missing the occipital region). Abbreviations: atp = alisphenoid tympanic process; cf = carotid foramen; ect = ectotympanic; fo = foramen ovale; gf = glenoid fossa; hs = hypotympanic sinus; pgf = postglenoid foramen; pgp = postglenoid process; pr = promontorium of the petrosal; rtpp = rostral tympanic process of the petrosal; tcf = transverse canal foramen. Note that **a-c** are in ventral view, whereas **d** is in ventromedial view

The most striking aspect of the auditory region of *E. ameghinoi* is the apparent lack of an ossified floor to the hypotympanic sinus (see above; Fig. 12a). The morphology of the auditory region is unknown for most other polydolopimorphians. However, all known argyrolagids differ markedly from *E. ameghinoi* in possessing a large alisphenoid tympanic process flooring the hypotympanic sinus (Fig. 12d; Simpson 1970b; Sánchez-Villagra and Kay 1997; Sánchez-Villagra et al. 2000).

### Glenoid Region

The glenoid region of *E. ameghinoi* appears unspecialized (Figs. 3, 5 and 12a). The glenoid fossa forms a smooth curve, and the postglenoid process is well developed but unpneumatized. Complete loss of the ectotympanic in DGM 321-M suggests that this bone was neither fused nor tightly sutured to the postglenoid process (fusion of the ectotympanic with adjacent bones is observed in many diprotodontians; Aplin 1987, 1990; Springer and Woodburne 1989). The postglenoid foramen is immediately posterior to the postglenoid process, and was probably entirely enclosed by the squamosal in the intact skull. The only other group currently included in Polydolopimorphia for which the glenoid region is known is Argyrolagidae. Simpson (1970b: 24–25) reported that the glenoid fossa of *Argyrolagus scagliai* is “almost perfectly flat,” and that the ecotympanic resembles that of *Caenolestes*, namely forming an incomplete ring that is unfused to the adjacent bones. Simpson (1970b) did not describe the morphology of the postglenoid process, but he identified a foramen immediately dorsal to the external auditory meatus as a possible homologue of the postglenoid foramen (Simpson 1970b: 25); however, it seems more likely that this is in fact the subsquamosal foramen (= suprameatal foramen sensu Wible 2003), in which case the postglenoid foramen of argyrolagids has yet to be identified.

### Mandible

The overall morphology of the mandible of *E. ameghinoi* does not appear particularly derived, except for the presence of multiple small foramina within the masseteric fossa (Figs. 6 and 10a). Pascual (1980b, 1981) did not discuss the presence of masseteric foramina in *Prepidolops* spp. or *Bonapartherium hinakusijum*, nor are they mentioned in published descriptions of polydolopids, *Klohnia charrieri*, or *Patagonia peregrina* (Marshall 1982a; Pascual and Carlini 1987; Flynn and Wyss 1999; Goin et al. 2010; Goin and Abello 2013). Rusconi (1933, 1936) and Simpson (1970b: 20) both stated that a masseteric foramen is present in *Argyrolagus* spp., but it is not mentioned in descriptions of other argyrolagids in which this region is preserved (Sánchez-Villagra and Kay 1997; Goin and Abello 2013).

**Fig. 13:**
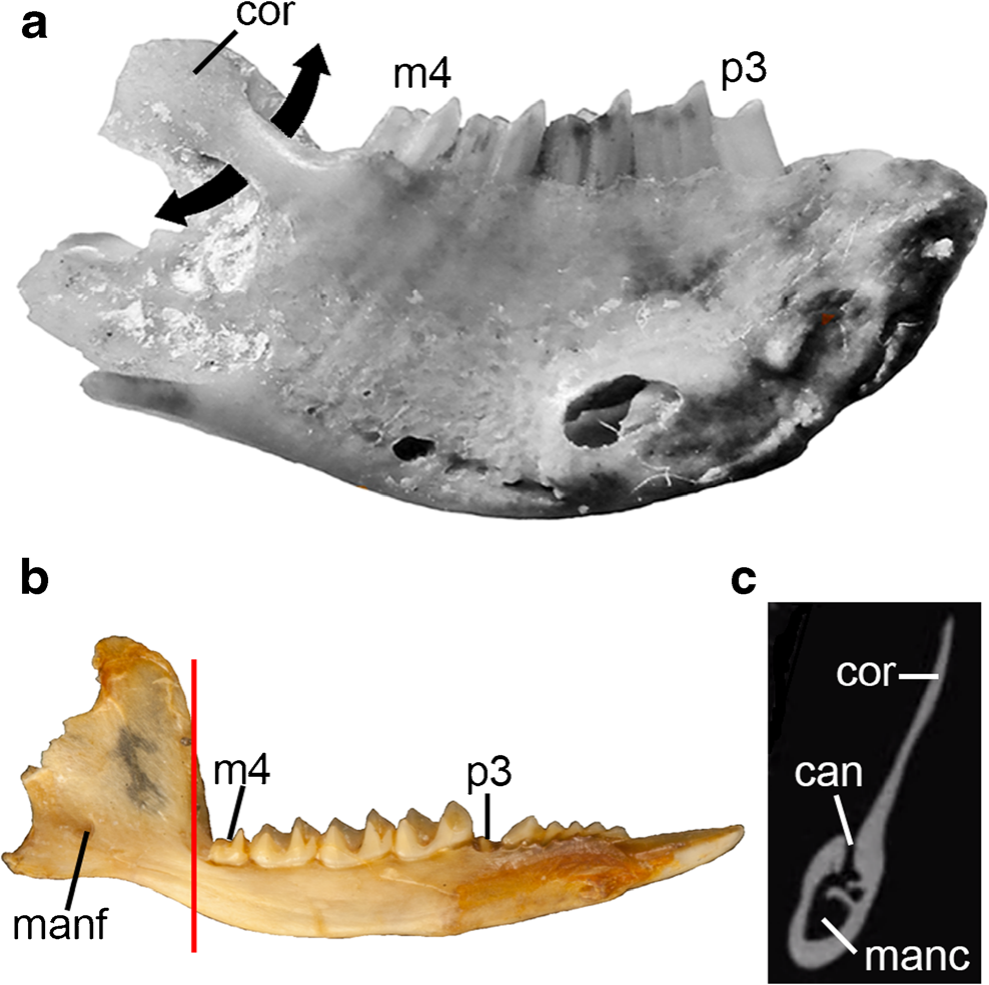
Comparison of the morphology of the retromolar space of the argyrolagid *Argyrolagus* and the caenolestid *Caenolestes*. **a** Partial left mandible of *Argyrolagus* sp. (MACN 17590) in medial view, with arrows indicating the path of the retrodental canal (= maxillary canal sensu Babot and García-López 2016; modified from Babot and García-López 2016: fig. 9.1); **b** Left mandible of *Caenolestes caniventer* (BMNH 1954.302) in medial view, with vertical red line corresponding to the plane of the coronal section shown in **c**; **c** coronal section of left mandible of *Caenolestes fuliginosus* (KU 124015) in anterior view, based on CT scan data (modified from Digimorph). Abbreviations: can = canal; cor = coronoid process; m4 = fourth lower molar; manc = mandibular canal; manf = mandibular foramen; p3 = third lower premolar

*Epidolops ameghinoi* lacks a retrodental canal sensu Hoffstetter and Villarroel (1974, = maxillary canal sensu Babot and García-López 2016), and this structure has not been reported in *Bonapartherium hinakusijum*, *Prepidolops* spp. or polydolopids (Pascual 1980b, 1981; Marshall 1982a). It is also clearly absent in *Groeberia* (pers. obs.). A very large retrodental canal is, however, consistently present in argyrolagids (Fig. 13a; Rusconi 1933; Simpson 1970b; Hoffstetter and Villarroel 1974; Sánchez-Villagra et al. 2000; Babot and García-López 2016) - Goin and Abello (2013) reported that a retrodental canal is absent in *Proargyrolagus*, but Sánchez-Villagra et al. (2000: 292) identified the canal in *Proargyrolagus bolivianus* after additional preparation of already-described material (Sánchez-Villagra and Kay 1997).

### Petrosal

As discussed above, Ladevèze’s (2004) Type II marsupialiform petrosals from Itaboraí plausibly represent *E. ameghinoi*. Notable features of this morphotype include the presence of a deep groove for the internal carotid artery at the anterior pole of the promontorium, presence of a very small rostral tympanic process, and an incomplete dorsal roof of the geniculate ganglion of the facial nerve (Ladevèze 2004).

Most other polydolopimorphians are not known from petrosal specimens, but the ventral surface of the petrosal has been described for the argyrolagids *Proargyrolagus bolivianus* and *Hondalagus altiplanensis*: in both taxa, the petrosal differs markedly from the Itaboraí Type II petrosals in having a prominent, anteroposteriorly elongate rostral tympanic process (Sánchez-Villagra and Kay 1997; Sánchez-Villagra et al. 2000). A similarly prominent rostral tympanic process is present in *Argyrolagus scagliai* (Fig. 12d). *Hondalagus altiplanensis* lacks a deep promontorial groove for the internal carotid artery (Sánchez-Villagra et al. 2000), as does *Aryrgolagus scagliai* (pers. obs.), but it is unclear whether or not this structure is present in *Proargyrolagus bolivianus* (see Sánchez-Villagra and Kay 1997).

### Tarsals

Notable features of the IMG VII tarsal morphotype that Szalay (1994) referred to *E. ameghinoi* include: a very small peroneal process that is positioned at the distal terminus of the calcaneus, and in which the groove for the tendon of the peroneus longus muscles is on the ventral (rather than dorsal) surface; an unspecialized calcaneocuboid facet; an elongate tuber; separate ectal and sustenacular facets with a distinct sulcus calcanei between them (= the separate lower ankle joint pattern [SLAJP]); and a calcaneofibular facet that is lateral to (and continuous with) the ectal facet (Fig. 9a).

Tarsal remains have also been described for argyrolagids (Simpson 1970b; Szalay 1994; Babot and García-López 2016; see Fig. 9b-c). These specimens show several derived similarities that are markedly different from IMG VII: the calcaneofibular facet is extremely broad, and in *Argyrolagus scagliai* and *Microtragulus bolivianus* it is largely isolated from (and much larger than) the ectal facet, whilst the sustentacular facet is small and faces almost directly medially (Fig. 9b and c). Although still not particularly well developed, the peroneal process of argyrolagids is larger than that of IMG VII and is set back from the distal end of the calcaneus, and the groove for the tendon of the peroneal longus is on the dorsal (not ventral) surface. Perhaps the most striking difference between the argyrolagid tarsals and IMG VII is the morphology of the calcaneocuboid facet: it is a single facet in IMG VII (Fig. 9a), whereas in argyrolagids it is tripartite and distinctly “stepped” (Fig. 9b and c), with a distally-facing proximal facet, a more distal facet that faces medially, and a distalmost face that faces distally (see also Simpson 1970b: 30–31; Szalay 1994).

## Affinities of *Epidolops* and Other Polydolopimorphians

There are three main hypotheses for the affinities of *Epidolops* and the other taxa currently included in Polydopimorphia, namely that they are closely related to paucituberculatans (Gregory 1910; Simpson 1928, 1948; Paula Couto 1952c; Simpson 1945; Aplin and Archer 1987; Marshall 1987), that they are closely related to microbiotherians and diprotodontians in the marsupial superorder Australidelphia (Goin et al. 1998b, 2009, 2016, in press; Goin 2003; Goin and Candela 2004; Oliveira and Goin 2006, 2011; Chornogubsky and Goin 2015), or that they are not closely related to any other marsupialiform order (Szalay 1994; Case et al. 2005). Here, I review these alternatives based on the evidence provided by the *E. ameghinoi* specimens from Itaboraí.

### Evidence for Paucituberculatan Affinities

A close relationship between polydolopimorphians and paucituberculatans was originally proposed based on the presence in both groups of an enlarged and procumbent (“gliriform”) anterior tooth in the lower jaw (Gregory 1910; Simpson 1928, 1948; Paula Couto 1952c; Simpson 1945). However, the antemolar dentition of *E. ameghinoi* and paucituberculatans differ markedly: specifically, *E. ameghinoi* has four large, procumbent teeth (i1–3 and c1, with i1 and c1 particularly large; Figs. 6, 7, and 10a) whereas paucituberculatans have only a single gliriform tooth followed by a series of very small unicuspids (Fig. 10d; Osgood 1921; Marshall 1980; Martin 2007; Voss and Jansa 2009; Abello 2013; Martin 2013). Among other polydolopimorphians, *Bonapartherium hinakuaijum* apparently lacks a true gliriform lower tooth (Pascual 1981), whilst in *Prepidolops didelphoides* procumbency of the anterior lower dentition develops over the course of ontogeny (Pascual 1980b). Assuming that *Bonapartherium* and *Prepidolops* form a clade with *Epidolops* and other taxa currently included in Polydolopimorphia (Goin et al. 2009; Chornogubsky and Goin 2015), and that the absence of true diprotodonty in *Bonapartherium* and *Prepidolops* is plesiomorphic rather than secondary, then this represents compelling evidence that diprotodonty evolved independently in Polydolopimorphia and Paucituberculata.

*Epidolops ameghinoi* has several foramina within the masseteric fossa, and a masseteric foramen is also present in some paucituberculatans (e.g., *Caenolestes* spp., *Lestoros inca*, and *Stilotherium dissimile*; Osgood 1921; Simpson 1970b; Voss and Jansa 2009). However, the presence of this foramen has not been reported in any other putative polydolopimorphian besides the argyrolagid *Argyrolagus* spp. (Simpson 1970b). Furthermore, a masseteric foramen is only variably present in some other paucituberculatans (e.g., *Palaeothentes* spp., *Rhyncholestes raphanurus*; Forasiepi et al. 2014), whereas it is consistently present in many diprotodontians, the dasyuromorphian *Myrmecobius fasciatus*, *Notoryctes* spp., *Yalkaparidon coheni*, and the microbiotherian *Microbiotherium gallegosense* (pers. obs.; Abbie 1939; Marshall 1982b; Beck et al. 2014). Thus, the presence of this foramen in *E. ameghinoi* does not constitute strong evidence for a close relationship with paucituberculatans.

The remainder of the cranium of *E. ameghinoi* appears more plesiomorphic than paucituberculatans and other crown marsupials in lacking an ossified hypotympanic sinus floor (Figs. 3, 5, and 12a). All known paucituberculatans have the hypotympanic sinus enclosed anteriorly and ventrally by an alisphenoid tympanic process (Fig. 12c; Osgood 1921, 1924; Patterson and Gallardo 1987; Goin et al. 2003b; Ojala-Barbour et al. 2013; Forasiepi et al. 2014), a morphology that is likely plesiomorphic for Marsupialia as a whole (Horovitz and Sánchez-Villagra 2003).

The very small palatal vacuities and absent or tiny tranverse canal foramen of *E. ameghinoi* are also unlike the morphology seen in paucituberculatans and most other marsupials, in which palatal vacuities are normally well developed and the transverse canal foramen is usually prominent (Fig. 12c; Osgood 1921, 1924; Patterson and Gallardo 1987; Sánchez-Villagra and Wible 2002; Goin et al. 2003b, 2007a; Martin 2013; Ojala-Barbour et al. 2013; Rincón et al. 2015); these features of *E. ameghinoi* may be plesiomorphies, although it should be noted there is considerable homoplasy in both features within Metatheria (see below).

If the Type II petrosals do indeed belong to *E. ameghinoi*, they share a few features with some paucituberculatans: like some specimens of *Caenolestes* spp. the dorsal roof for the geniculate ganglion is incomplete, and they share with specimens of *Lestoros inca* the presence of an anteroventral groove on the promontorium for the internal carotid artery (pers. obs.; Wible 1990; Ladevèze 2004). However, they differ in their tiny rostral tympanic process, which is a probable plesiomorphic feature; all known paucituberculatan petrosals exhibit a prominent rostral tympanic process (Fig. 12c; Sánchez-Villagra and Wible 2002; Goin et al. 2003b; Forasiepi et al. 2014).

If the IMG VII tarsals represent *E. ameghinoi*, then they also differ markedly from those of paucituberculatans (compare Fig. 9a and d). IMG VII is apomorphic in that the peroneal process is very small, with the groove for the tendon of the peroneus longus muscle on the ventral (rather than dorsal) surface, and the tuber is relatively elongate, but plesiomorphic in that the calcaneocuboid facet is a single facet (Fig. 9a; Szalay 1994); the very distal position of the peroneal process in also distinctive, but is of uncertain polarity. By contrast, calcanea of paucituberculatans (Fig. 9c) retain a well-developed peroneal process with a dorsal groove for the peroneus longus and a tuber that is not obviously elongate, whereas the calcaneocuboid facet appears distinctly tripartite, particularly in *Palaeothentes minutus* (Szalay 1982, 1994; Abello and Candela 2010).

Goin and co-workers (see e.g., Goin 2003; Goin et al. 2009) have shown that molar morphology differs markedly between polydolopimorphians and paucituberculatans, and they have concluded that their derived similarities, such as the presence of enlarged stylar cusps B and D and a metaconular hypocone sensu Beck et al. (2008a), evolved independently. This conclusion has been supported by published phylogenetic analyses (Goin et al. 2009; Forasiepi et al. 2013; Chornogubsky and Goin 2015), although these have focused almost exclusively on dental characters and have not incorporated the cranial or postcranial evidence discussed here.

### Evidence for Microbiotherian Affinities

Support for a close relationship between Polydolopimorphia and the extant South American australidelphian order Microbiotheria is based largely on the proposal that the polydolopimorphian molar pattern is derivable from a somewhat “microbiotherian-like” ancestor (Goin et al. 1998b, 2007b, 2016; Goin 2003; Goin and Candela 2004). However, molars of definitive microbiotherians and polydolopimorphians differ markedly in that the stylar cusps are reduced and labiolingually compressed (forming a crestlike structure along the labial margin of the tooth) in microbiotherians (Marshall 1982b; Goin et al. 2007b, 2016; Goin and Abello 2013), whereas stylar cusps B and D are distinctly enlarged in polydolopimorphians (Fig. 11a-d; Goin and Candela 1996; Goin 2003; Goin et al. 2016). Even the most plesiomorphic described microbiotherian, the middle Eocene *Woodburnodon casei*, appears far too derived in terms of its stylar shelf morphology to represent a plausible structural ancestor for polydolopimorphians (Goin et al. 2007b).

Centrocrista morphology also differs between the two groups: the centrocrista is straight in all known microbiotherians, whereas the centrocrista of polydolopimorphians (where identifiable) is open, with the postparacrista terminating at stylar cusp B and the premetacrista terminating at stylar cusp D (Fig. 11a-d; ; Goin 2003; Case et al. 2005; Goin et al. 2009: character 33). Finally, all known polydolopimorphians have an enlarged metaconule that is usually posterolingually displaced to form a metaconular hypocone sensu Beck et al. (2008a; see Fig. 11a-d), whereas all known microbiotherians have very reduced conules (Marshall 1982b; Goin et al. 2007b, 2016; Goin and Abello 2013). Thus, there are major differences in molar morphology between microbiotherians and polydolopimorphians, and on available evidence it seems more plausible to me that polydolopimorphians evolved from an ancestor with a more generalized marsupialiform molar morphology, namely in which the stylar cusps and conules were well developed, and the centrocrista was v-shaped.

Other aspects of the dentition and cranium of microbiotherians also differ markedly from those of *Epidolops* and other plesiomorphic polydolopimorphians. Microbiotherians are unusual in that i2 is not staggered (Hershkovitz 1982, 1995, 1999), whereas i2 appears to be staggered in several polydolopimorphians that preserve the anterior end of the mandible, including *E. ameghinoi* (Fig. 7), *Bonapartherium hinakusijum*, and *Prepidolops* spp. (see Pascual, 1980b, 1981). In the cranium, microbiotherians have large palatal vacuities and a complete auditory bulla that encloses the hypotympanic sinus, formed by an alisphenoid tympanic sinus and fused rostral and caudal tympanic processes of the petrosal (Hershkovitz 1999; Sánchez-Villagra and Wible 2002; Giannini et al. 2004), but they lack a groove for the internal carotid artery on the promontorium (Sánchez-Villagra and Wible 2002). As already noted, palatal vacuities are tiny in both *E. ameghinoi* and *B. hinakusijum*, whilst *E. ameghinoi* lacks an ossified floor to the hypotympanic sinus (Figs. 3, 5, and 12a), and, if the Type II petrosals belong to this taxon, also has a tiny rostral tympanic process of the petrosal and a distinct groove for the internal carotid artery (Ladevèze 2004).

Finally, in the tarsus, the microbiotherian *Dromiciops gliroides* exhibits the combination of a tripartite calcaneocuboid facet (also present in paucituberculatans – see above) and CLAJP (Fig. 9e; Szalay 1982, 1994) characteristic of australidelphians (Szalay 1982, 1994; Beck et al. 2008b; Beck 2012). The IMG VII tarsals, which are probably referable to *Epidolops*, lack both of these apomorphies (Fig. 9a; Szalay 1994).

### Evidence for Diprotodontian Affinities

A close relationship between Polydolopimorphia and the extant Australian order Diprotodontia has been proposed by Goin and co-workers (Goin 2003; Goin and Candela 2004). Recent phylogenies consistently place Diprotodontia and Microbiotheria in the clade Australidelphia, and some have supported a sister-taxon relationship between the two orders (see Beck in press-b for a review). The hypothesis that Polydolopimorphia and Microbiotheria are closely related (discussed above) implies that Polydolopimorphia must also be closely related to Diprotodontia. The shared presence of diprotodonty in polydolopimorphians and diprotodontians represents an obvious putative apomorphy supporting this relationship. I have already discussed why I believe a close relationship between Polydolopimorphia and Microbiotheria is unlikely (see above), which weakens support for Polydolopimorphia-Diprotodontia link.

A consideration of the craniodental and tarsal morphology of diprotodontians and polydolopimorphians also does not support this proposed relationship. Diprotodontians and most polydolopimorphians are diprotodont, but (as discussed above) the absence of diprotodonty in *Bonapartherium hinakusijum* and the ontogeny-related diprotodonty of *Prepidolops didelphoides* indicate that this derived feature must have originated independently in polydolopimorphians and diprotodontians – unless, that is, *Bonapartherium* and *Prepidolops* are early diverging members of a combined Polydolopimorphia + Diprotodontia clade. In addition, the craniodental anatomy of *E. ameghinoi* is very different and far more plesiomorphic than any diprotodontian. As already discussed, *E. ameghinoi* lacks an ossified hypotympanic sinus floor and the Type II petrosals that probably belong to this taxon lack a well-developed rostral tympanic process, whereas all diprotodontians have an at least partially ossified bulla (as do all crown marsupials – see below) and usually also a prominent rostral tympanic process of the petrosal (Archer 1984a; Aplin 1987, 1990; Springer and Woodburne 1989). In contrast to the Type II petrosals, a promontorial groove for the internal carotid artery is usually absent, although it is observed in a few taxa, e.g., some specimens of vombatids *Vombatus ursinus* and *Lasiorhinus latifrons* (Sánchez-Villagra and Wible 2002; Aplin 1987, 1990). Again unlike the Type II petrosals, the geniculate ganglion is roofed dorsally in all diprotodontians that I have examined with the exceptions of the macropodoid *Notamacropus agilis* and thylacoleonid *Thylacoleo carnifex* (pers. obs.).

In the glenoid region, most diprotodontians exhibit a distinct “complex” morphology, with a raised articular eminence anteriorly and grooved mandibular fossa, and the postglenoid foramen is usually in a medial position, often in the posteromedial corner of the glenoid fossa (Aplin 1987, 1990; Springer and Woodburne 1989). In *Epidolops*, the glenoid region is much more plesiomorphic: the glenoid fossa forms a continuous plane, and the postglenoid foramen opens posterior to the postglenoid process (Figs. 5 and 12a).

Tarsal morphology within Diprotodontia is highly variable, reflecting the variety of locomotor modes observed within the order. However, the tarsals of small-bodied “possums” such as burramyids, acrobatids, pseudocheirids and petaurids, closely resemble those of the microbiotherian *Dromiciops gliroides* (Fig. 9e) and likely approach the ancestral diprotodontian morphotype (Szalay 1982, 1994). The calcanea of these “possums” share with *Dromiciops* the apomorphies of a tripartite calcaneocuboid facet and CLAJP (Szalay 1982, 1994), neither of which are present in the IMG VII calcanea referred by Szalay (1994) to *Epidolops* (Fig. 9a).

### Evidence for a Position Outside Marsupialia

A third hypothesis for the affinities of Polydolopimorphia is that the order is not closely related to any other marsupial order. Indeed, available evidence suggests to me that *Epidolops*, and other taxa that are probably closely related such as *Bonapartherium*, *Prepidolops*, and polydolopids, most likely fall outside Marsupialia.

Most striking is the apparent absence of an ossified floor to the hypotympanic sinus in *E. ameghinoi*. Presence of an alisphenoid tympanic process flooring at least the anterior part of the hypotympanic sinus is probably plesiomorphic for Marsupialia (Horovitz and Sánchez-Villagra 2003). An ossified hypotympanic sinus floor formed by the alisphenoid is also present in some fossil metatherians that fall outside Marsupialia in recent published phylogenetic analyses; these include *Asiatherium* and the as-yet-named “Gurlin Tsav skull” from the Late Cretaceous of Mongolia (Szalay and Trofimov 1996), and *Herpetotherium fugax* from the early Oligocene of North America (Gabbert 1998; Sánchez-Villagra et al. 2007; Horovitz et al. 2008).

Several other non-marsupial metatherians, however, lack an ossified hypotympanic sinus floor, namely the Cretaceous Asian deltatheroidans (G.W. Rougier, pers. comm. in Forasiepi 2009; Bi et al. 2015: fig. 2E), *Pucadelphys*, *Andinodelphys*, and *Mayulestes* (Fig. 12b) from the early or middle Paleocene Tiupampa Fauna of Bolivia (Muizon 1994, 1998; Marshall and Muizon 1995; Muizon et al. 1997), the early Eocene North American peradectid *Mimoperadectes* (contra Horovitz et al. 2009 - see Horovitz et al. 2009: fig. S3 and comments by Beck 2012: electronic supplementary material and Jansa et al. 2014: supporting information) and many South American sparassodonts (Muizon 1999; Forasiepi 2009). Thus, the distribution of this feature within Metatheria is complex and shows some homoplasy. Nevertheless, the apparent absence of an ossified hypotympanic sinus floor in *E. ameghinoi* is a striking feature not seen in any crown marsupial.

Another potentially plesiomorphic feature of *E. ameghinoi* is its very small palatal fenestrae. Palatal morphology is variable among metatherians that have been found to lie outside Marsupialia in recent phylogenetic analysis: palatal fenestrae are absent in deltatheroidans (the sister-taxon of Marsupialiformes; Rougier et al. 1998; Bi et al. 2015), *Pucadelphys*, *Mayulestes*, and sparassodonts (Muizon 1994, 1998; Marshall and Muizon 1995; Forasiepi 2009; Engelman and Croft 2014; Forasiepi et al. 2015), but present in the “Gurlin Tsav skull,” various Late Cretaceous marsupialiforms (e.g., stagodontids; Fox and Naylor 1995, 2006), herpetotheriids, peradectids, and *Andinodelphys* (Muizon et al. 1997; Fox and Naylor 2006). This variability makes it difficult to determine the polarity of this feature. However, given their broad distribution among crown marsupials, it seems likely that the presence of well-developed maxillopalatine fenestrae is plesiomorphic for Marsupialia. The very small size of these fenestrae in *E. ameghinoi* (and in *Bonapartherium hinakuaijum* and probably also in *Kramadolops mckennai*; Pascual 1981; Flynn and Wyss 2004; Goin et al. 2016: fig. 5.10b) may be indicative of a position outside Marsupialia. However, it should be noted that many crown marsupials have secondarily lost or greatly reduced maxillopalatine fenestrae, such as caluromyine didelphids (Voss and Jansa 2009) and diprotodontoid diprotodontians (Archer 1984a), and reduction in the size of these fenestrae may also have occurred in polydolopimorphians.

A prominent transverse canal foramen is present in most marsupials and is likely plesiomorphic for Marsupialia, although the precise morphology and position of this foramen varies among marsupials (Sánchez-Villagra 1998; Sánchez-Villagra and Wible 2002; Horovitz and Sánchez-Villagra 2003). Among non-marsupial metatherians, a prominent transverse canal foramen is present in the *Herpetotherium fugax*, *Mimoperadectes houdei*, *Andinodelphys cochabambensis*, and probably also *Asiatherium reshetovi*, but it is absent in *Mayulestes ferox* (Fig. 12b) and probably also the “Gurlin Tsav skull” (Szalay and Trofimov 1996; Muizon et al. 1997; Muizon 1998; Sánchez-Villagra et al. 2007; Horovitz et al. 2008, 2009). Among sparassodonts, which also fall outside Marsupialia (Rougier et al. 1998, 2004; Forasiepi 2009; Engelman and Croft 2014; Forasiepi et al. 2015; Beck in press-b;), an obvious transverse canal foramen is absent in some taxa (e.g., *Arctodictis*) but present in others (e.g., *Prothylacynus*; Forasiepi 2009). The transverse canal foramen has been reported as absent in *Pucadelphys andinus* (see Marshall and Muizon 1995), but based on my own examination of a large collection of crania (see Ladevèze et al. 2011), a transverse canal foramen appears to be present in a few specimens. The condition in the sister-taxon to Marsupialiformes, Deltatheroida, is currently unknown. The transverse canal foramen is either absent or tiny in *E. ameghinoi*, in contrast to the prominent foramen observed in most marsupials; this may be an indication that it lies outside Marsupialia, but (as for palatal vacuities) the distribution of this feature shows considerable homoplasy.

The Type II petrosals that I argue probably belong to *E. ameghinoi* also appear more plesiomorphic than those of crown marsupials, most obviously in the very small rostral tympanic process and groove for the internal carotid artery (Ladevèze 2004); the published phylogenies that have included the Type II petrosals place them outside Marsupialia, either in a clade with the Tiupampan *Pucadelphys* and *Andinodelphys* (Ladevèze 2004, 2007; Ladevèze and Muizon 2010), or in an even more basal position within Marsupialiformes (Ladevèze and Muizon 2007).

Szalay (1994: 326) considered that the IMG VII tarsals from Itaboraí that he referred to *E. ameghinoi* to be derivable from a “primitive itaboraiform” ancestral morphology. Szalay’s (1994) concept of “Itaboraiformes” is explicitly paraphyletic, representing a grade from which Marsupialia presumably originated; an “itaboraiform” ancestry for *Epidolops* is therefore compatible with a position close to, but outside, Marsupialia.

One striking probable tarsal apomorphy of Marsupialia is complete superposition of the astragalus on the calcaneus (Szalay 1984, 1993; Horovitz 2000); in non-marsupial metatherians such as the deltatheroidan *Deltatheridium* and the marsupialiforms *Pucadelphys*, *Andinodelphys*, *Mayulestes*, and *Herpetotherium*, the astragalus is positioned more medially relative to the calcaneus, as indicated by the orientation of the ectal and sustentacular facets, and is in greater contact with the substrate (Horovitz 2000; Szalay and Sargis 2001, 2006; Horovitz et al. 2008). It is therefore noteworthy that the sustentacular facet of the IMG VII calcanea faces distinctly medially, particularly at its anterior end (Fig. 9a), implying incomplete superposition by the astragalus. The angle formed by the ectal and sustentacular facets also suggests incomplete superposition (Fig. 9a).

Presence of a very large astragalar medial plantar tubercle is another indicator that the astragalus is not completely superposed on the calcaneus (Szalay 1993, 1994; Szalay and Sargis 2001); Szalay (1994:176–177) identified two astragali (DGM 1.148-N and 1.151-M) as belonging to IMG VII, but did not illustrate or describe them in detail, and hence the size of the astragalar medial plantar tubercle is unclear. However, Szalay (1994: 176–177) reported that these two astragali are very similar in morphology to most of the other Itaboraian marsupialiform astragali, which suggests that the astragalar medial plantar tubercle in these specimens was large (Szalay 1994; Szalay and Sargis 2001). If, as this evidence suggests, *Epidolops* lacked complete astragalar superposition, it would provide further support for a position outside Marsupialia.

## Australidelphian Tarsals from the Early-Middle Eocene La Barda Locality

Lorente et al. (2016) described isolated australidelphian-type tarsals from the early-middle Eocene (Lutetian) La Barda locality in Patagonia, and argued that they belong to one of four taxa currently included in Polydolopimorphia, namely either *Gashternia* (Gashterniidae), *Polydolops* (Polydolopidae), *Amphidolops* (Polydolopidae), or *Palangania* (family incertae sedis). The La Barda tarsals differ markedly from the IMG VII that Szalay (1994) referred to *E. ameghinoi*, and they were placed within Diprotodontia in Lorente et al.‘s (2016) phylogenetic analysis. This suggests that either IMG VII or the La Barda tarsals (or possibly both) do not belong to the dental taxa to which they have been tentatively referred, or that Polydolopimorphia as currently recognized is polyphyletic.

*Gashternia* and the polydolopids *Polydolops* and *Amphidolops* share with *Epidolops* the apomorphic presence of an enlarged and bladelike P3 (Figs. 11a, c-d), and I have already discussed additional craniodental similarities between *Epidolops* and *Kramadolops*, which is currently the best known polydolopid genus (see “Comparisons with Other Taxa Currently Included in Polydolopimorphia” above). Premolar morphology is unknown for *Palangania*, and several dental similarities between this taxon and microbiotherians have been noted (Goin et al. 1998b; Goin 2003). If IMG VII represents *E. ameghinoi*, then I consider *Palangania* to be the most plausible candidate for referral of La Barda tarsals, out of the four taxa suggested by Lorente et al. (2016). However, this remains speculative in the absence of associated postcranial remains of the taxa under consideration.

## Affinities of Argyrolagoids

The superfamily Argyrolagoidea includes some of the dentally most derived taxa currently placed within Polydolopimorphia, namely the families Argyrolagidae, Groeberiidae, Patagoniidae, together with *Pradens*, *Klohnia*, and *Epiklohnia*, which are currently classified as Argyrolagoidea incertae sedis (Goin et al. 2010, 2016; Zimicz 2011). Recently, Chimento et al. (2014) argued that *Groeberia* and *Patagonia* are not polydolopimorphians or even therian mammals, but are in fact members on the non-therian order Gondwanatheria. A full reassessment of their work is beyond the scope of the current paper; however, I do not accept their conclusions, as briefly summarized here.

Perhaps most importantly, *Groeberia* retains obvious traces of a tribosphenic molar pattern (Patterson 1952; Simpson 1970a), whereas gondwanatherians are non-tribosphenic (Gurovich 2006; Krause 2014). In addition, the phylogenetic analysis of Chimento et al. (2014) is flawed because it does not include any other polydolopimorphians besides *Groeberia* and *Patagonia*. Particularly problematic is the absence of argyrolagids, which share with *Groeberia* and *Patagonia* a hypsodont or hypselodont molar dentition and a gliriform lower incisor, but which are nevertheless unequivocally marsupialiform based on their cranial morphology (Simpson 1970b; Sánchez-Villagra and Kay 1997; Sánchez-Villagra et al. 2000). With argyrolagids (and other key groups with superficially similar dentitions, such as rodents) absent from Chimento et al.‘s (2014) matrix, it is unsurprising that *Groeberia* and *Patagonia* ended up grouping with the only hypsodont/hypselodont taxa with a procumbent lower incisor present, namely gondwanatherians. I prefer the interpretation of most recent authors (e.g., Szalay 1994; Kirsch et al. 1997; Goin et al. 2016, in press), namely that *Groeberia* and *Patagonia* are marsupialiforms, although their precise affinities will remain unclear without the discovery of more complete material.

Argyrolagids are by far the best known argyrolagoids, represented by multiple crania and also postcranial remains, in addition to plentiful dental material (Rusconi 1933; Simpson 1970b; Hoffstetter and Villarroel 1974; Villarroel and Marshall 1988; Szalay 1994; Sánchez-Villagra and Kay 1997; Sánchez-Villagra et al. 2000; Sánchez-Villagra 2001; Carlini et al. 2007; Garcia-Lopez and Babot 2015; Babot and García-López 2016). Comparison of these specimens with known material of *E. ameghinoi* reveals numerous major differences.

As discussed above, the cranium of *E. ameghinoi* exhibits a number of strikingly plesiomorphic features, including very small maxillopalatine fenestrae (as also seen in *Bonapartherium hinakuaijum* and probably also the polydolopid *Kramadolops mckennai*; Pascual 1981; Flynn and Wyss 2004; Goin et al. 2016: fig. 5.10b), the hypotympanic sinus appears to lack an ossified floor, and the petrosal (if the Type II petrosals represent *Epidolops*) lacks a rostral tympanic process but has a deep groove for the internal carotid artery. By contrast, argyrolagids are far more derived: the maxillopalatine fenestrae are enormous (possibly forming a single, confluent opening in the palate), the hypotympanic sinus is enclosed ventrally by a very large alisphenoid tympanic process, and the petrosal has a prominent, elongate rostral tympanic process but lacks a deep groove for the internal carotid artery (Fig. 12d; Simpson 1970b; Sánchez-Villagra and Kay 1997; Sánchez-Villagra et al. 2000; Babot and García-López 2016).

In the mandible, *E. ameghinoi* has four large, procumbent teeth anteriorly (i1–3 and c1) and lacks a retrodental foramen (Figs. 6, 7, and 10a), whereas in argyrolagids the only procumbent tooth is a gliriform incisor, followed by one or more very small unicuspids, and a very large retrodental foramen is present (Figs. 10c and 13a; Rusconi 1933; Simpson 1970b; Hoffstetter and Villarroel 1974; Sánchez-Villagra and Kay 1997; Sánchez-Villagra et al. 2000; Sánchez-Villagra 2001; Carlini et al. 2007; Goin and Abello 2013; Babot and García-López 2016). Based on its alveolus, the C1 of *E ameghinoi* was clearly a very large tooth (as in *Bonapartherium hinakuaijum* and *Hondonadia feruglioi*; Pascual 1981; Goin and Candela 1998), whereas C1 of argyrolagids is either very small or entirely absent (Simpson 1970b; Sánchez-Villagra and Kay 1997; Sánchez-Villagra et al. 2000; Carlini et al. 2007). The P3 and p3 of *E. ameghinoi* are enormous and bladelike (Figs. 2, 3, 6, 10a, and 11a), as they are in several other polydolopimorphians, including *Bonapartherium hinakuaijum* (Fig. 11b), *Prepidolops* spp., *Gashternia carioca* (Fig. 11c), and polydolopids (Fig. 11d), although the precise morphology differs somewhat between taxa (Pascual 1980a, b, 1981; Marshall 1982a; Goin and Oliveira 2007). By contrast, P3 and p3 of argyrolagids are small, hypsodont or hypselodont teeth (Figs. 10c, 11e, and 13a; Rusconi 1933; Simpson 1970b; Hoffstetter and Villarroel 1974; Sánchez-Villagra and Kay 1997; Sánchez-Villagra et al. 2000; Sánchez-Villagra 2001; Carlini et al. 2007; Goin and Abello 2013) that would appear more easily derived from a generalised premolariform morphology than from enlarged bladelike precursors. Finally, the IMG VII tarsal morphotype referred *E. ameghinoi* by Szalay (1994) differs markedly from known argyrolagid tarsals, most notably in that the calcaneocuboid facet of *Epidolops* is a single facet whereas that of argyrolagids is more derived in being tripartite and distinctly stepped (Fig. 9a-c).

A prominent foramen is present in the retromolar space of most paucituberculatans that I have examined, namely *Caenolestes*, *Lestoros*, *Palaeothentes*, *Stilotherium*, and some but not all specimens of *Rhyncholestes* (see also Simpson 1970b; Voss and Jansa 2009; Ojala-Barbour et al. 2013), and it also seems to present in *Abderites* (Abello and Rubilar-Rogers 2012: fig. 6.4). CT scans demonstrate that this foramen leads into an elongate canal that extends ventrally and connects to the mandibular canal, within the substance of the dentary (Fig. 13c). Sánchez-Villagra et al. (2000: character 13) scored this foramen and canal in *Lestoros* as homologous with the retrodental canal of argyrolagids (Fig. 13a). However, the retromolar canal of argyrolagids is very short and does not connect with the mandibular canal, but instead opens on the medial surface of the dentary, dorsal to the mandibular foramen (Fig. 13a). In addition to the retrodental canal, Babot and García-López (2016) reported the presence of small foramina in the retromolar space (immediately posterior to m4) in argyrolagids; they argued that these small foramina, and not the retrodental canal, are homologous with the foramen in the retromolar space observed in paucituberculatans.

Scattered tiny foramina are present within the retromolar fossa of several other marsupials (pers. obs.), but these appear to be nutrient foramina that are variable in number and position, and which do not lead into a distinct canal, unlike the single, relatively large foramen observed in paucituberculatans. It is unclear whether the small retromolar foramina in argyrolagids lead into a distinct canal; if they do not, I consider it more likely that the distinct retromolar foramen and canal found in most paucituberculatans is homologous with the retrodental canal of argyrolagids (contra Babot and García-López 2016), despite the differences in position and morphology (see Brocklehurst et al. 2016 for a discussion of the homology of a morphologically similar canal, which they refer to as the “coronoid canal,” in afrotherian placentals). If these structures are indeed homologous, then they would represent a striking potential synapomorphy uniting paucituberculatans and argyrolagids. However, for the phylogenetic analysis presented here, I have elected to score the argyrolagids *Argyrolagus* and *Proargyrolagus* as having a retrodental canal, and the paucituberculatans *Caenolestes* and *Palaeothentes* as unknown for this character.

A tripartite calcaneocuboid facet is also present in paucituberculatans and australidelphians (Fig. 9d-e; Szalay 1982, 1994; Horovitz and Sánchez-Villagra 2003; Beck 2012). This feature has been identified as an australidelphian apomorphy (Szalay 1982, 1994; Szalay and Sargis 2006; Beck et al. 2008b; Beck 2012), but Horovitz and Sánchez-Villagra (2003: 185, fig. 3) noted that a tripartite calcaneocuboid facet is shared by paucituberculatans and australidelphians. Szalay and Sargis (2006: 205) downplayed this apparent resemblance, writing that “the alleged special similarity of the cuboid proximal surface of *Caenolestes* and australidelphians is so completely out of context of the well-understood dynamics of the entire tarsal character complex of these taxa that it is difficult to comment on.” Szalay and Sargis (2006) did not provide any specific evidence in support of this conclusion, however, and comparison of the calcaneocuboid facet of the calcanea of *Caenolestes* with a range of australidelphians reveals obvious similarities in morphology (see Fig. 9d-e). The fossil paucitiberculatan *Palaeothentes* also has a tripartite calcaneocuboid facet (Abello and Candela 2010: 1523, fig. 8D).

The position of the root within Marsupialia has yet to be confidently resolved. However, the retroposon analysis of Gallus et al. (2015) found statistically significant support for Didelphimorphia to be the first living order to diverge, leaving Paucituberculata and Australidelphia as sister-taxa. A tripartite calcaneocuboid facet is a therefore a potential morphological synapomorphy for Paucituberculata + Australidelphia, with the fossil argyrolagids included within this clade. The tripartite calcaneocuboid facet morphology appears to have evolved from a didelphimorphian-like bipartite precursor (Szalay 1994; Beck 2012), supporting the hypothesis that Didelphimorphia was the first living order to diverge within Marsupialia (Gallus et al. 2015). Thus, the tarsal morphology of argyrolagids is congruent with a close relationship to paucituberculatans.

In summary, there is no compelling morphological evidence that argyolagids are closely related to *Epidolops* and craniodentally similar forms such as *Bonapartherium* and polydolopids, which (as discussed above) may fall outside Marsupialia. Instead, the known anatomy of argyrolagids suggests that they are crown marsupials, and closely related to, or within, Paucituberculata. If so, Polydolopimorphia sensu *Goin* et al. (2016) is polyphyletic. This conclusion is somewhat similar to that of Szalay (1994) and Kirsch et al. (1997) who placed Argyrolagidae and also Gashterniidae, Groeberiidae, and Patagoniidae closer to undoubted paucituberculatans than to polydolopimorphians such as *Epidolops* and polydolopids (see Table 1). However, I consider that Gashterniidae is probably more closely related to *Epidolops* (and dentally similar forms such as *Bonapartherium* and polydolopids) than to argyrolagids, based on the shared presence in *Epidolops* and *Gashternia* of a large, bladelike P3. Szalay (1994: 335) also argued that argyrolagids may have originated from “pichipilin caenolestines,” but I do not think there is sufficient evidence at present to link argyrolagids with a specific paucituberculatan clade.

Sánchez-Villagra (2001) presented the first formal phylogenetic analysis of argyrolagids, using dental, cranial, and postcranial characters, and found that they formed the sister-taxon of caenolestids (other paucituberculatan families were not included), with strong support; the results of this analysis are congruent with my own conclusions. The phylogenetic analyses of Goin et al. (2009) and Chornogubsky and Goin (2015), by contrast, supported monophyly of Polydolopimorphia sensu Goin et al. (2016), i.e., including argyrolagids.

However, Goin et al. (2009) and Chornogubsky and Goin (2015) relied almost exclusively on dental characters, and interpreting the molar morphology of argyrolagids is difficult: their molars of are highly derived and, because they are hypsodont or hypselodont, their occlusal morphology is soon lost through wear (Simpson 1970b; Sánchez-Villagra et al. 2000; Zimicz 2011; Goin and Abello 2013). To my knowledge, the only argyrolagid molar to be described to date that is sufficiently unworn to preserve distinct cusps is an m1 of *Proargyrolagus bolivianus* (see Goin and Abello 2013: figs. 1.14 and 4.1); all other specimens have lost their cusps through wear, and hence uncertainties remain regarding cusp homologies (Sánchez-Villagra and Kay 1997; Zimicz 2011; Goin and Abello 2013). A priori interpretations regarding cusp homologies that lack clear evidential support may bias phylogenetic analyses towards particular topologies (O’Meara and Thompson 2014). Assumptions that, for example, the labialmost cuspid of the trigonid in argyrolagids is a neomorphic “ectostylid” (as in Zimicz 2011; Goin and Abello 2013) rather than the protoconid (as in Sánchez-Villagra and Kay 1997), remain questionable in the absence of unworn argyrolagid dentitions that would clarify the topological and occlusal relations between cusps.

Ultimately, the hypothesis of polydolopimorphian polyphyly will require further testing. CT data from argyrolagid crania are likely to prove particularly useful, as will the discovery of more complete remains (ideally including associated poscranial material) of other polydolopimorphians. The affinities of argyrolagids could also potentially be tested by molecular data: the youngest known argyolagids are from the late Pliocene (Marplatan South American Land Mammal Age; Goin et al. 2016), ~3.3–2.0 MYA, which is considerably older than the oldest successfully sequenced DNA (~430 kya; Dabney et al. 2013; Meyer et al. 2014, 2016) but younger than the oldest collagen peptide sequences (Rybczynski et al. 2013) obtained from fossils to date. Thus it may be possible to obtain collagen peptide sequences from late Pliocene argyolagids. If my hypothesis is correct, such sequences should form a clade with those from the living paucituberculatan caenolestids. Alternatively, if Goin et al. (2016) are correct, they should be more closely related to those of the living microbiotherian *Dromiciops* and diprotodontians, within the clade Australidelphia.

## Results of Phylogenetic Analyses

**Fig. 14:**
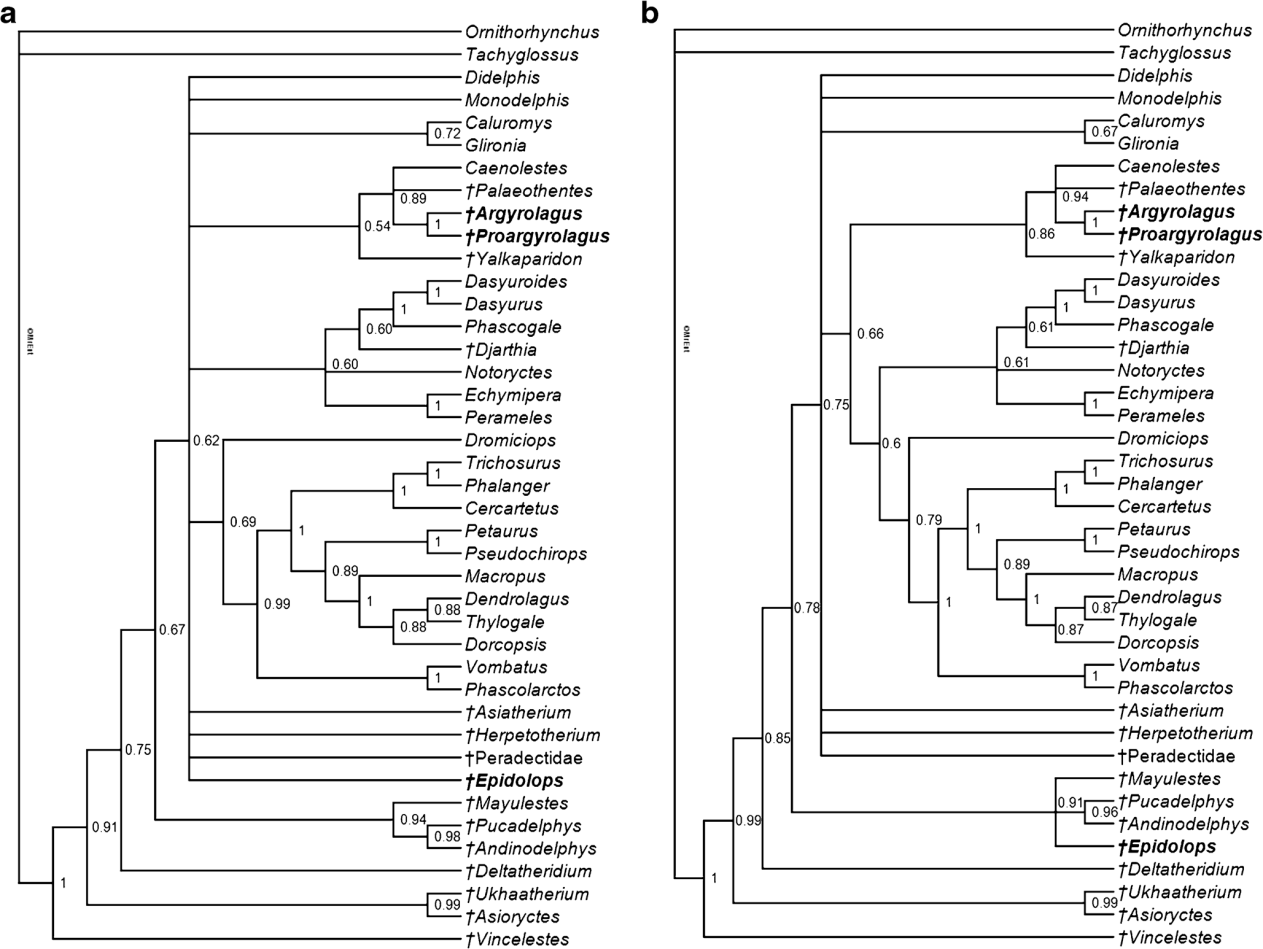
Phylogenetic relationships of *Epidolops*, argyrolagids, and other metatherians, based on Bayesian undated analyses of a total evidence matrix modified from Beck et al. (2014); both analyses comprised four independent runs of 50 × 10^6^ generations each, sampling trees every 2000 generations, and discarding the first 25 % (i.e., 12.5 × 10^6^ generations) as burn-in. **a** 50 % majority rule consensus of post-burn-in trees from analysis of matrix in which the Type II petrosals and IMG VII tarsals from Itaboraí were not used to score characters for *Epidolops* (“Matrix A”); the harmonic mean of lnL across all four runs was −62,311.01; **b** 50 % majority rule consensus of post-burn-in trees from analysis of matrix in which the Type II petrosals and IMG VII tarsals from Itaboraí were used to score characters for *Epidolops* (“Matrix B″); the harmonic mean of lnL across all four runs was −62,336.95. Values at nodes are Bayesian posterior probabilities. Extinct taxa are identified by *daggers*, and *Epidolops* and the argyrolagids are highlighted in *bold*

The results of the Bayesian non-clock total evidence analyses are shown in Fig. 14. Although taxon sampling is limited, the results for both versions of the total evidence matrix (i.e., either using the Type II petrosals and IMG VII tarsals to score characters for *Epidolops* or not) are congruent with the qualitative comparisons presented above. Most significantly, Polydolopimorphia sensu Goin et al. (2016) is diphyletic in both analyses, and *Epidolops* does not form a clade with either paucituberculatans (represented here by the extant caenolestid *Caenolestes* and the fossil palaeothentid *Palaeothentes*), microbiotherians (represented here by the extant microbiotheriid *Dromiciops*), or diprotodontians.

When the Type II petrosals and IMG VII tarsals are not used for scoring *Epidolops*, the resultant phylogeny is highly unresolved (Fig. 14a) with *Epidolops* part of a large polytomy that includes crown marsupials and several probable stem marsupials (*Asiatherium*, *Herpetotherium*, Peradectidae). Argyrolagidae is monophyletic (BPP = 1.00) and forms a relatively strongly-supported clade (BPP = 0.89) with the paucituberculatans *Caenolestes* and *Palaeothentes*, congruent with the earlier analysis of Sánchez-Villagra (2001). The Australian *Yalkaparidon* is sister to this clade, albeit with weak support (BPP = 0.54).

When the Type II petrosals and IMG VII tarsals are used to score *Epidolops*, the resultant phylogeny is somewhat more resolved (Fig. 14b). *Epidolops* is in a clade with *Mayulestes*, *Andinodelphys*, and *Pucadelphys* (BPP = 0.89), all of which are from the early or middle Paleocene Tiupampa fauna of Bolivia. As in the other analysis (Fig. 14a). Argyrolagidae is monophyletic (BPP = 1.00) and forms a clade with the paucituberculatans *Caenolestes* and *Palaeothentes* (BPP = 0.90), with *Yalkaparidon* sister to this clade (BPP = 0.88). A full list of the synapomorphies supporting the clades present in both phylogenies (under both accelerated and delayed transformation) is given in the Electronic Supplementary Material.

## Implications for the Biogeographical Origin and Early Evolution of Marsupialia

Recent molecular divergence dates (e.g., Beck 2008; Meredith et al. 2009, 2011; Mitchell et al. 2014) suggest that Marsupialia had originated and the modern orders had diverged from each other prior to the current estimate for the age of the Itaboraian, namely 50–53 Ma; as such, crown marsupials could, in principle, be present at Itaboraí. As already discussed, I conclude that *Epidolops* is not a crown marsupial. But what about the other marsupialiform taxa? Voss and Jansa (2009) observed that loss of the postcingulid from the lower molars optimizes as a synapomorphy of Marsupialia, although a postcingulid is present in dasyurids, thylacinids, and the stem australidelphian *Djarthia murgonensis*, indicating a degree of homoplasy in this feature (Beck in press-b). Nevertheless, the presence of a postcingulid in several of the Itaboraí marsupialiform taxa (e.g., *Bobbschaefferia fluminensis, Gaylordia mater, Minusculodelphis modicum, Protodidelphis mastodontoides*; Oliveira and Goin 2011, 2015; Oliveira et al. 2016) suggests that they may fall outside Marsupialia. This conclusion receives support from recent studies indicating that *Gaylordia* is a stem marsupial (Oliveira and Goin 2015) and that *Minisculodelphis* cannot be confidently placed within Marsupialia (Oliveira et al. 2016).

Several other Itaboraí marsupialiforms lack a postcingulid (e.g., *Guggenheimia crocheti*, *Procaroloameghinia pricei*, *Protodidelphis vanzolinii*; Oliveira and Goin 2011), and so are better candidates for being members of Marsupialia. However, loss of the third trochanter of the femur characterizes crown marsupials except paucituberculatans (given my proposal that argyrolagids are probably members of Paucituberculata, it is interesting to note that the femur of the argyrolagid *Argyrolagus scagliai* has a third trochanter; Simpson 1970b) and a few Australian taxa that are secondarily specialized for fossoriality (Szalay and Sargis 2001; Horovitz et al. 2008; Abello and Candela 2010; Beck et al. 2016). Szalay and Sargis (2001) described ten marsupialiform femur morphotypes, all of which exhibit a distinct third trochanter, suggesting that they represent stem marsupials or possibly paucituberculatans.

*Riolestes capricornis* was described as a paucituberculatan based on a single lower molar from Itaboraí (Goin et al. 2009), but this specimen may in fact be a dp3 of another, already named marsupialiform taxon (indeed, this possibility was considered by Goin et al. 2009). Szalay (1994) proposed that *Carolopaulacoutoia* (=“*Sternbergia*”) *itaboraiensis* may be a paucituberculatan, but subsequent studies have disagreed with this conclusion (Goin 2003; Oliveira and Goin 2011). The peculiar *Deroryhnchus singularis* has large and procumbent anterior incisors (Paula Couto 1952b; Marshall 1987), and Goin et al. (2009: 872–873) noted that derorhynchid molar morphology “anticipates” that seen in paucituberculatans. Congruent with this, the phylogenetic analysis of Goin et al. (2009), which was based on craniodental characters, placed this taxon as sister to Paucituberculata. Szalay (1994:333) also observed that the IMG 1 tarsal morphotype, which is of an appropriate size for referral to *D. singularis*, “shares some of the primitive [tarsal] pattern with caenolestids.” However, *D. singularis* also has an enlarged, procumbent canine (Paula Couto 1952b; Marshall 1987), whereas this tooth is greatly reduced or absent in definitive paucituberculatans (Marshall 1980; Martin 2007, 2013; Abello 2013). In addition, Forasiepi and Rougier (2009) tentatively referred an isolated metatherian petrosal (MPEF-PV 2235) from the early Paleocene (Clyde et al. 2014) Punta Peligro locality in southern Argentina to *Derorhynchus* aff. *D. minutus*. The petrosal preserves apparently plesiomorphic features not seen in crown marsupials. If this specimen does represent *Derorhynchus* aff. *D. minutus*, it suggests that derorhynchids probably lie outside Marsupialia. Other published phylogenetic analyses that have included *Derorhynchus* have not supported a close relationship with Paucituberculata (Goin et al. 2006; Ladevèze and Muizon 2010; Forasiepi et al. 2013). No other putative paucituberculatans have been identified at Itaboraí based on dental remains; it therefore seems likely that the femoral remains (all of which retain a third trochanter) represent stem marsupials.

Szalay (1994) identified two marsupialiform tarsal morphotypes from Itaboraí, IMGs V and XII, as representing didelphimorphians based on the presence of a bipartite calcaneocuboid joint morphology similar to that of living didelphids. The didelphid-like bipartite morphology is plausibly ancestral to the tripartite morphology seen in paucituberculatans (including argyrolagids; see above) and australidelphians (Szalay 1994; Beck 2012), in which case its reported presence in IMG V and XII does not necessarily imply that the taxa represented by these tarsal remains are members of Didelphimorphia. In any case, Szalay and Sargis (2001: 257) wrote that they now doubted that IMG V and XII represent didelphimorphians, although they did not give any details regarding this change of opinion, nor did they propose alternative affinities for these specimens. A reappraisal of these potentially highly significant specimens is desperately needed. Regardless, IMG V and XII cannot be regarded as representing unambiguous crown marsupials.

The phylogenetic analyses of Ladevèze and Muizon (2010), Oliveira and Goin (2011), and Oliveira et al. (2016) suggest that some of the Itaboraí marsupialiforms are crown marsupials, but these analyses show major conflicts both with each other and with more comprehensive metatherian phylogenies. For example, Ladevèze and Muizon (2010) found *Gaylordia* to be a crown marsupial, whereas Oliveira and Goin (2015) found it to fall outside Marsupialia, whilst the Paucituberculata + Peramelemorphia clade recovered by Ladevèze and Muizon (2010) has not been found in other recent analyses (see Beck in press-b). In summary, then, two of the best-preserved Itaboraí marsupialiforms, namely *Epidolops* and *Gaylordia*, appear to fall outside Marsupialia, and none of the remaining taxa can be confidently identified as crown marsupials.

The oldest known definitive crown marsupials are the stem australidelphian *Djarthia murgonensis* and an isolated “ameridelphian” marsupial calcaneus, both from the 54.6 Ma old Tingamarra fauna in northeasten Australia (Beck et al. 2008b; Beck 2012). The oldest unequivocal crown marsupials from South America, meanwhile are from the “Sapoan” (= 47–49 Ma old) Laguna Fría and La Barda localities in Chubut Province, southern Argentina, namely the paucitutuberculatan *Bardalestes*, microbiotherians, and isolated tarsals of an australidelphian (Goin et al. 2009; Tejedor et al. 2009; Lorente et al. 2016).

Influenced by the work of Morrone (2002, 2004, 2006), Goin and co-workers have emphasized in recent publications (Goin et al. 2007b, 2012a, 2016, in press; Lorente et al. 2016) that South America should be viewed as comprising two distinct biogeographical “kingdoms” (Goin et al. 2012a: fig. 3.1; Goin et al. 2016: fig. 4.2): northern South America is part of the Holotropical Kingdom, whilst southern South America is part of the Austral Kingdom, which also includes Antarctica and Australia. Goin et al. (2007b, 2016, in press) and Lorente et al. (2016) proposed that the origin and early evolution of Australidelphia occurred in the Austral Kingdom.

As reviewed above, unequivocal crown marsupials have not been identified at Itaboraí, which falls within the Holotropical Kingdom, whereas they are known from similarly-aged sites in the Austral Kingdom (Tingamarra, Laguna Fría, and La Barda). Definitive marsupials (paucituberculatans and microbiotherians) are known from Santa Rosa, in the Amazon Basin of eastern Peru (Goin and Candela 2004), which is part of the Holotropical Kingdom, but this site is considerably younger than Itaboraí, namely middle or late Eocene or early Oligocene. Collectively, this raises the possibility that the origin and early evolution of Marsupialia as a whole, rather than just Australidelphia, was restricted to the Austral Kingdom. This hypothesis needs to be tested by the discovery and full description of Late Cretaceous and early Paleogene mammal faunas from areas that lie within the Holotropical Kingdom (for example in the Bogotá Formation of Colombia; Bloch et al. 2012) and from the Austral Kingdom (e.g., the Las Flores and Punta Peligro faunas of southern Argentina; Goin et al. 2002; Forasiepi and Rougier 2009; Goin et al. 2016). However, it may be that much of the early evolution of marsupials occurred in regions for which the Late Cretaceous-early Paleogene fossil record of mammals is poor (Australia) or as yet non-existent (mainland Antarctica).

## Electronic supplementary material

ESM 1 (DOCX 21 kb)

ESM 2 (DOCX 164 kb)

ESM 3 (DOCX 165 kb)

ESM 4 (DOCX 161 kb)

ESM 5 (DOCX 160 kb)

ESM 6 (DOCX 37 kb)
