# Supplementary material for: The Skull of Epidolops ameghinoi from the Early Eocene Itaboraí Fauna, Southeastern Brazil, and the Affinities of the Extinct Marsupialiform Order Polydolopimorphia
Source: J Mamm Evol. 2016 Oct 26;24(4):373–414. doi: 10.1007/s10914-016-9357-6 (PMC5684316; doi:10.1007/s10914-016-9357-6)
Supplement: Supplementary file 1 — (DOCX 21 kb) [file 10914_2016_9357_MOESM1_ESM.docx]

**Supplementary Table 1.** Values for skull length represent maximum skull length (where available) or condylobasal length. Values for promontorium area are mean values from Ladevèze and Muizon (2010: table 2).

| Taxon | skull length |  | promontorium area | Source of skull length |
| --- | --- | --- | --- | --- |
| *Didelphis aurita* | 104.39 |  | 48.9375 | Cerqueira and Lemos (2000) |
| *Didelphis marsupialis* | 99.76 |  | 43.525 | Cerqueira and Lemos (2000) |
| *Didelphis albiventris* | 86.595 |  | 37.0525 | Lemos and Cerqueira (2002) |
| *Marmosa murina* | 27.31896 |  | 12.0475 | Ladevèze et al. (2011) |
| *Metachirus nudicaudatus* | 58.35 |  | 24.2 | Mares and Braun (2000) |
| *Philander opossum* | 67 |  | 26.25 | Castro-Arellano et al. (2000) |
| *Caluromys philander* | 55.3 |  | 20.6977778 | López-Fuster et al. (2008) |
| *Caenolestes fuliginosus* | 31.6 |  | 10.0371429 | Ojala-Barbour et al. (2013) |
| *Phascogale tapoatafa* | 46.02625 |  | 13.625 | Rhind et al. (2001) |
| *Pucadelphys andinus* | 28.075 |  | 8.28 | Ladevèze et al. (2011; skull length is from MHNC 8266 only) |
| *Andinodelphys cochabambensis* | 49.3125 |  | 14.834 | Muizon et al. (1997) |
| *Mayulestes ferox* | 54 |  | 20.095 | Muizon (1998) |
|  |  |  |  |  |

**References**

Castro-Arellano I, Zarza H, Medellín R (2000) *Philander opossum*. Mamm Species 638:1-8

Cerqueira R, Lemos B (2000) Morphometric differentiation between Neotropical black-eared opossums, *Didelphis marsupialis* and *D. aurita* (Didelphimorphia, Didelphidae). Mammalia 64 (3):319-327

Ladevèze S, Muizon, C. de (2010) Evidence of early evolution of Australidelphia (Metatheria, Mammalia) in South America: phylogenetic relationships of the metatherians from the Late Palaeocene of Itaborai (Brazil) based on teeth and petrosal bones. Zool J Linn Soc 159 (3):746-784. doi:DOI 10.1111/j.1096-3642.2009.00577.x

Ladevèze S, Muizon, C. de, Beck RMD, Germain D, Cespedes-Paz R (2011) Earliest evidence of mammalian social behaviour in the basal Tertiary of Bolivia. Nature 474 (7349):83-86. doi:10.1038/nature09987

Lemos B, Cerqueira R (2002) Morphological differentiation in the white-eared opossum group (Didelphidae: *Didelphis*). J Mammal 83 (2):354-369

López-Fuster MJ, Pérez-Hernández R, Ventura J (2008) Morphometrics of genus *Caluromys* (Didelphimorphia: Didelphidae) in northern South America. ORSIS 23:97-114

Mares MA, Braun JK (2000) Systematics and natural history of marsupials from Argentina. In: Choate J (ed) Reflections of a naturalist: Papers honoring Professor Eugene D. Fleharty. Fort Hays Studies, Special Issue. Stenberg Museum of Natural History, Fort Hays State University, Hays, Kansas, USA, pp 23-45

Muizon, C. de (1998) *Mayulestes ferox*, a borhyaenoid (Metatheria, Mammalia) from the early Palaeocene of Bolivia: phylogenetic and palaeobiologic implications. Geodiversitas 20 (1):19-142

Muizon, C. de, Cifelli RL, Céspedes Paz R (1997) The origin of the dog-like borhyaenoid marsupials of South America. Nature 389:486-489

Ojala-Barbour R, Pinto CM, M. JB, Albuja V. L, Lee Jr. TE, Patterson BD (2013) A new species of shrew-opossum (Paucituberculata: Caenolestidae) with a phylogeny of extant caenolestids. J Mammal 94 (5):967-982

Rhind SG, Bradley JS, Cooper NK (2001) Morphometric variation and taxonomic status of brush-tailed phascogales, *Phascogale tapoatafa* (Meyer, 1793) (Marsupialia:Dasyuridae). Aust J Zool 49:345-368
