# Supplementary material for: The Skull of Epidolops ameghinoi from the Early Eocene Itaboraí Fauna, Southeastern Brazil, and the Affinities of the Extinct Marsupialiform Order Polydolopimorphia
Source: J Mamm Evol. 2016 Oct 26;24(4):373–414. doi: 10.1007/s10914-016-9357-6 (PMC5684316; doi:10.1007/s10914-016-9357-6)
Supplement: Supplementary file 6 — (DOCX 37 kb) [file 10914_2016_9357_MOESM6_ESM.docx]

**Morphological character list**

Characters 1-258 are from Beck et al. (2014), whilst characters 259-273 are new to this study. Characters that represent plausible morphoclines/transformation series and treated as ordered are indicated. I created two versions of the morphological matrix. For Matrix A, I scored *Epidolops* based solely on specimens that preserve the dentition and so can be unequivocally identified as belonging to *E. ameghinoi*. For Matrix B, *Epidolops* was scored assuming that the Type II petrosals described by Ladevèze (2004) and the IMG VII calcanea described by Szalay (1994) also belong to this taxon (see main text). Scores for *Epidolops* are based on personal observations of craniodental specimens held at DNPM and MNRJ, plus the descriptions of Ladevèze (2004) and Szalay (1994). Scores for *Argyrolagus* were taken from Simpson (1970) and Szalay (1994), whilst scores for *Proargyrolagus* were taken from Sánchez-Villagra and Kay (1997) and Sánchez-Villagra (2001). The full morphological and total evidence matrices (including MrBayes commands) can be downloaded from Morphobank (http://www.morphobank.org, Project 2436).

1. Atlantal foramen absent (0); present (1).

***Epidolops = ?***

***Argyrolagus = ?***

***Proargyrolagus = ?***

1. Atlas transverse foramen absent (0); present (1).

***Epidolops* = ?**

***Argyrolagus* = ?**

***Proargyrolagus* = ?**

1. Atlas, posterior extent of transverse process absent (0); present (1).

***Epidolops* = ?**

***Argyrolagus* = ?**

***Proargyrolagus* = ?**

1. Atlas, cranial facets shape only concave (0); dorsal edge curved (1); dorsal edge envelops the occipital condyle medially (2). **Ordered**.

***Epidolops* = ?**

***Argyrolagus* = ?**

***Proargyrolagus* = ?**

1. Atlas, transverse process ventral to atlantal foramen groove absent (0); present (1).

***Epidolops* = ?**

***Argyrolagus* = ?**

***Proargyrolagus* = ?**

1. Atlas ventral arches open (0); complete (1).

***Epidolops* = ?**

***Argyrolagus* = 1**

***Proargyrolagus* = ?**

1. Atlas intercentrum absent (0); present with no fusion (1).

***Epidolops* = ?**

***Argyrolagus* = ?**

***Proargyrolagus* = ?**

1. Axis transverse foramen open (0); enclosed (1).

***Epidolops* = ?**

***Argyrolagus* = ?**

***Proargyrolagus* = ?**

1. Axis posterior spinous process extension absent (0); present (1).

***Epidolops* = ?**

***Argyrolagus* = ?**

***Proargyrolagus* = ?**

1. Axis extra pair of transverse processes on the ventral surface of the body absent (0); present (1).

***Epidolops* = ?**

***Argyrolagus* = ?**

***Proargyrolagus* = ?**

1. Axis anterior facets (prezygapophyses) and dens connection not linked (0); linked (1); facets extend ventral to the dens (2). **Ordered**.

***Epidolops* = ?**

***Argyrolagus* = ?**

***Proargyrolagus* = ?**

1. Suture between rib and axis is visible no (0); suture visible (1).

***Epidolops* = ?**

***Argyrolagus* = ?**

***Proargyrolagus* = ?**

1. Axis-C3-C4 fusion no fusion (0); axis and C3 fused (1); axis-C3-C4 fused (2). **Ordered**.

***Epidolops* = ?**

***Argyrolagus* = ?**

***Proargyrolagus* = ?**

1. C3-C4 ventral sagittal process absent (0); present (1).

***Epidolops* = ?**

***Argyrolagus* = ?**

***Proargyrolagus* = ?**

1. C5 transverse process displays two heads absent (0); present (1).

***Epidolops* = ?**

***Argyrolagus* = ?**

***Proargyrolagus* = ?**

1. C5 transverse process heads overlap transversally absent (0); present (1).

***Epidolops* = ?**

***Argyrolagus* = ?**

***Proargyrolagus* = ?**

1. C5 and T1 body length subequal or C5 longer than T1 (0); C5 shorter than T1 (1).

***Epidolops* = ?**

***Argyrolagus* = ?**

***Proargyrolagus* = ?**

1. C6 transverse process posterior extension absent (0); present (1).

***Epidolops* = ?**

***Argyrolagus* = ?**

***Proargyrolagus* = ?**

1. C6 spinous process absent (0); protuberance (1); lamina (2). **Ordered**.

***Epidolops* = ?**

***Argyrolagus* = ?**

***Proargyrolagus* = ?**

1. C7 transverse foramen absent (0); incipient (1); present (2). **Ordered**.

***Epidolops* = ?**

***Argyrolagus* = ?**

***Proargyrolagus* = ?**

1. Articulation among cervical vertebrae only bodies articulate (0); prezygapophyses and postzygapophyses in addition to bodies articulate (1).

***Epidolops* = ?**

***Argyrolagus* = ?**

***Proargyrolagus* = ?**

1. Articulation between C4-C5 bodies type flat (0); saddle (1).

***Epidolops* = ?**

***Argyrolagus* = ?**

***Proargyrolagus* = ?**

1. T1 transverse process absent (0); present (1).

***Epidolops* = ?**

***Argyrolagus* = ?**

***Proargyrolagus* = ?**

1. T1 transverse process level level with prezygapophysis (0); lower than prezygapophysis (1).

***Epidolops* = ?**

***Argyrolagus* = ?**

***Proargyrolagus* = ?**

1. First thoracic vertebra with a tall spinous process (relative to other vertebrae) T1 (0); T2 (1).

***Epidolops* = ?**

***Argyrolagus* = ?**

***Proargyrolagus* = ?**

1. First thoracic vertebrae with prezygapophysis facing laterally T1 (0); T2 (1); T3 (2). **Ordered**.

***Epidolops* = ?**

***Argyrolagus* = ?**

***Proargyrolagus* = ?**

1. Thoracic vertebrae, intervertebral foramen enclosed absent (0); present (1).

***Epidolops* = ?**

***Argyrolagus* = ?**

***Proargyrolagus* = ?**

1. Last lumbar vertebra, foramen on dorsal arch absent (0); present (1).

***Epidolops* = ?**

***Argyrolagus* = ?**

***Proargyrolagus* = ?**

1. Metapophyses in third lumbar vertebra anterior to last absent (0); anterior (1); dorsal (2).

***Epidolops* = ?**

***Argyrolagus* = ?**

***Proargyrolagus* = ?**

1. Caudal vertebrae, count 9 or fewer (0); 11 or more (1).

***Epidolops* = ?**

***Argyrolagus* = ?**

***Proargyrolagus* = ?**

1. Caudal vertebrae, hyperexpanded chevrons absent (0); present (1).

***Epidolops* = ?**

***Argyrolagus* = ?**

***Proargyrolagus* = ?**

1. Prehensile tail absent (0); present (1).

***Epidolops* = ?**

***Argyrolagus* = ?**

***Proargyrolagus* = ?**

1. Sternal cartilages, ossification (= sternal ribs) absent (0); present (1).

***Epidolops* = ?**

***Argyrolagus* = ?**

***Proargyrolagus* = ?**

1. Ribs, articulation with sternum with single sternebra (0); with two sternebrae (1).

***Epidolops* = ?**

***Argyrolagus* = ?**

***Proargyrolagus* = ?**

1. Coracoid process separate and large (0); hook-like process (1); small process (2); small protuberance or absent (3). **Ordered**.

***Epidolops* = ?**

***Argyrolagus* = ?**

***Proargyrolagus* = ?**

1. Ventral extension of acromion ventral (beyond) glenoid (0); does not extend beyond level of glenoid (1).

***Epidolops* = ?**

***Argyrolagus* = ?**

***Proargyrolagus* = ?**

1. Crest on medial aspect of scapula, near the caudal border absent (0); present (1).

***Epidolops* = ?**

***Argyrolagus* = ?**

***Proargyrolagus* = ?**

1. Separate large interclavicle absent (0); present (1).

***Epidolops* = ?**

***Argyrolagus* = ?**

***Proargyrolagus* = ?**

1. Infraspinous fossa width less than 1/4 its length (0); between 1/2 and 1/4 its length (1); more than 1/2 its length (2). **Ordered**.

***Epidolops* = ?**

***Argyrolagus* = ?**

***Proargyrolagus* = ?**

1. Scapular spine width at neck level narrower than infraspinous fossa (0); subequal (1); wider than infraspinous fossa (2). **Ordered**.

***Epidolops* = ?**

***Argyrolagus* = ?**

***Proargyrolagus* = ?**

1. Infraspinous/supraspinous fossa width at neck level infraspinous fossa narrower (0); subequal (1); supraspinous fossa narrower (2). **Ordered**.

***Epidolops* = ?**

***Argyrolagus* = 2**

***Proargyrolagus* = ?**

1. Humerus, medial relief for teres major m. absent (0); present (1).

***Epidolops* = ?**

***Argyrolagus* = ?**

***Proargyrolagus* = ?**

1. Humerus, lateral ridge absent (0); ridge or crest (1); massive crest continuous with deltopectoral crest (2). **Ordered**.

***Epidolops* = ?**

***Argyrolagus* = ?**

***Proargyrolagus* = ?**

1. Humerus, capitulum for radius spherical (0); cylindrical (1).

***Epidolops* = ?**

***Argyrolagus* = ?**

***Proargyrolagus* = ?**

1. Humerus, entepicondylar foramen absent (0); present (1).

***Epidolops* = ?**

***Argyrolagus* = 1**

***Proargyrolagus* = ?**

1. Humerus, olecranon fossa or foramen absent (0); small fossa (1); large fossa (2); foramen (3). **Ordered**.

***Epidolops* = ?**

***Argyrolagus* = 3**

***Proargyrolagus* = ?**

1. Humerus, laminar supinator crest absent (0); present (1).

***Epidolops* = ?**

***Argyrolagus* = 1**

***Proargyrolagus* = ?**

1. Humerus, greater tuberosity height relative to humeral head height equal or greater tuberosity is lower (0); greater tuberosity is higher (1).

***Epidolops* = ?**

***Argyrolagus* = ?**

***Proargyrolagus* = ?**

1. Humerus, extension of the deltoid crest restricted to proximal half of humerus (0); reaches distal half (1).

***Epidolops* = ?**

***Argyrolagus* = 0**

***Proargyrolagus* = ?**

1. Humerus trochlea absent (0); present (1).

***Epidolops* = ?**

***Argyrolagus* = 1**

***Proargyrolagus* = ?**

1. Humerus, proximal extension of capitulum and trochlea longer proximal extension of trochlea (0); equal (1).

***Epidolops* = ?**

***Argyrolagus* = ?**

***Proargyrolagus* = ?**

1. Humerus, medial epicondyle size small (0); large (1).

***Epidolops* = ?**

***Argyrolagus* = 0**

***Proargyrolagus* = ?**

1. Humerus distal end size small (0); large (1).

***Epidolops* = ?**

***Argyrolagus* = ?**

***Proargyrolagus* = ?**

1. Humerus, head shape symmetrical/ovoid (0); mediolaterally compressed (1).

***Epidolops* = ?**

***Argyrolagus* = ?**

***Proargyrolagus* = ?**

1. Humerus, capitulum lateral extension absent (0); present (1).

***Epidolops* = ?**

***Argyrolagus* = ?**

***Proargyrolagus* = ?**

1. Humerus, shaft curvature straight (0); curved (1).

***Epidolops* = ?**

***Argyrolagus* = ?**

***Proargyrolagus* = ?**

1. Ulna, proximal epiphysis, distal most portion of articular surface for humerus ulnar area is less than 1/2 of radial one (0); more (1).

***Epidolops* = ?**

***Argyrolagus* = ?**

***Proargyrolagus* = ?**

1. Ulna, coronoid process absent (0); present (1).

***Epidolops* = ?**

***Argyrolagus* = 1**

***Proargyrolagus* = ?**

1. Distal process of ulna spherical, contributing to a ball and socket articulation (0); nonspherical (1).

***Epidolops* = ?**

***Argyrolagus* = ?**

***Proargyrolagus* = ?**

1. Radius, articular facet for humerus circular (0); antero-posteriorly compressed (1).

***Epidolops* = ?**

***Argyrolagus* = 0**

***Proargyrolagus* = ?**

1. Lunate absent or fused to other elements (0); very small (1); relatively large, with contacts with other carpals (2). **Ordered**.

***Epidolops* = ?**

***Argyrolagus* = ?**

***Proargyrolagus* = ?**

1. Prepollex absent (0); present (1).

***Epidolops* = ?**

***Argyrolagus* = ?**

***Proargyrolagus* = ?**

1. Distolateral process of scaphoid separates dorsally lunate from magnum absent (0); present (1); present, separates dorsally lunate from magnum (2). **Ordered**.

***Epidolops* = ?**

***Argyrolagus* = ?**

***Proargyrolagus* = ?**

1. Proximal aspect of magnum single head (0); saddle-shape articulation with lunate (1).

***Epidolops* = ?**

***Argyrolagus* = ?**

***Proargyrolagus* = ?**

1. Magnum, facet with MC III flat, convex or keeled (0); fully concave (1).

***Epidolops* = ?**

***Argyrolagus* = ?**

***Proargyrolagus* = ?**

1. Trapezium, number of distal heads one head (0); two heads (1).

***Epidolops* = ?**

***Argyrolagus* = ?**

***Proargyrolagus* = ?**

1. Plane of the articular surface trapezium-Mc I in relation to other carpal-metacarpal articulations not parallel (0); somewhat parallel (1).

***Epidolops* = ?**

***Argyrolagus* = ?**

***Proargyrolagus* = ?**

1. Both MC I and MC V are hyper-reduced in relation to other metacarpals hyperreduction (0); no hyperreduction (1).

***Epidolops* = ?**

***Argyrolagus* = ?**

***Proargyrolagus* = ?**

1. Magnum and unciform proximal extension similarly proximal (0); unciform more proximal (1).

***Epidolops* = ?**

***Argyrolagus* = ?**

***Proargyrolagus* = ?**

1. Tuberosity for rectus femoris m. no relief (0); protuberance (1); depression (2).

***Epidolops* = ?**

***Argyrolagus* = ?**

***Proargyrolagus* = ?**

1. Relative size of obturator foramen smaller or equal to acetabulum (0); larger than acetabulum (1).

***Epidolops* = ?**

***Argyrolagus* = 1**

***Proargyrolagus* = ?**

1. Hyperdevelopment of iliopubic process absent (0); present (1).

***Epidolops* = ?**

***Argyrolagus* = 0**

***Proargyrolagus* = ?**

1. Pelvis, acetabular notch absent (0); present (1).

***Epidolops* = ?**

***Argyrolagus* = 1**

***Proargyrolagus* = ?**

1. Epipubic bones absent (0); present (1).

***Epidolops* = ?**

***Argyrolagus* = ?**

***Proargyrolagus* = ?**

1. Epipubic bones proximal size short (0); long (1).

***Epidolops* = ?**

***Argyrolagus* = ?**

***Proargyrolagus* = ?**

1. Mediolateral constriction in greater trochanter of femur absent (0); present (1).

***Epidolops* = ?**

***Argyrolagus* = ?**

***Proargyrolagus* = ?**

1. Relative height of greater trochanter / femoral head greater trochanter is lower or equal (0); higher (1).

***Epidolops* = ?**

***Argyrolagus* = 1**

***Proargyrolagus* = ?**

1. Femur, lesser trochanter absent (0); present (1).

***Epidolops* = ?**

***Argyrolagus* = 1**

***Proargyrolagus* = ?**

1. Fibula proximal medial accessory shelf absent (0); present (1).

***Epidolops* = ?**

***Argyrolagus* = ?**

***Proargyrolagus* = ?**

1. Fibula proximal posterior extension beyond the area of contact with the tibia does not extend posteriorly (0); extends (1).

***Epidolops* = ?**

***Argyrolagus* = ?**

***Proargyrolagus* = ?**

1. Fibula lateral distal process absent (0); present (1).

***Epidolops* = ?**

***Argyrolagus* = ?**

***Proargyrolagus* = ?**

1. Articulation between femur and fibula absent (0); present (1).

***Epidolops* = ?**

***Argyrolagus* = ?**

***Proargyrolagus* = ?**

1. Process on fibula proximal end extends proximally beyond knee absent (0); present (1).

***Epidolops* = ?**

***Argyrolagus* = 0**

***Proargyrolagus* = ?**

1. Sesamoids in articular area between tibia, fibula, and astragalus (or UAJ, upper ankle joint) absent (0); one sesamoid present (1); two sesamoids present (2). **Ordered**.

***Epidolops* = ?**

***Argyrolagus* = ?**

***Proargyrolagus* = ?**

1. Tibia medial malleolus absent (0); present (1).

***Epidolops* = ?**

***Argyrolagus* = 1**

***Proargyrolagus* = ?**

1. Tibia length relative to femur length tibia shorter than femur (0); tibia longer or equal to femur (1).

***Epidolops* = ?**

***Argyrolagus* = 1**

***Proargyrolagus* = ?**

1. Tibia proximal dimensions larger mediolaterally than anteroposteriorly (0); equal (1); smaller mediolaterally than anteroposteriorly (2). **Ordered**.

***Epidolops* = ?**

***Argyrolagus* = ?**

***Proargyrolagus* = ?**

1. Tibia posterior shelf absent (0); present but does not extend posteriorly beyond the medial astragalotibial facet (1); present and extends posteriorly beyond the medial astragalotibial facet (2). **Ordered**.

***Epidolops* = ?**

***Argyrolagus* = 0**

***Proargyrolagus* = ?**

1. Tibia, distal articulation type sagittal (0); spiral (1).

***Epidolops* = ?**

***Argyrolagus* = 0**

***Proargyrolagus* = ?**

1. Tibia, posterior shelf articulation not articular (0); articular (1).

***Epidolops* = ?**

***Argyrolagus* = -**

***Proargyrolagus* = ?**

1. Astragalus, angle between medial and lateral facets for tibia 90 degrees (0); intermediate (1); 180 degrees (2). **Ordered**.

***Epidolops* = ?**

***Argyrolagus* = 0**

***Proargyrolagus* = ?**

1. Astragalonavicular facet extends on medial side of head absent (0); medial (1).

***Epidolops* = ?**

***Argyrolagus* = 0**

***Proargyrolagus* = ?**

1. Astragalonavicular facet extends on ventromedial area of head absent (0); present (1).

***Epidolops* = ?**

***Argyrolagus* = ?**

***Proargyrolagus* = ?**

1. Astragalus, dimensions of facet for navicular in distal view transversely wider (0); dorsoventrally wider (1).

***Epidolops* = ?**

***Argyrolagus* = 0**

***Proargyrolagus* = ?**

1. Astragalar medial plantar tuberosity (ampt) visibility in dorsal view not visible (0); visible (1).

***Epidolops* = ?**

***Argyrolagus* = 0**

***Proargyrolagus* = ?**

1. Astragalus, angle between facet for fibula and lateral facet for tibia 180 or more degrees (0); intermediate (90-180 degrees) (1); 90 degrees (2). **Ordered**.

***Epidolops* = ?**

***Argyrolagus* = 0**

***Proargyrolagus* = ?**

1. Astragalar neck absent (0); present (1).

***Epidolops* = ?**

***Argyrolagus* = 1**

***Proargyrolagus* = ?**

1. Astragalus, relative width of head and neck neck narrower or as wide as head (0); neck wider than head (1).

***Epidolops* = ?**

***Argyrolagus* = ?**

***Proargyrolagus* = ?**

1. Astragalar sustentacular facet medial extent does not reach medial edge of neck (0); reaches medial edge of neck (1).

***Epidolops* = ?**

***Argyrolagus* = 0**

***Proargyrolagus* = ?**

1. Astragalonavicular facet position relative to facets for tibia anterior to facets for tibia (0); medial relative to facets for tibia (1).

***Epidolops* = ?**

***Argyrolagus* = 0**

***Proargyrolagus* = ?**

1. Astragalar canal absent (0); present (1).

***Epidolops* = ?**

***Argyrolagus* = 0**

***Proargyrolagus* = ?**

1. Ectal facet major dimension orientation straight (0); posteromedial to anterolateral (1).

***Epidolops* = ?**

***Argyrolagus* = 0**

***Proargyrolagus* = ?**

1. Ectal facet position in ventral view extending up to posterior edge of astragalus (0); subterminal (1).

***Epidolops* = ?**

***Argyrolagus* = 0**

***Proargyrolagus* = ?**

1. Astragalus, ridge between medial and lateral astragalotibial facets absent (0); present (1).

***Epidolops* = ?**

***Argyrolagus* = 1**

***Proargyrolagus* = ?**

1. Astragalus, ridge between lateral astragalotibial and astragalofibular facets absent (0); present (1).

***Epidolops* = ?**

***Argyrolagus* = 1**

***Proargyrolagus* = ?**

1. Medial astragalotibial facet, posterior extent does not reach posterior edge of lateral astragalotibial facet (0); equal in anteroposterior length as lateral astragalotibial facet (1).

***Epidolops* = ?**

***Argyrolagus* = ?**

***Proargyrolagus* = ?**

1. Astragalonavicular facet connection with sustentacular facet absent (0); present (1).

***Epidolops* = ?**

***Argyrolagus* = 1**

***Proargyrolagus* = ?**

1. Calcaneal sustentacular facet on sustentaculum no sustentaculum (facet is located in calcaneal body) (0); on sustentaculum (1); facet is located above level of sustentaculum, which becomes the medial process (2). **Ordered**.

***Epidolops* = 1**

***Argyrolagus* = 1**

***Proargyrolagus* = ?**

1. Ectal facet longest dimension anteromedial to posterolateral (0); straight (1); posteromedial to anterolateral (2). **Ordered**.

***Epidolops* = ?**

***Argyrolagus* = ?**

***Proargyrolagus* = ?**

1. Ectal facet longest axis when straight sagittally longer (0); transversely longer (1).

***Epidolops* = ?**

***Argyrolagus* = ?**

***Proargyrolagus* = ?**

1. Calcaneal anterior peroneal tubercle absent (0); present (1).

***Epidolops* = ? (Matrix A)/1 (Matrix B)**

***Argyrolagus* = 1**

***Proargyrolagus* = ?**

1. Calcaneal anterior peroneal tubercle shape protuberance (0); laminar (1); process (2). **Ordered**.

***Epidolops* = ? (Matrix A)/0 (Matrix B)**

***Argyrolagus* = 0**

***Proargyrolagus* = ?**

1. Calcaneal anterior peroneal tubercle position protruding anteriorly beyond calcaneocuboid facet (0); anterior, non-protruding (1); at a distance from anterior end of calcaneum (2). **Ordered**.

***Epidolops* = ? (Matrix A)/1 (Matrix B)**

***Argyrolagus* = 2**

***Proargyrolagus* = ?**

1. Calcaneal sustentacular facet (anterior part) mesiolateral orientation medial (0); mediodorsal or dorsal (1); anterodorsal (2).

***Epidolops* = ? (Matrix A)/0 (Matrix B)**

***Argyrolagus* = 2**

***Proargyrolagus* = ?**

1. Calcaneal sustentacular facet anteroposterior orientation dorsal (0); 45 degrees dorsoanteriorly (1).

***Epidolops* = ?**

***Argyrolagus* = 1**

***Proargyrolagus* = ?**

1. Calcaneal sustentacular facet anteroposterior convexity concave or flat (0); convex (1).

***Epidolops* = ?**

***Argyrolagus* = ?**

***Proargyrolagus* = ?**

1. Calcaneal sustentacular facet posteriorly convex absent (0); present (1).

***Epidolops* = ?**

***Argyrolagus* = ?**

***Proargyrolagus* = ?**

1. Calcaneal plantar tubercle absent (0); present (1).

***Epidolops* = ? (Matrix A)/1 (Matrix B)**

***Argyrolagus* = 1**

***Proargyrolagus* = ?**

1. Calcaneal sustentacular facet and ectal facet merging separate (0); with narrow connection (1); merged (2). **Ordered**.

***Epidolops* = ? (Matrix A)/0 (Matrix B)**

***Argyrolagus* = 0**

***Proargyrolagus* = ?**

1. Calcaneal sustentacular facet anterior edge anterior to posterior facet (0); equal or posterior to posterior facet (1).

***Epidolops* = ? (Matrix A)/0 (Matrix B)**

***Argyrolagus* = 1**

***Proargyrolagus* = ?**

1. Calcaneal facet for fibula absent (0); present, smaller than ectal facet (1); present, larger than ectal facet (2). **Ordered**.

***Epidolops* = ? (Matrix A)/1 (Matrix B)**

***Argyrolagus* = 2**

***Proargyrolagus* = ?**

1. Calcaneal facet for fibula orientation lateral (0); dorsal (1); dorsal only posteriorly (2). **Ordered**.

***Epidolops* = ?**

***Argyrolagus* = 1**

***Proargyrolagus* = ?**

1. Calcaneocuboid facet of the calcaneum planar or concave (0); subdivided into two facets (1); subdivided into three facets (2). **Ordered**.

***Epidolops* = ? (Matrix A)/0 (Matrix B)**

***Argyrolagus* = 2**

***Proargyrolagus* = ?**

1. Calcaneum sustentacular facet reaches anterior end absent (0); present (1).

***Epidolops* = ? (Matrix A)/1 (Matrix B)**

***Argyrolagus* = 0**

***Proargyrolagus* = ?**

1. Calcaneum accessory facet anterior to sustentacular facet absent (0); present (1).

***Epidolops* = ? (Matrix A)/0 (Matrix B)**

***Argyrolagus* = 0**

***Proargyrolagus* = ?**

1. Cuboid medial plantar process forms groove absent (0); present (1).

***Epidolops* = ?**

***Argyrolagus* = 0**

***Proargyrolagus* = ?**

1. Cuboidcalcaneal facet mostly convex or flat (0); concave anteroposteriorly (1); two shelves, medial more proximal (2); two concavities (3).

***Epidolops* = ?**

***Argyrolagus* = 2**

***Proargyrolagus* = ?**

1. Cuboidcalcaneal facet angle between proximal and distal facet areas no angle (0); angle present and small, dorsal area narrower than proximal area (1); angle present and almost straight, dorsal area wider than width of proximal area (2). **Ordered**.

***Epidolops* = ?**

***Argyrolagus* = 2**

***Proargyrolagus* = ?**

1. Cuboidcalcaneal facet outer shelf absent (0); present (1).

***Epidolops* = ?**

***Argyrolagus* = ?**

***Proargyrolagus* = ?**

1. Cuboidcalcaneal ventral facet absent (0); present (1).

***Epidolops* = ?**

***Argyrolagus* = 0**

***Proargyrolagus* = ?**

1. Spatial relationship between navicular and entocuneiform entocuneiform anterior to navicular (0); entocuneiform extends proximally medial to the distal area of the navicular (1).

***Epidolops* = ?**

***Argyrolagus* = ?**

***Proargyrolagus* = ?**

1. Navicular shelf between cuboid and astragalus absent (0); present (1).

***Epidolops* = ?**

***Argyrolagus* = ?**

***Proargyrolagus* = ?**

1. Navicular size navicular half to one third the size of the cuboid (0); navicular more than half the size of the cuboid to equal to it (1); navicular larger than the cuboid (2). **Ordered**.

***Epidolops* = ?**

***Argyrolagus* = 1/2**

***Proargyrolagus* = ?**

1. Mesocuneiform contact with navicular absent (0); present (1).

***Epidolops* = ?**

***Argyrolagus* = ?**

***Proargyrolagus* = ?**

1. Falcula on hallux absent (0); present (1).

***Epidolops* = ?**

***Argyrolagus* = ?**

***Proargyrolagus* = ?**

1. Prehallux absent (0); present (1).

***Epidolops* = ?**

***Argyrolagus* = ?**

***Proargyrolagus* = ?**

1. Mt IV proximal contact ectocuneiform and cuboid (0); cuboid (1).

***Epidolops* = ?**

***Argyrolagus* = ?**

***Proargyrolagus* = ?**

1. Mt V proximal process extends ventral to cuboid absent (0); present (1).

***Epidolops* = ?**

***Argyrolagus* = ?**

***Proargyrolagus* = ?**

1. Mt II and Mt III proximal ends Mt II extends more proximally than Mt III (0); equal (1); Mt III more proximal (2). **Ordered**.

***Epidolops* = ?**

***Argyrolagus* = ?**

***Proargyrolagus* = ?**

1. Hallux opposability (articulation Mt I with entocuneiform) not opposable (0); opposable (1).

***Epidolops* = ?**

***Argyrolagus* = ?**

***Proargyrolagus* = ?**

1. Ridge on proximal articular facet of Mt I absent (0); present (1).

***Epidolops* = ?**

***Argyrolagus* = ?**

***Proargyrolagus* = ?**

1. Syndactyly or external fusion of digits II and III in the foot absent (0); present (1).

***Epidolops* = ?**

***Argyrolagus* = ?**

***Proargyrolagus* = ?**

1. Mt III thickness relative to that of Mt IV Mt III thinner (0); Mt III and IV equal thickness or Mt IV thicker (1).

***Epidolops* = ?**

***Argyrolagus* = 1**

***Proargyrolagus* = ?**

1. Mt III thickness relative to that of MtI Mt III thinner (0); Mt III and I equal (1); Mt III thicker than Mt I (2); Mt I absent (3). **Ordered**.

***Epidolops* = ?**

***Argyrolagus* = ?**

***Proargyrolagus* = ?**

1. Foot ungual phalanx of digit IV, proximal view larger dorsoventrally than mediolaterally (0); equal dimensions (1); larger mediolaterally than dorsoventrally (2). **Ordered**.

***Epidolops* = ?**

***Argyrolagus* = ?**

***Proargyrolagus* = ?**

1. Number of upper incisors five (0); four (1); three (2); two (3); one (4); none (5). **Ordered**.

***Epidolops* = 2**

***Argyrolagus* = 3**

***Proargyrolagus* = 1**

1. Number of lower incisors four (0); three (1); two (2); one (3); none (4). **Ordered**.

***Epidolops* = 1**

***Argyrolagus* = 2**

***Proargyrolagus* = 0/1/2**

1. Number of upper molars four (0); three (1); none (2). **Ordered**.

***Epidolops* = 0**

***Argyrolagus* = 0**

***Proargyrolagus* = 0**

1. Upper molar M2 shape triangular or semi-triangular (0); rectangular or semi-square (1).

***Epidolops* = 1**

***Argyrolagus* = 1**

***Proargyrolagus* = 1**

1. Paracone and metacone placement in M2 medial or buccal (0); buccal margin (1).

***Epidolops* = 0**

***Argyrolagus* = ?**

***Proargyrolagus* = ?**

1. Paracone versus metacone size in M2 pa > me (0); pa = me (1); pa < me (2); pa entirely suppressed (3). **Ordered**.

***Epidolops* = ?**

***Argyrolagus* = ?**

***Proargyrolagus* = ?**

1. Centrocrista shape linear, oriented anteroposteriorly (0); V-shaped (1); linear, oriented obliquely (2).

***Epidolops* = 1**

***Argyrolagus* = ?**

***Proargyrolagus* = ?**

1. Metaconule absent or not-well developed (0); well-developed or enlarged (1).

***Epidolops* = 1**

***Argyrolagus* = 1**

***Proargyrolagus* = 1**

1. Trigonid versus talonid width trigonid wider than talonid (0); trigonid subequal to talonid or trigonid narrower than talonid (1).

***Epidolops* = 0**

***Argyrolagus* = 1**

***Proargyrolagus* = 1**

1. Paraconid on lower molars absent (0); present (1).

***Epidolops* = 0**

***Argyrolagus* = ?**

***Proargyrolagus* = 1**

1. Intersection of cristid obliqua with trigonid on m2 lingual to protocristid notch (0); labial to protocristid notch (1).

***Epidolops* = 1**

***Argyrolagus* = ?**

***Proargyrolagus* = ?**

1. Upper incisor arcade shape U-shape (0); broad V-shape (1); long, narrow V-shape (2). **Ordered**.

***Epidolops* = ?**

***Argyrolagus* = ?**

***Proargyrolagus* = ?**

1. P1 absent (0); reduced and single-rooted (1); present, well-developed (2).

***Epidolops* = 1**

***Argyrolagus* = 0**

***Proargyrolagus* = 1**

1. Lower p2 absent (0); greatly reduced (1); present (2). **Ordered**.

***Epidolops* = 1**

***Argyrolagus* = 0**

***Proargyrolagus* = 0/1**

1. Upper P2 absent (0); greatly reduced (1); present (2). **Ordered**.

***Epidolops* = 1**

***Argyrolagus* = 0**

***Proargyrolagus* = 1**

1. Upper incisors spatulate no (0); yes (1).

***Epidolops* = ?**

***Argyrolagus* = ?**

***Proargyrolagus* = ?**

1. Size upper I3 vs. I2 I3 > I2 (0); I3 = I2 (1); I3 < I2 (2). **Ordered**.

***Epidolops* = ?**

***Argyrolagus* = -**

***Proargyrolagus* = 2**

1. Procumbent gliriform lower anteriormost incisor absent (0); present (1).

***Epidolops* = 1**

***Argyrolagus* = 1**

***Proargyrolagus* = 1**

1. Hypoconulid absent or present absent (0); present (1).

***Epidolops* = 0**

***Argyrolagus* = ?**

***Proargyrolagus* = 1**

1. Lower canine absent (0); greatly reduced (1); retained (2). **Ordered**.

***Epidolops* = 2**

***Argyrolagus* = 0**

***Proargyrolagus* = 0/1**

1. Upper canine caniniform or premolariform (0); very small (1); absent (2). **Ordered**.

***Epidolops* = 0**

***Argyrolagus* = 2**

***Proargyrolagus* = 1**

1. Number of roots on upper canine two (0); one (1).

***Epidolops* = 1**

***Argyrolagus* = -**

***Proargyrolagus* = 1**

1. Lower i2 (i3 of Hershkovitz) staggered or not staggered not staggered (0); staggered (1).

***Epidolops* = 1**

***Argyrolagus* = ?**

***Proargyrolagus* = ?**

1. Bunolophodonty or lophodonty developed no (0); yes (1).

***Epidolops* = 1**

***Argyrolagus* = ?**

***Proargyrolagus* = ?**

1. Selenodonty developed no (0); yes (1).

***Epidolops* = 1**

***Argyrolagus* = ?**

***Proargyrolagus* = ?**

1. Marsupial pattern of dental replacement absent (0); present (1).

***Epidolops* = ?**

***Argyrolagus* = ?**

***Proargyrolagus* = ?**

1. Parietal-alisphenoid or squamosal-frontal contact on braincase parietal-alisphenoid (0); squamosal-frontal (1).

***Epidolops* = ?**

***Argyrolagus* = ?**

***Proargyrolagus* = ?**

1. Width of frontals versus width of parietals parietal wider or equal to frontal (0); parietal narrower than frontal (1).

***Epidolops* = ?**

***Argyrolagus* = ?**

***Proargyrolagus* = ?**

1. Angular process medially inflected no (0); yes (1).

***Epidolops* = 1**

***Argyrolagus* = 1**

***Proargyrolagus* = 1**

1. Mandibular symphysis fused no (0); yes (1).

***Epidolops* = 0**

***Argyrolagus* = 0**

***Proargyrolagus* = 0**

1. Bones surrounding infraorbital canal in the orbit maxilla + lacrimal (0); maxilla only (1).

***Epidolops* = 0**

***Argyrolagus* = ?**

***Proargyrolagus* = ?**

1. Posterior-most point of premaxillo-nasal contact anterior or at the canine (0); posterior to the canine (1).

***Epidolops* = 1**

***Argyrolagus* = -**

***Proargyrolagus* = 1**

1. Maximum maxilla (palatal portion) length/width ratio ratio less or equal to 1.5 (0); ratio larger than 1.5 (1).

***Epidolops* = ?**

***Argyrolagus* = ?**

***Proargyrolagus* = ?**

1. Maxillofrontal contact absent (0); present (1).

***Epidolops* = 1**

***Argyrolagus* = ?**

***Proargyrolagus* = 1**

1. Lacrimal tubercle absent (0); present (1).

***Epidolops* = ?**

***Argyrolagus* = ?**

***Proargyrolagus* = ?**

1. Alisphenoid tympanic wing absent (0); poorly developed (1); moderately developed (2); well-developed, extending to or near posterior lacerate foramen and paroccipital process (3). **Ordered**.

***Epidolops* = 0**

***Argyrolagus* = 3**

***Proargyrolagus* = 2**

1. Ectotympanic shape ring-shaped (0); moderately broadened (1); tubelike (2). **Ordered**.

***Epidolops* = ?**

***Argyrolagus* = ?**

***Proargyrolagus* = 0**

1. Postglenoid process absent (0); present (1).

***Epidolops* = 1**

***Argyrolagus* = ?**

***Proargyrolagus* = ?**

1. Bony external auditory meatus separates ear canal from epitympanic recess no (0); yes (1).

***Epidolops* = ?**

***Argyrolagus* = ?**

***Proargyrolagus* = ?**

1. Fusion of ectotympanic with other bones of the skull no (0); yes (1).

***Epidolops* = 0**

***Argyrolagus* = 0**

***Proargyrolagus* = 0**

1. Postglenoid foramen absent (0); present (1).

***Epidolops* = 1**

***Argyrolagus* = ?**

***Proargyrolagus* = ?**

1. Postglenoid foramen position located posterior to postglenoid process (0); even with postglenoid process (1); anterior to postglenoid process and frequently encircled by squamosal (2). **Ordered**.

***Epidolops* = 0**

***Argyrolagus* = ?**

***Proargyrolagus* = ?**

1. Position of incisura tympanica caudal or caudodorsal (0); located dorsally or anterior crus and posterior crus unite dorsally (1).

***Epidolops* = ?**

***Argyrolagus* = ?**

***Proargyrolagus* = 0**

1. Size of incisura tympanica narrow or absent (0); wide (1).

***Epidolops* = ?**

***Argyrolagus* = ?**

***Proargyrolagus* = 1**

1. Foramen ovale position lamina obturans / other bones (0); alisphenoid / petrosal (1); just alisphenoid (2); alisphenoid / squamosal (3).

***Epidolops* = ?**

***Argyrolagus* = ?**

***Proargyrolagus* = ?**

1. Carotid foramen position in basisphenoid (0); basisphenoid / basioccipital suture (1); basisphenoid / petrosal (2).

***Epidolops* = ?**

***Argyrolagus* = ?**

***Proargyrolagus* = 0**

1. Transverse canal foramen absent or vestigial (0); present (1).

***Epidolops* = 0**

***Argyrolagus* = 1**

***Proargyrolagus* = ?**

1. Transverse canal position anterior to carotid foramen (0); perforating pterygoid fossa (1); confluent with carotid foramen (2); posterior to carotid foramen (3).

***Epidolops* = ?**

***Argyrolagus* = ?**

***Proargyrolagus* = ?**

1. Intramural transverse canal absent (0); present (1).

***Epidolops* = ?**

***Argyrolagus* = ?**

***Proargyrolagus* = ?**

1. Dorsal margin of foramen magnum formed by exoccipitals and supraoccipital (0); formed by exoccipitals (1).

***Epidolops* = ?**

***Argyrolagus* = ?**

***Proargyrolagus* = ?**

1. Shape of nasals posteriorly expanded (0); not posteriorly expanded (1).

***Epidolops* = ?**

***Argyrolagus* = 1**

***Proargyrolagus* = 1**

1. Septomaxilla absent (0); present (1).

***Epidolops* = 0**

***Argyrolagus* = ?**

***Proargyrolagus* = 0**

1. Palatal vacuities absent or just small foramina (0); present, restricted to palatine bones (1); present in both palatine and maxillary bones (2). **Ordered**.

***Epidolops* = 0**

***Argyrolagus* = 2**

***Proargyrolagus* = 2**

1. Premaxilla, palatal process does not (0); does reach canine alveolus or it is immediately posterior to it (1).

***Epidolops* = 1**

***Argyrolagus* = -**

***Proargyrolagus* = ?**

1. Minor palatine foramen absent (0); present (1).

***Epidolops* = 1**

***Argyrolagus* = ?**

***Proargyrolagus* = ?**

1. Hypoglossal foramina confluent with jugular foramen (0); one (1); two or more (2). **Ordered**.

***Epidolops* = ?**

***Argyrolagus* = ?**

***Proargyrolagus* = 2**

1. Optic foramen absent (0); present (1).

***Epidolops* = ?**

***Argyrolagus* = ?**

***Proargyrolagus* = ?**

1. masseteric fossa imperforate (0); masseteric foramen or foramina present (1); massseteric canal present (2).

***Epidolops* = 1**

***Argyrolagus* = 1**

***Proargyrolagus* = ?**

1. Malleolar neck long relative to head and manubrium (0); short (1).

***Epidolops* = ?**

***Argyrolagus* = ?**

***Proargyrolagus* = ?**

1. Ossicular axis > 20 degrees (0); 10 to 20 degrees (1); < 10 degrees (2). **Ordered**.

***Epidolops* = ?**

***Argyrolagus* = ?**

***Proargyrolagus* = ?**

1. Manubrial-incudal lever-arm ratio equal or smaller than 1.6 (0); larger than 1.6 (1).

***Epidolops* = ?**

***Argyrolagus* = ?**

***Proargyrolagus* = ?**

1. Stapedial ratio < 1.8 (0); > 1.8 (1).

***Epidolops* = ?**

***Argyrolagus* = ?**

***Proargyrolagus* = ?**

1. Stapedial foramen visible absent (0); present (1).

***Epidolops* = ?**

***Argyrolagus* = ?**

***Proargyrolagus* = ?**

1. Bullate stapes not bullate (0); bullate (1).

***Epidolops* = ?**

***Argyrolagus* = ?**

***Proargyrolagus* = ?**

1. Paraseptal cartilage dips down vertically by the side of nasopalatine duct no (0); yes (1).

***Epidolops* = ?**

***Argyrolagus* = ?**

***Proargyrolagus* = ?**

1. Paraseptal cartilage shape outer bar connects with uppermost portion of paraseptal cartilage (0); with middle portion, dorsal process short (1); with middle portion, dorsal process long (2).

***Epidolops* = ?**

***Argyrolagus* = ?**

***Proargyrolagus* = ?**

1. Portion of paraseptal cartilage is ring-shaped in cross section no (0); yes (1).

***Epidolops* = ?**

***Argyrolagus* = ?**

***Proargyrolagus* = ?**

1. Sperm pairing in epididymis no (0); yes (1).

***Epidolops* = ?**

***Argyrolagus* = ?**

***Proargyrolagus* = ?**

1. Pouch type in mammary area type 1 (0); type 5 (1); type 6 (2); no marsupium or skin folds develop during the reproductive period (3); thin marsupium-like structure develops during reproductive period (4).

***Epidolops* = ?**

***Argyrolagus* = ?**

***Proargyrolagus* = ?**

1. Mammary count 0 teats (0); 2 teats (1); 4 teats (2); 5-8 teats (3); 9 teats or more (4). **Ordered**.

***Epidolops* = ?**

***Argyrolagus* = ?**

***Proargyrolagus* = ?**

1. Caecum absent (0); present (1).

***Epidolops* = ?**

***Argyrolagus* = ?**

***Proargyrolagus* = ?**

1. Fasciculus aberrans in brain absent (0); present (1).

***Epidolops* = ?**

***Argyrolagus* = ?**

***Proargyrolagus* = ?**

1. Cavum supracochleare is not roofed dorsally by the petrosal, and a depression in the anterior lamina for the geniculate ganglion is visible in dorsal view (0); is entirely enclosed within the petrosal (1); or is not floored ventrally, such that there is no secondary facial foramen (2).

***Epidolops* = ? (Matrix A)/0 (Matrix B)**

***Argyrolagus* = ?**

***Proargyrolagus* = ?**

1. Cavum epiptericum floored by petrosal (0); petrosal and alisphenoid (1); primarily or exclusively by alisphenoid (2); primarily open as piriform fenestra (3).

***Epidolops* = ?**

***Argyrolagus* = ?**

***Proargyrolagus* = ?**

1. Fossa subarcuata deep (0); extremely shallow (1).

***Epidolops* = ? (Matrix A)/0 (Matrix B)**

***Argyrolagus* = ?**

***Proargyrolagus* = ?**

1. Pars mastoidea not extensively pneumatised (0); extensively pneumatised and composed of cancellous bone (1).

***Epidolops* = ? (Matrix A)/0 (Matrix B)**

***Argyrolagus* = ?**

***Proargyrolagus* = ?**

1. Expansion of the crista petrosa into a salient crest that may cover the anterolateral part of the fossa subarcuata absent (0); present (1).

***Epidolops* = ?**

***Argyrolagus* = ?**

***Proargyrolagus* = ?**

1. Anterior lamina of petrosal exposure on the lateral wall of the braincase present and large (0); rudimentary (1); absent (2).

***Epidolops* = ? (Matrix A)/2 (Matrix B)**

***Argyrolagus* = ?**

***Proargyrolagus* = ?**

1. Anterior lamina of petrosal makes a major contribution to the medial wall of the middle cranial fossa (0); makes a minor contribution (1).

***Epidolops* = ?**

***Argyrolagus* = ?**

***Proargyrolagus* = ?**

1. Internal acoustic meatus with prefacial commissure at least 50% the width of the internal acoustic meatus (0); with prefacial commissure less than 50% the width of the internal acoustic meatus (1).

***Epidolops* = ?**

***Argyrolagus* = ?**

***Proargyrolagus* = ?**

1. Deep groove for internal carotid artery excavated on anterior pole of promontorium absent (0); present (1).

***Epidolops* = ? (Matrix A)/1 (Matrix B)**

***Argyrolagus* = ?**

***Proargyrolagus* = ?**

1. Deep and large fossa for the tensor tympani muscle excavated on the anterolateral aspect of promontorium, creating a battered ventral surface of the promontorium absent (0); present (1).

***Epidolops* = ? (Matrix A)/1 (Matrix B)**

***Argyrolagus* = ?**

***Proargyrolagus* = ?**

1. Epitympanic wing of petrosal (1) absent (0); present (1).

***Epidolops* = ? (Matrix A)/1 (Matrix B)**

***Argyrolagus* = ?**

***Proargyrolagus* = ?**

1. Epitympanic wing of petrosal (2) flat (0); confluent with bulla (1).

***Epidolops* = ? (Matrix A)/0 (Matrix B)**

***Argyrolagus* = ?**

***Proargyrolagus* = ?**

1. Lateral flange large and lateral to promontorium (0); greatly reduced or absent (1).

***Epidolops* = ? (Matrix A)/1 (Matrix B)**

***Argyrolagus* = ?**

***Proargyrolagus* = ?**

1. Broad shelf of bone surrounding fenestra cochleae and making a separation between it and aqueductus cochleae absent (0); present (1).

***Epidolops* = ? (Matrix A)/0 (Matrix B)**

***Argyrolagus* = ?**

***Proargyrolagus* = ?**

1. Rostral tympanic process of petrosal (1) absent (0); present as a distinct crest or erected process (1).

***Epidolops* = ? (Matrix A)/0 (Matrix B)**

***Argyrolagus* = 1**

***Proargyrolagus* = 1**

1. Rostral tympanic process of petrosal (2) forms an anterolaterally directed wing, sometimes contacting the ectotympanic, that does not extend on the whole length of the promontorium (0); that does extend on the whole length of the promontorium (1).

***Epidolops* = ? (Matrix A)/- (Matrix B)**

***Argyrolagus* = 1**

***Proargyrolagus* = 1**

1. Tympanic aperture of hiatus Fallopii dorsal (0); intermediate (1); ventral (2). **Ordered**.

***Epidolops* = ? (Matrix A)/0 (Matrix B)**

***Argyrolagus* = ?**

***Proargyrolagus* = ?**

1. Stylomastoid foramen absent (0); present (1).

***Epidolops* = ?**

***Argyrolagus* = ?**

***Proargyrolagus* = ?**

1. Inferior petrosal sinus intrapetrosal (0); between petrosal, basisphenoid and basioccipital (1); endocranial (2).

***Epidolops* = ? (Matrix A)/1 (Matrix B)**

***Argyrolagus* = ?**

***Proargyrolagus* = ?**

1. Mastoid exposure contacts the parietal (0); does not contact the parietal (1); greatly reduced, forming a dorsoventrally narrow band on the ventrolateral corner of the posterior face of the cranium (2).

***Epidolops* = ?**

***Argyrolagus* = ?**

***Proargyrolagus* = ?**

1. Mastoid tympanic process large and vertical (0); small, slanted, and nodelike, on the posterolateral border of the stylomastoid notch and continuous with squamosal (1); indistinct to absent (2).

***Epidolops* = ? (Matrix A)/1 (Matrix B)**

***Argyrolagus* = ?**

***Proargyrolagus* = ?**

1. Caudal tympanic process of petrosal (1) absent (0); present (1).

***Epidolops* = ? (Matrix A)/1 (Matrix B)**

***Argyrolagus* = ?**

***Proargyrolagus* = ?**

1. Caudal tympanic process of petrosal (2) forms a small crest that does not wholly floor the postpromontorial sinus (0); forms an expanded lamina that floors the postpromontorial sinus (1).

***Epidolops* = ? (Matrix A)/0 (Matrix B)**

***Argyrolagus* = ?**

***Proargyrolagus* = ?**

1. Petrosal plate absent (0); present (1).

***Epidolops* = ? (Matrix A)/0 (Matrix B)**

***Argyrolagus* = ?**

***Proargyrolagus* = ?**

1. Fossa incudis and epitympanic recess continuous (0); separated by a distinct ridge (1).

***Epidolops* = ?**

***Argyrolagus* = ?**

***Proargyrolagus* = ?**

1. Petrosal crest absent (0); present (1).

***Epidolops* = ? (Matrix A)/1 (Matrix B)**

***Argyrolagus* = ?**

***Proargyrolagus* = ?**

1. Petrosal contribution to the lateral wall of the epitympanic recess (1) absent (0); present (1).

***Epidolops* = ? (Matrix A)/1 (Matrix B)**

***Argyrolagus* = ?**

***Proargyrolagus* = ?**

1. Petrosal contribution to the lateral wall of the epitympanic recess (2) massive, large shelf of bone, sometimes rounded (0); slender and triangular (1); forming a thin lamina (2).

***Epidolops* = ? (Matrix A)/1 (Matrix B)**

***Argyrolagus* = ?**

***Proargyrolagus* = ?**

1. Prootic canal present (0); absent (1).

***Epidolops* = ? (Matrix A)/0 (Matrix B)**

***Argyrolagus* = ?**

***Proargyrolagus* = ?**

1. Imprint of the transverse sinus bifurcation on the petrosal absent (0); present (1).

***Epidolops* = ? (Matrix A)/0 (Matrix B)**

***Argyrolagus* = ?**

***Proargyrolagus* = ?**

1. Foramina on the sigmoid sinus and/or prootic sinus, apparently connecting both vessels (i.e., sigmoid sinus vein) absent (0); present (1).

***Epidolops* = ? (Matrix A)/0 (Matrix B)**

***Argyrolagus* = ?**

***Proargyrolagus* = ?**

1. Posttemporal sulcus on the squamosal surface of the petrosal present (0); absent (1).

***Epidolops* = ? (Matrix A)/0 (Matrix B)**

***Argyrolagus* = ?**

***Proargyrolagus* = ?**

1. Posttemporal notch/foramen present (0); absent (1).

***Epidolops* = ? (Matrix A)/0 (Matrix B)**

***Argyrolagus* = ?**

***Proargyrolagus* = ?**

1. Transpromontorial sulcus present (0); absent (1).

***Epidolops* = ? (Matrix A)/1 (Matrix B)**

***Argyrolagus* = ?**

***Proargyrolagus* = ?**

1. Sulcus for stapedial artery present (0); absent (1).

***Epidolops* = ? (Matrix A)/1 (Matrix B)**

***Argyrolagus* = ?**

***Proargyrolagus* = ?**

1. Cochlear coiling absent or less than 300° (0); fully coiled (more than 360°) (1).

***Epidolops* = ? (Matrix A)/1 (Matrix B)**

***Argyrolagus* = ?**

***Proargyrolagus* = ?**

1. Squamosal contribution to hypotympanic sinus roof absent (0); present (1).

***Epidolops* = 0**

***Argyrolagus* = ?**

***Proargyrolagus* = ?**

1. Alisphenoid contribution to hypotympanic sinus roof absent (0); present (1).

***Epidolops* = 1**

***Argyrolagus* = ?**

***Proargyrolagus* = ?**

1. Posterior cingulid on lower molars absent (0); present (1).

***Epidolops* = ?**

***Argyrolagus* = ?**

***Proargyrolagus* = 0**

1. Distinct third trochanter present (0); absent (1).

***Epidolops* = ?**

***Argyrolagus* = 0**

***Proargyrolagus* = ?**

1. Squamosal epitympanic sinus absent (0); present (1).

***Epidolops* = ?**

***Argyrolagus* = ?**

***Proargyrolagus* = ?**

1. NEW. Masseteric process absent (0); present (1).

***Epidolops* = 0**

***Argyrolagus* = 1**

***Proargyrolagus* = ?**

1. NEW. Retrodental foramen absent (0); present (1).

***Epidolops* = 0**

***Argyrolagus* = 1**

***Proargyrolagus* = 1**

1. NEW. Molars non-hypsodont (0); hypsodont (1); hypselodont (2). **Ordered**.

***Epidolops* = 0**

***Argyrolagus* = 2**

***Proargyrolagus* = 1**

1. NEW. Glenoid fossa planar (0); with distinct articular eminence and mandibular fossa (1); convex (2).

***Epidolops* = 0**

***Argyrolagus* = 0**

***Proargyrolagus* = 0**

1. NEW. Groove for tendon of peroneus longus muscle on dorsal surface of peroneal process (0); on ventral surface of peroneal process (1).

***Epidolops* = 1**

***Argyrolagus* = 0**

***Proargyrolagus* = ?**

1. NEW. Ectal and fibular facets confluent (0); separate (1).

***Epidolops* = 0**

***Argyrolagus* = 1**

***Proargyrolagus* = ?**

1. NEW. Paracanine fossa present (0); absent (1).

***Epidolops* = 1**

***Argyrolagus* = 1**

***Proargyrolagus* = 1**

1. NEW. Lower canine procumbency erect (0); procumbent (1).

***Epidolops* = 1**

***Argyrolagus* = -**

***Proargyrolagus* = ?**

1. NEW. Antorbital fossa absent (0); present (1).

***Epidolops* = 0**

***Argyrolagus* = ?**

***Proargyrolagus* = 1**

1. NEW. Maxillo-nasal contact present (0); prevented by premaxilla-frontal contact (1).

***Epidolops* = 0**

***Argyrolagus* = ?**

***Proargyrolagus* = 1**

1. NEW. Antorbital fenestra absent (0); present (1).

***Epidolops* = 0**

***Argyrolagus* = 0**

***Proargyrolagus* = 0**

1. NEW. P3 present (0); absent (1).

***Epidolops* = 0**

***Argyrolagus* = 0**

***Proargyrolagus* = 0**

1. NEW. P3 morphology premolariform (0); hypsodont or hypselodont (1); bladelike (2).

***Epidolops* = 2**

***Argyrolagus* = 1**

***Proargyrolagus* = 1**

1. NEW. Protocone absent or vestigial (0); present (1).

***Epidolops* = 1**

***Argyrolagus* = 1**

***Proargyrolagus* = 1**

1. NEW. Talonid absent or vestigial (0); present (1).

***Epidolops* = 1**

***Argyrolagus* = 1**

***Proargyrolagus* = 1**

**References**

Beck RMD, Travouillon KJ, Aplin KP, Godthelp H, Archer M (2014) The osteology and systematics of the enigmatic Australian Oligo-Miocene metatherian *Yalkaparidon* (Yalkaparidontidae; Yalkaparidontia; ?Australidelphia; Marsupialia). J Mamm Evol 21 (2):127-172

Ladevèze S (2004) Metatherian petrosals from the Late Paleocene of Itaboraí (Brazil), and their phylogenetic implications. J Vertebr Paleontol 24:202-213

Sánchez-Villagra MR (2001) The phylogenetic relationships of argyrolagid marsupials. Zool J Linn Soc 131:481-496

Sánchez-Villagra MR, Kay RF (1997) A skull of *Proargyrolagus*, the oldest argyrolagid (Late Oligocene Salla Beds, Bolivia), with brief comments concerning its paleobiology. J Vertebr Paleontol 17 (4):717-724

Simpson GG (1970) The Argyrolagidae, extinct South American marsupials. Bull Mus Comp Zool 139:1-86

Szalay FS (1994) Evolutionary history of the marsupials and an analysis of osteological characters. Cambridge University Press, Cambridge
